# Characteristics and multi-omics analysis of spontaneous spondyloarthritis in non-human primates: Case report

**DOI:** 10.1016/j.heliyon.2025.e41706

**Published:** 2025-01-08

**Authors:** Lei Cai, Qing Lv, Ronghua Ma, Wei Liu, Yalun Guan, Zhongqiang Huang, Qingyu Liu, Yunfeng Li, Shuhua Liu, Ge Li, Yu Zhang

**Affiliations:** a Guangdong Provincial Biotechnology Research Institute (Guangdong Provincial Laboratory Animals Monitoring Center), Guangzhou, Guangdong, 510663, China; b Department of Rheumatology and Immunology, The seventh affiliated hospital, Sun Yat-sen University, Guangzhou, 518107, China; c Huazhen Laboratoty Animal Breeding Center, Guangzhou, Guangdong, 510900, China; d Guangzhou Huazhen Biosciences Co., Ltd., Guangzhou, Guangdong, 510900, China; e Radiological Department, The seventh affiliated hospital, Sun Yat-sen University, Guangzhou, 518107, China; f Guangzhou National Laboratory, Guangzhou, 510005, China

**Keywords:** Macaca fascicularis, Spondyloarthritis, MRI, Transcriptome, Proteome, S100A8/A9

## Abstract

Spondyloarthritis is a prevalent and persistent condition that significantly impacts the quality of life. Its intricate pathological mechanisms have led to a scarcity of animal models capable of replicating the disease progression in humans, making it a prominent area of research interest in the field. To delve into the pathological and physiological traits of spontaneous non-human primate spondyloarthritis, this study meticulously examined the disease features of this natural disease model through an array of techniques including X-ray imaging, MRI imaging, blood biochemistry, markers of bone metabolism, transcriptomics, proteomics, and metabolomics. X-ray imaging results revealed that crab-eating monkeys (*Macaca fascicularis*) with spontaneous spondyloarthritis developed bone spurs in the spine and experienced bone destruction in peripheral joints. MRI imaging further confirmed inflammatory changes in the spine and facet joints of these monkeys, along with inflammation and bone destruction in peripheral joints. Blood biochemistry analysis showed abnormalities in liver function and kidney function indicators in crab-eating monkeys with spontaneous spondyloarthritis. Analysis of bone metabolism-related markers showed a decrease in bone formation (BGP) and bone resorption (β-CTx). A thorough correlation analysis was conducted on the transcriptome and proteome expression data, revealing a significant positive correlation between the two datasets. A total of eight genes and proteins with high expression levels were identified as common to both the transcriptome and proteome. Subsequent Gene Ontology (GO) and Kyoto Encyclopedia of Genes and Genomes (KEGG) analyses were performed on these co-expressed genes and proteins, indicating a predominant enrichment in the IL-17 signaling pathway, with S100A8 and S100A9 identified as the core proteins. Further analysis using clinical data in conjunction with proteome data through Weighted Gene Co-expression Network Analysis (WGCNA) demonstrated a significant positive correlation between the high expression of S100A8 and S100A9 and the clinical phenotypes of spinal abnormalities, thereby corroborating the close association of S100A8 and S100A9 with the phenotype of spondyloarthritis. Human clinical studies have already established a link between S100A8 and S100A9 and autoimmune-related arthritic diseases, suggesting that the spontaneous spondyloarthritis observed in crab-eating macaques may be related to autoimmune diseases. It is hypothesized that S100A8 and S100A9 could serve as potential predictive biomarkers for spondyloarthritis in non-human primates.

## 1 Introduction

Spondyloarthritis is a group of diseases with similar clinical, genetic, and pathophysiological characteristics [1], including ankylosing spondylitis, reactive arthritis, psoriatic arthritis, inflammatory bowel disease-related arthritis, undifferentiated spondyloarthritis, and juvenile chronic arthritis. According to epidemiological statistics, the prevalence of this disease can be as high as 2.5 % [2]. In a survey of the Chinese military population, the prevalence was 0.45 %, and in a civilian survey, it was 0.93 % [3]. Spondyloarthritis has become a very common and chronic disease that significantly impacts quality of life.

The pathogenesis of Spondyloarthritis is complex, involving multiple factors such as genetic factors, abnormalities in the immune system, and microbial infections [[4], [5], [6], [7]]. Analysis of genome-wide association studies (GWAS) has identified several genetic loci that may be associated with spondyloarthritis, primarily involving the immune system, cell signaling pathways, and bone metabolism [8]. Patients with spondyloarthritis have abnormal immune cells and immune molecules in their peripheral blood, such as T lymphocytes, B lymphocytes, macrophages, and changes in cytokines like interleukin-1, interleukin-6, and tumor necrosis factor (TNF)-α [9,10]. In recent years, more and more studies have shown that microbial infections may be associated with the pathogenesis of spondyloarthritis, with infections like Epstein-Barr virus, mycoplasma, and chlamydia, which may play a role in the onset and progression of the disease [11].

In the study of spondyloarthritis animal models, various rodent models have been developed, including transgenic animal models [12], enthesitis-stiff animal models [13], inflammation-driven animal models [14], etc. However, most animal models are difficult to accurately simulate the pathogenesis of human spondyloarthritis. Non-human primates, as animals with the closest genetic relationship to humans, are excellent models for simulating the development of complex human diseases. Currently, there are only a few reports on spontaneous spondyloarthritis in non-human primates such as rhesus monkeys and crab-eating monkeys [15,16], mostly focusing on observations or phenotypes [17,18]. There is limited research on the imaging characteristics, blood biochemical characteristics, blood omics characteristics, etc. of spontaneous spondyloarthritis in non-human primates, and a lack of effective exploration of the specific pathogenic mechanisms, which hinders the expansion of the use of such spontaneous animal models for studying the pathogenesis of the disease and screening and evaluating drugs.

In this study, we initially identified individuals with spontaneously occurring spondyloarthritis in a captive population of macaques in Guangzhou, China. Through extensive screening of the breeding population, we found that the incidence of spontaneous spondyloarthritis was approximately 1 % of the total population, with onset occurring at around 13 years of age. The clinical manifestations of the disease were similar to those of Spondyloarthritis in humans. To further understand the characteristics of spondyloarthritis in these macaques and explore the underlying mechanisms of the disease, we utilized various techniques including X-ray imaging, MRI imaging, blood biochemistry, bone turnover markers, transcriptomics, and proteomics. Our study systematically investigated the disease features of spontaneously occurring spondyloarthritis in macaques, with the aim of expanding its applications in biomedical research.

## 2 Methods

### 2.1 Ethics and animal groups

The crab-eating monkeys used in this study were all from the Huazhen Animal Breeding Farm in Guangzhou City. All animals were group-housed, with a temperature of 18∼26 °C, relative humidity of 40 %–70 %, and were fed regularly with natural light. Thirteen crab-eating monkeys with abnormal spinal joint were identified through gross observation and X-ray screening, consisting of 7 females and 6 males, with an average body weight of 4.22 ± 1.22 kg and an average age of 11.45 ± 4.05 years. Additionally, 10 normal crab-eating monkeys with similar age and body weight were selected as the control group, with 5 females in each group. The basic information of the animals used in this study is shown in Table 1. This study was approved by the Institutional Animal Care and Use Committee (IACUC2021147) of the Guangdong Experimental Animal Monitoring Institute.

**Table 1:** Basic information of animals.

Animal ID	Gender	Body weight	Age	Group
1	♀	5.6	14	SpAF (spondyloarthritis female group)
2	♀	2.9	8.3	
3	♀	4	13.4	
4	♀	2.6	10.2	
5	♀	5	10.9	
6	♀	2.5	8.6	
7	♀	3.7	18.7	
8	♂	3.3	7.9	SpAF (spondyloarthritis male group)
9	♂	6.6	20.5	
10	♂	5.1	11.1	
11	♂	5.4	7.3	
12	♂	3.6	7.2	
13	♂	4.5	10.7	
14	♂	8.3	7.4	Ctrl F (normal female animal)
15	♂	8	7.5	
16	♂	8.4	7.5	
17	♂	8.2	7.4	
18	♂	6.1	7.4	
19	♀	4.6	14.6	Ctrl M (normal male animal)
20	♀	5.2	14.3	
21	♀	3.5	10.7	
22	♀	4.3	10	
23	♀	3.9	14.1	

### 2.2 Radiographic examination of animals

The experimental monkeys were anesthetized before undergoing X-rays using a Mikasa HF400VA X-ray machine. The X-rays included a full spine anterior/lateral view and a pelvic anterior view. In the spine anterior/lateral view, the monkey's mandible should be visible at the top and the tail end at the bottom. The pelvic anterior view should show the tip of the coccyx aligned with the pubic symphysis, with both closed rings and iliac wings in a symmetrical posture. Additionally, a nuclear magnetic resonance imaging device (uMR® 790: 3.0T MR, United-Imaging Healthcare) was used to further examine the spines of one normal monkey and three typical scoliotic crab-eating monkeys.

### 2.3 Sample collection

During the X-ray examination, 3 mL of whole blood is collected per animal, with 1 mL being used to separate peripheral blood mononuclear cells (PBMC) using lymphocyte separation fluid (Biosharp, BL590) and stored in Trizol at −80 °C for transcriptome sequencing. The remaining blood is separated using centrifugation to obtain serum, which is stored at −80 °C for testing blood biochemistry, bone metabolism indicators, and proteomics.

### 2.4 Hematological parameters testing

The serum of control group and diseased group of crab-eating macaques was tested using the fully automated blood biochemistry analyzer (HITACHI 3100), including 16 blood biochemical indicators such as alanine aminotransferase (ALT), aspartate aminotransferase (AST), alkaline phosphatase (ALP), as well as 5 trace element indicators including calcium (Ca), phosphorus (P), sodium (Na), potassium (K), and chloride (Cl).

Bone metabolism-related markers, such as bone-specific alkaline phosphatase (BAP), osteocalcin (BGP), and total type I collagen amino-terminal propeptide (PINP), are analyzed using electrochemiluminescence or LC-MS/MS techniques. In addition, markers for bone formation (such as parathyroid hormone) and bone resorption (such as 25-hydroxyvitamin D and beta-collagen degradation products) are also measured. LC-MS/MS is utilized for assessing 25-hydroxyvitamin D, while electrochemiluminescence is employed for the other markers.

### 2.5 Transcriptome sequencing

After total RNA extraction and library construction of PBMC cells, transcriptome sequencing of 23 samples was performed using the DNASEQ sequencing platform. The sequencing data was filtered, quality assessed, aligned to the reference genome of the rhesus macaque, and differential gene expression was identified. Differential gene expression analysis included GO, KEGG, clustering, gene variation, and other analyses. The specific sequencing work was completed by BGI Genomics Co., Ltd. in Shenzhen.

### 2.6 Proteome sequencing

Proteomics sequencing was conducted using a non-targeted protein quantification technique. Samples were subjected to total protein extraction, protein concentration determination, trypsin digestion, liquid chromatography-mass spectrometry analysis, and bioinformatics analysis to identify differentially expressed proteins. The differentially expressed proteins were then subjected to GO functional clustering analysis, KEGG enrichment analysis, protein domain enrichment analysis, and protein interaction network analysis. The specific sequencing work was completed by BGI Genomics Co., Ltd. in Shenzhen.

### 2.7 Transcriptome and proteome correlation analysis

Select differentially expressed genes from the transcriptome and differentially expressed proteins from the proteome, and carry out the following analyses separately: (1) Differential quantitative analysis: correlation quantity statistics, expression level correlation; (2) Functional enrichment analysis: GO enrichment, Pathway enrichment; (3) Transcription factor analysis: TF statistical analysis, TF expression level analysis; (4) Network correlation analysis: Pathway network, protein interaction network.

### 2.8 Clinical and proteomic datasets were integrated through weighted gene Co-expression network analysis (WGCNA)

Clinical observations regarding the rate of spinal abnormalities (Table 2) and the binary classification of spinal status (coded as 1 for abnormal and 0 for normal) were analyzed alongside protein expression data using Weighted Gene Co-expression Network Analysis (WGCNA) [19]: (1) The correlation coefficient was employed to measure co-expression, capitalizing on the interaction patterns among genes and constructing a gene co-expression network based on weighted expression correlations. (2) Hierarchical clustering was performed based on the weighted correlations, and the clustering results were dissected using the dynamic tree-cutting method according to set criteria, yielding distinct gene modules represented by branches and colors on a dendrogram. (3)The proteins within these modules were correlated with external phenotypic trait information to identify biologically significant clusters associated with phenotypic traits. Modules of interest were further analyzed to uncover potential key drivers within them.

**Table 2:** Statistics of spine abnormalities.

Animal ID	Number of vertebrae	Abnormal number of vertebrae	Abnormality rate/%	Abnormality	Group
1	11	8	73	1	SpAF (spondyloarthritis female group)
2	16	15	94	1	
3	15	11	73	1	
4	16	9	56	1	
5	12	12	100	1	
6	16	10	63	1	
7	13	13	100	1	
8	18	18	100	1	SpAM (spondyloarthritis male group)
9	15	15	100	1	
10	16	16	100	1	
11	15	15	100	1	
12	16	16	100	1	
13	13	13	100	1	
14	12	0	0	0	Ctrl F (normal female animal)
15	15	0	0	0	
16	11	0	0	0	
17	14	0	0	0	
18	17	0	0	0	
19	16	0	0	0	Ctrl M (normal male animal)
20	14	0	0	0	
21	19	0	0	0	
22	15	0	0	0	
23	16	0	0	0	

### 2.9 Data analysis

Statistical analysis of the results was conducted using SPSS 17.0, with a two-tailed *t*-test used to calculate the significance of differences between groups. A p-value of less than 0.05 was considered statistically significant.

## 3 Results

### 3.1 Imaging features of spontaneous spondyloarthropathy in crab-eating macaques

**Fig. 1:**
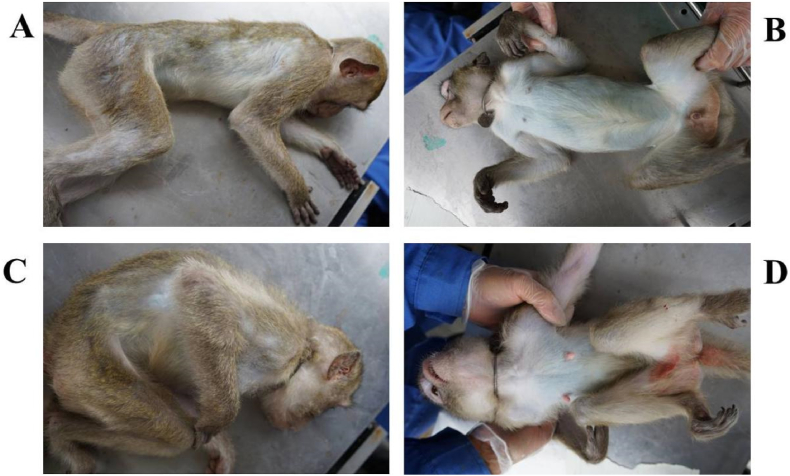
The external characteristics of the spinal arthritis in the crab-eating macaque. Note: A and B are ordinary animals, C and D are spondyloarthropathy animals.

According to statistics, about 1 % of the breeding group of macaques spontaneously develop spinal arthritis. The diseased animals show symptoms such as emaciation, restricted movement, stiffness in the lower limb and muscl atrophy, limping, hunchback, and decreased joint mobility in all four limbs (Fig. 1A-1D).

**Fig. 2:**
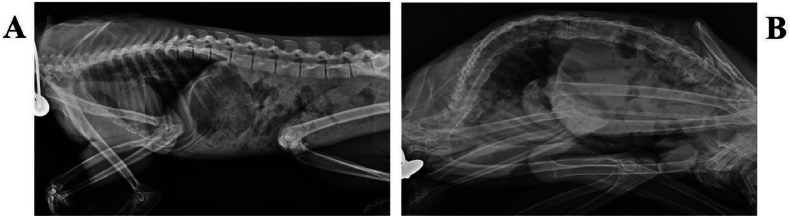
X-ray imaging characteristics of spinal spondyloarthritis for eating crab monkeys. Note: A is ordinary animal, B is spondyloarthropathy animal.

The X-ray examination results reveal several findings: (1) Spine: changing in the natural curvature of the spine with vertebral malformations, osteophyte changes along vertebral edges, and evidence of fusion. When analyzing the abnormal vertebrae, the rate of abnormality in the diseased group is consistently over 60 %. Refer to Table 2 for specific abnormality rates. (2) Peripheral joints: narrowing of joint spaces, osteoporosis, and bone destruction in the knee, elbow, hand, interphalangeal, and metatarsophalangeal joints. (3) Pelvis: The bone structure and morphology of the pelvis appear normal without any abnormalities. The bilateral sacroiliac joint surfaces are smooth with no evidence of roughness or narrowing of joint spaces. The hip joint appear normal. Overall, there is a consideration of bone spurs forming in the spine and bone destruction in the peripheral joints, as depicted in Fig. 2A-2B.

**Fig. 3:**
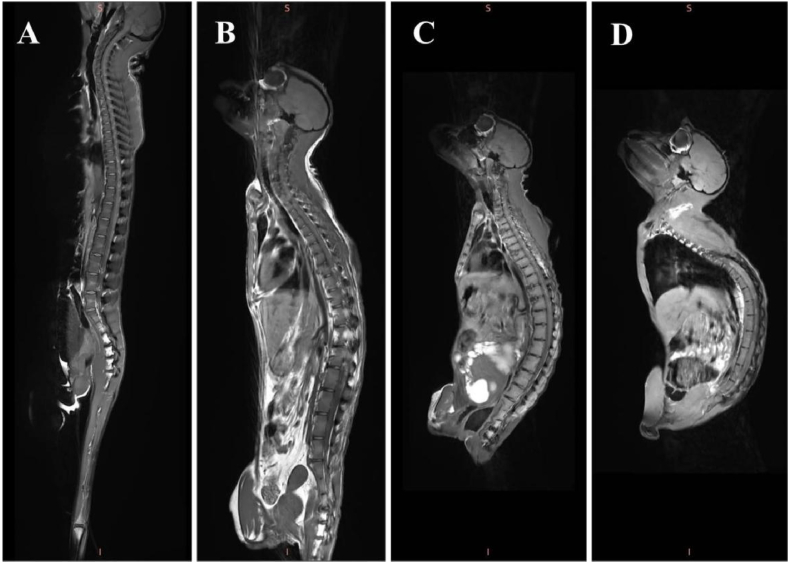
MRI imaging features of spinal arthritis in the crab-eating monkeys. Note: A is ordinary animal, B, C and D are spondyloarthropathy animals.

The MRI imaging examination results reveal the following findings: (1) Spine: Partial vertebral bodies, facet joints and surrounding soft tissues exhibit patchy T2 hyperintense signal changes. (2) Peripheral joints: Some peripheral joints display partial bone defects, narrowing of joint spaces, and T2 hyperintense signal changes in bone and surrounding soft tissues. Overall, inflammatory changes are observed in the vertebral bodies and facet joints, along with associated bone destruction in peripheral joints (Fig. 3A-3D). These findings suggest the presence of inflammation in the spine and peripheral joints。

The comprehensive imaging study results suggest inflammation in the spine and peripheral joints, along with spine osteophyte formation accompanied by partial sclerosis and peripheral joint bone destruction changes, which are similar to the clinical phenotype of human Spondyloarthritis.

### 3.2 Blood biochemical characteristics analysis in spondyloarthritis crab-eating macaque

**Fig. 4:**
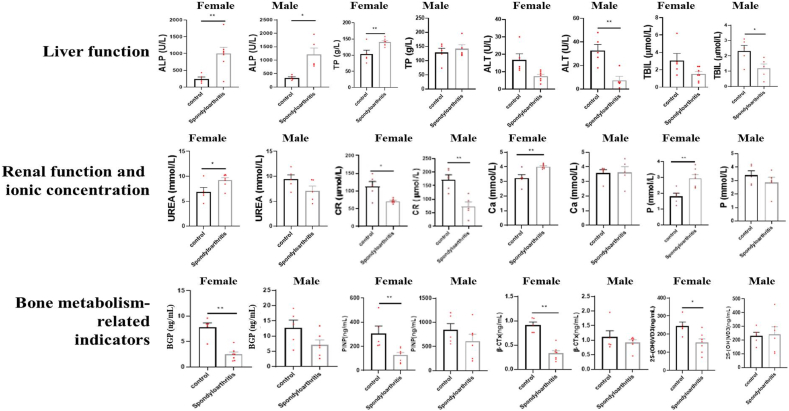
Comparison of average blood biochemical parameters and bone metabolism-related indicators between the diseased group and the control group. Note: Asterisks indicate significant difference (*p* < 0.05).

Comparison of blood biochemical indicators of male and female rhesus monkeys in the diseased group and the control group (Table 3) showed that among the 21 blood biochemical parameters, liver function indicators ALP significantly increased (p < 0.05) and ALT significantly decreased (p < 0.05) in male and female monkeys; renal function indicator CR significantly decreased (p < 0.05); female monkeys showed significant decreases in serum TP, UREA, and LDH (p < 0.05), while LDL-C and ion concentrations (Na, K, Ga, P, etc.) significantly increased (p < 0.05); male monkeys showed significant increases in serum AST and TBIL (p < 0.05) (Table 3, Fig. 4). Significant differences in many biochemical indicators were found in female monkeys, which may be related to their estrous cycle.

**Table 3:** Biochemical parameter values.

Parameters (units)	SpAF		SpAM		CtrlF		CtrlM		*p*-value	
	Mean	Std	Mean	Std	Mean	Std	Mean	Std	SpAF *VS* Ctrl F	SpAM *VS* CtrlM
ALT (U/L)	7.43	2.66	8.25	7.08	16.80	7.03	32.80	10.38	0.0148∗	0.0033∗
AST (U/L)	72.43	21.20	40.00	12.79	59.80	32.89	75.60	23.32	0.4778	0.0267∗
ALP (U/L)	995.14	467.84	1020.75	328.79	244.00	113.08	344.00	77.05	0.0093∗	0.0073∗
GGT (U/L)	95.14	27.94	99.75	57.06	78.00	32.28	80.80	15.30	0.3913	0.4357
TP (g/L)	140.20	10.56	141.45	31.83	102.80	25.15	128.68	30.06	0.0092∗	0.5358
ALB (g/L)	53.57	5.59	50.13	16.80	57.30	11.82	70.24	14.92	0.5212	0.0782
TBIL (μmol/L)	1.53	0.70	1.15	0.63	3.06	1.65	2.32	0.72	0.0736	0.0376∗
UREA (mmol/L)	9.21	1.24	7.50	1.99	6.86	1.81	9.40	1.73	0.0349∗	0.1140
CR (μmol/L)	71.29	5.44	76.50	36.99	112.40	28.54	172.40	36.30	0.0068∗	0.0039∗
GLU (mmol/L)	7.14	1.96	8.58	4.46	5.68	1.35	13.65	3.53	0.2182	0.0800
TG (mmol/L)	1.08	0.23	0.86	0.30	1.17	0.24	1.79	0.75	0.5353	0.0549
TCHO (mmol/L)	4.53	1.45	3.30	1.26	5.37	1.52	3.25	0.58	0.3958	0.9207
HDL-C (mmol/L)	2.20	0.83	1.53	0.76	2.18	1.02	1.51	0.36	0.9761	0.9363
LDL-C (mmol/L)	1.65	0.57	1.13	0.69	2.55	0.67	1.16	0.28	0.0453∗	0.8304
CK (U/L)	318.00	159.29	221.50	105.29	365.80	176.27	368.20	137.50	0.6641	0.1299
LDH (U/L)	1276.14	529.66	591.25	251.46	600.40	221.32	648.60	241.13	0.0339∗	0.7535
Ca (mmol/L)	4.02	0.14	3.68	0.84	3.22	0.48	3.58	0.46	0.0037∗	0.9061
P (mmol/L)	2.94	0.55	2.86	0.86	1.79	0.42	3.40	0.65	0.0048∗	0.3178
Na (mmol/L)	262.63	16.88	228.73	57.36	213.06	33.38	229.96	25.15	0.0117∗	0.8645
K (mmol/L)	8.31	0.42	7.53	2.10	5.44	0.94	6.22	0.50	0.0001∗	0.3205
Cl (mmol/L)	206.76	15.56	173.53	48.00	161.28	28.57	160.38	15.07	0.0090∗	0.7001
BAP(μg/L)	21.30	9.14	43.40	26.14	20.09	7.48	41.15	21.17	0.8285	0.8920
BGP(ng/mL)	2.47	1.16	7.13	3.64	7.82	1.73	12.74	4.99	0.0002∗	0.0834
PINP (ng/mL)	127.47	53.63	608.62	328.65	308.64	119.96	748.73	225.71	0.0091∗	0.5246
PTH (pmol/L)	1.15	1.48	1.28	1.10	1.11	0.44	2.05	0.64	0.9499	0.2436
25-(OH)VD3(ng/mL)	153.40	51.81	243.02	121.10	244.50	44.59	229.80	54.33	0.0158∗	0.8427
β-CTx (ng/mL)	0.33	0.13	0.92	0.22	0.91	0.13	1.11	0.44	0.0001∗	0.4247

Note: BAP (bone alkaline phosphatase), BGP (bone gla protein), PINP (Total type I collagen amino terminal lengthening peptide), PTH (parathyroid hormone), 25-(OH)VD3 (25-Hydroxyvitamin D), β-CTx (β-Crosslaps). Asterisks indicate significant difference (*p* < 0.05).

Analysis of bone metabolism-related indicators showed that compared to the control group, female crab-eating monkeys in the diseased group had decreasing trends in BGP, PINP, 25-(OH)VD3, and β-CTx (p < 0.05), while male monkeys also showed similar trends but did not reach significant differences (Table 3, Fig. 4).

Typical biochemical characteristics of spontaneous spinal arthritis in crab-eating monkeys include significantly elevated alkaline phosphatase levels. Other indicators, such as abnormal liver and kidney function, may be related to the abnormal eating habits caused by spinal curvature, as evidenced by decreasing trends in bone metabolism-related indicators, especially significant decreases in bone formation and other bone metabolism-related indicators.

### 3.3 Transcriptome sequencing analysis

This study employed DNBSEQ for transcriptome sequencing of 23 samples, yielding an average output of 6.69G data per sample. The Q20 of clean reads for each sample exceeded 98 % (Supplementary Table 1). Clean reads were successfully aligned to the reference genome sequence using HISAT, achieving an overall alignment rate of over 97 % and ensuring good transcriptome coverage (Supplementary Table 2). The sequencing data demonstrated overall good quality.

**Fig. 5:**
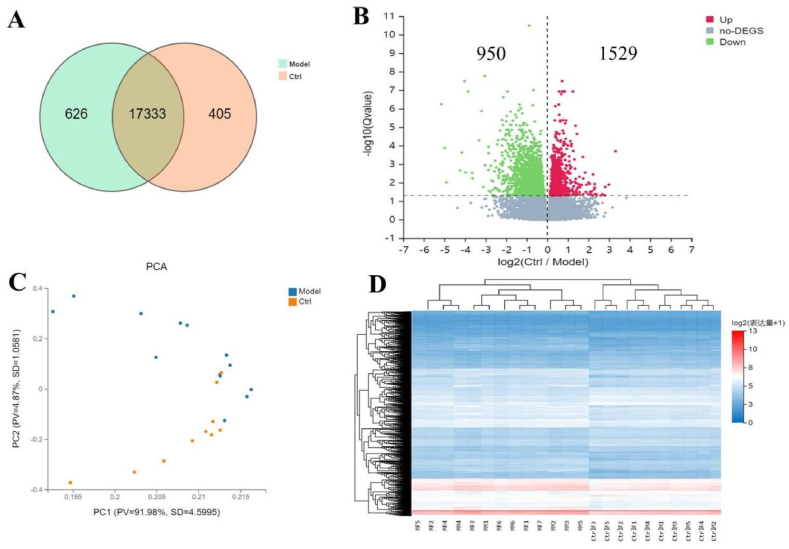
Identification and clustering analysis of differentially expressed genes in transcriptome data. Note: A is the VENN analysis of gene expression between the arthritis group and the control group, B is the volcano plot of differentially expressed genes, C is the PCA analysis, and D is the clustered heat map.

Identification and annotation of differentially expressed genes revealed 17,333 common expressed genes in the spondyloarthritis group and the control group, with unique expressed genes of 626 and 405 respectively (Fig. 5A). Among them, there were 1529 up-regulated genes and 950 down-regulated genes (Fig. 5B). Principal component clustering of differentially expressed genes (Fig. 5C) showed that apart from a few samples showing overlap, the spondyloarthritis group and control group clustered separately. Heatmap analysis (Fig. 5D) indicated significant differences in local gene expression between the spondyloarthritis group and the control group.

**Fig. 6:**
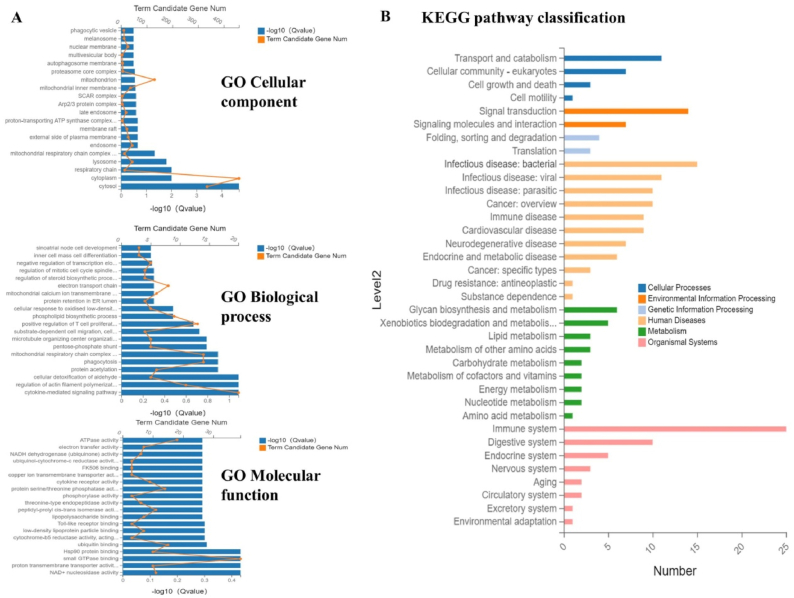
Gene Ontology (GO) and KEGG pathway enrichment analysis. Note: A is GO analysis, B is KEGG analysis.

GO analysis of differentially expressed genes (Fig. 6A) revealed the top four significantly enriched terms in Cellular Component as "cytosol," "cytoplasm," "respiratory chain," and "lysosome." Molecular function showed enrichment in "NAD + nucleosidase activity," "proton transmembrane transporter activity," "small GTPase binding," and "Hsp90 protein binding." The top four significantly enriched terms in Biological process were "cytokine-mediated signaling pathway," "regulation of actin filament polymerization," "cellular detoxification of aldehyde," and "protein acetylation," including one immune-related term "positive regulation of T cell proliferation."

Kegg pathway classification analysis of differentially expressed genes (Fig. 6B) revealed that the most significantly enriched pathways in the Humandisease category are infection, and immune-related disease pathways.

### 3.4 Proteome sequencing analysis

**Fig. 7:**
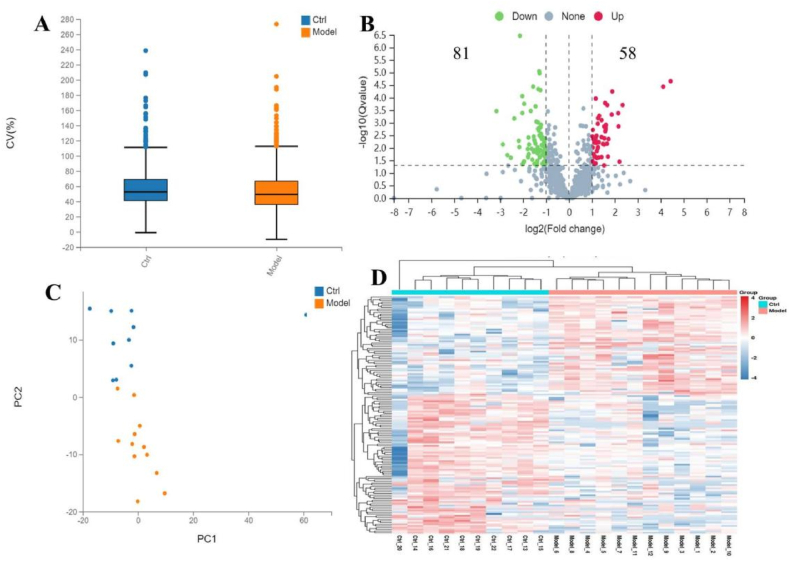
Identification and clustering analysis of differentially expressed proteins in proteome data.

Using label-free quantitative proteomic techniques to analyze control group and arthritis group samples, it was found that 80–90 % of protein CV values were less than 30 % (Fig. 7A), indicating good repeatability. Principal component analysis (PCA) was performed on the samples from both the control group and arthritis group, and it was observed that samples from the same group generally clustered together (Fig. 7C). The sequencing data demonstrated overall good quality.

Analyzing differentially expressed proteins, there were a total of 139 differentially expressed proteins between the arthritis group and the control group, with 58 up-regulated proteins and 81 down-regulated proteins (Fig. 7B). Cluster analysis of differentially expressed proteins between the arthritis group and the control group revealed a significant difference in protein expression patterns between the two groups (Fig. 7D).

**Fig. 8:**
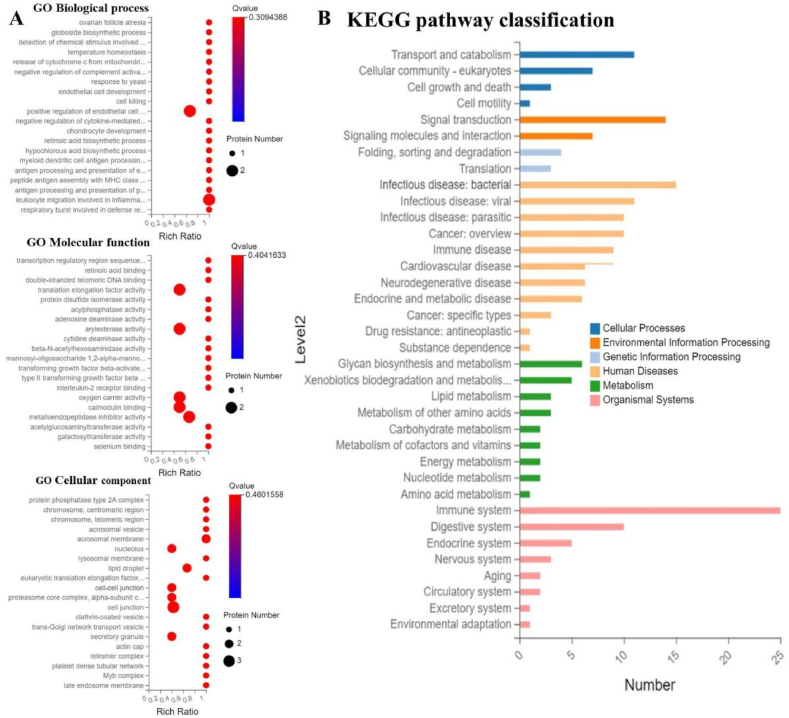
Gene Ontology (GO) and KEGG Pathway Enrichment Analysis in proteome data. Note: A is GO analysis, B is KEGG analysis.

GO analysis of differentially expressed proteins revealed that the top four significantly enriched terms in the Cellular Component category were "cell junction," "nucleolus," "lipid droplet," and "cell-cell junction" (Fig. 8A). In the Molecular Function category, the top four significantly enriched terms were "oxygen carrier activity," "calmodulin binding," "translation elongation factor activity," and "arylesterase activity." In the Biological Process category, the top two significantly enriched terms were "positive regulation of endothelial cell" and "leukocyte migration involved in inflammation," which included an immune-related term "positive regulation of T cell proliferation".

Kegg pathway classification analysis of differentially expressed genes (Fig. 8B) showed that the most significantly enriched pathways in the Humandisease category were infection and immune-related disease pathways. The KEGG analysis results of the proteome were generally consistent with those of the transcriptome, both indicating pathways related to infection or immunity.

Note: A is the analysis of inter-group coefficient of variation (CV), B is the volcano plot of differentially expressed proteins, C is the inter-group principal component analysis (PCA), D is the clustered heat map.

### 3.5 Transcriptome and proteome correlation analysis identifies numerous commonly shared proteins associated with inflammations

**Fig. 9:**
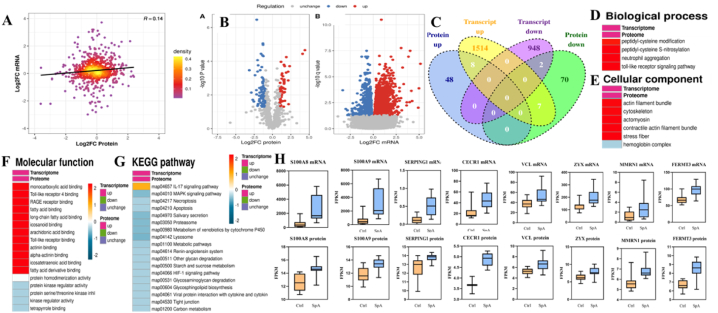
Correlation analysis between the transcriptome and proteome. Note: A represents the correlation of data between the transcriptome and proteome, B is the volcano plot of differential expression between the transcriptome and proteome, C illustrates the co-expression analysis of both the transcriptome and proteome, D to F are the GO analyses for co-expressed highly expressed proteins, G is the KEGG analysis for co-expressed highly expressed proteins, and H is the box plot of expression levels for the eight co-expressed genes and proteins.

Correlation analysis was performed on the transcriptome and proteome expression data, revealing a positive correlation between the transcriptome and proteome expression data (Fig. 9A and B). A total of eight genes and proteins were found to be highly expressed in both the transcriptome and proteome (Table 4) (Fig. 9C). GO (Fig. 9D to F) and KEGG analyses were conducted on these co-expressed genes and proteins. In the GO analysis, significant enrichment was observed in the Biological Process category for inflammation-related pathways (Fig. 9D), including "Neutrophil aggregation" and "toll-like receptor signaling pathway." In the Molecular Function category, the significantly enriched pathways were also predominantly related to inflammation (Fig. 9F), encompassing "Toll-like receptor 4 binding," "RAGE receptor binding," "Arachidonic acid binding," and "Toll-like receptor binding," among others. The KEGG analysis indicated that the co-expressed highly expressed proteins were significantly enriched in the IL-17 signaling pathway (Fig. 9G).

**Table 4:** Transcriptome and proteome share differentially expressed genes and proteins.

Name	Fold change (transcriptome)	Fold change (proteome)
S100A8	4.95	4.43
S100A9	4.77	3.03
SERPING1	4.05	2.21
MMRN1	2.32	2.22
CECR1	2.01	2.30
ZYX	1.54	3.16
VCL	1.44	2.68
FERMT3	1.41	3.71

Note: Asterisks indicate significant difference (p < 0.05).

Analysis of the expression levels of the co-expressed highly expressed genes and proteins was conducted (Fig. 9H), with all eight co-expressed proteins showing an expression level more than twofold higher (Table 4). Among these, S100A8 exhibited the highest expression levels, both in the transcriptome and proteome; S100A9 had the second-highest expression in the transcriptome and the third-highest in the proteome. Additionally, SERPING1, MMRN1, and CECR1 were found to have relatively high expression levels in both the transcriptome and proteome. Out of the eight co-expressed highly expressed proteins, S100A8, S100A9, SERPING1, CECR1, and FERMT3 are all associated with inflammation, with S100A8 and S100A9 being clinically confirmed to be closely related to spondyloarthritis.

### 3.6 WGCNA analysis of clinical and proteomic data has robustly linked S100A8 and S100A9 to the spondyloarthritis phenotype

**Fig. 10:**
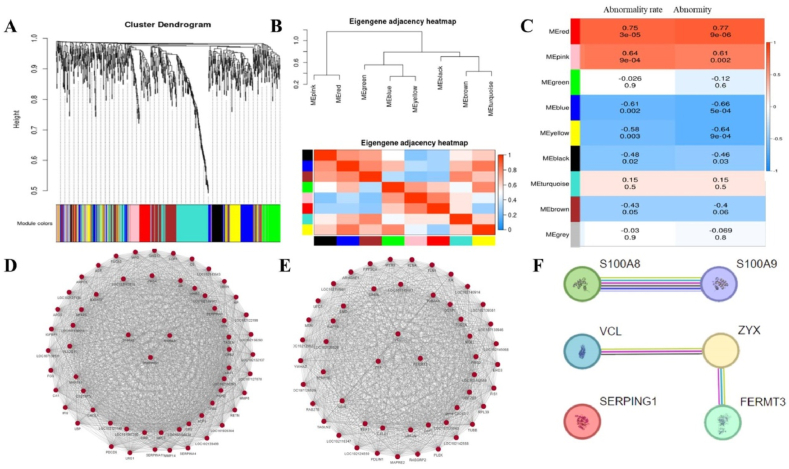
Wgcna analysis of clinical phenotypes of spondyloarthritis in relation to proteomic data. Note: A represents the hierarchical clustering and dynamic tree cutting method that divides all proteins into nine modules, B is the heatmap of inter-module correlations, where red indicates high correlation and blue indicates low correlation, C is the heatmap of correlations between clinical phenotypes of spondyloarthritis and each module, where red represents positive correlation and blue represents negative correlation. The numbers within the grid represent the correlation coefficients and significance p-values between the modules and clinical traits, with the values in parentheses indicating the significance p-values of the correlations.S100A8 and S100A9 may be markers of spontaneous spondyloarthritis in crab-eating macaques.

WGCNA analysis of two clinical datasets, the spinal abnormality rate and the condition of spinal abnormalities (Table 2), in conjunction with proteomic data, categorized all proteins into nine distinct clusters after hierarchical clustering (Fig. 10A). Correlation analysis among these clusters revealed a strong association between the Red and Pink clusters, which are grouped separately (Fig. 10B). The proteins belonging to each cluster are listed in the supplementary file (Supplementary Information Table 3). Association analysis between the proteins in the nine clusters and the two clinical datasets showed that the proteins in the Red and Pink clusters are significantly and positively correlated with spinal abnormalities in spondyloarthritis (Fig. 10C). Analysis of the co-expressed genes and proteins in the transcriptome and proteome revealed that six of the eight highly expressed proteins common to both are identifiable in the significantly correlated Red and Pink clusters, with S100A8, S100A9, and SERPING1 in the Red cluster, and ZYZ, VCL, and FERMT3 in the Pink cluster (Fig. 10D and E). It is evident that the high expression of S100A8, S100A9, SERPING1, ZYZ, VCL, and FERMT3 is positively correlated with spondyloarthritis. Interaction analysis of S100A8, S100A9, SERPING1, ZYZ, VCL, and FERMT3 (Fig. 10F) showed significant interactions between S100A8 and S100A9, and among ZYX, VCL, and FERMT3. This further confirms the close relationship of S100A8 and S100A9 with the phenotype of this spondyloarthritis.

### 3.7 S100A8 and S100A9 may be markers of spontaneous spondyloarthritis in crab-eating macaques

S100A8 and S100A9 are highly expressed in both the transcriptome and proteome (Fig. 9H), with S100A8 being the most highly expressed among the shared proteins. Both are significantly enriched in the IL-17 signaling pathway (Fig. 9G), and their high expression is significantly positively correlated with the clinical phenotype of spondyloarthritis (Fig. 10C). S100A8 and S100A9 may be markers of spontaneous spondyloarthritis in crab-eating macaques.

## 4 Discussion

### 4.1 The pathological and physiological characteristics of spontaneous spondyloarthritis in crab-eating macaques closely resemble those found in human clinical cases

Analysis of X-ray and MRI imaging of spontaneous spondyloarthropathy in crab-eating macaques revealed significant findings. X-ray imaging displayed the presence of spinal osteophytes and destruction of peripheral joint bones, while MRI showed inflammation changes, in the vertebral bodies and facet joints (Table 4, Fig. 3). Peripheral joint inflammation was also observed, accompanied by bone destruction. Moreover, markers for both bone formation and resorption were found to be decreased (Fig. 4). The bone and joint characteristics of spontaneous spondyloarthropathy in crab-eating macaques closely resembled the clinical phenotype seen in humans [[20], [21], [22], [23]]. Blood biochemistry analysis further revealed abnormalities in liver function-related indicators and 1 kidney function indicator in the macaques (Fig. 4). Studies in humans have shown that long-term spondyloarthropathy can lead to liver function abnormalities [24,25]. The abnormal liver function observed in spondyloarthropathy may be attributed to various factors, including chronic inflammation in the body inducing peripheral vascular inflammation and, subsequently, liver function abnormalities. Long-term restricted activity may also contribute to liver function abnormalities. Overall, it is evident that the imaging and biochemical characteristics of spontaneous spondyloarthropathy in crab-eating macaques closely resemble the clinical phenotype seen in humans.

### 4.2 Spondyloarthritis triggers the body to generate inflammatory responses in crab-eating macaques

Differential gene and protein analysis of transcriptome and proteome revealed a significant number of immune and infection-related disease pathways, including "Immune system," "Endocrine system," "Immune disease," and "Infectious disease," with high consistency between transcriptome and proteome in these pathways. Analysis of differentially expressed genes and proteins in transcriptome and proteome identified 17 shared proteins, and enrichment analysis of these shared proteins revealed 4 inflammation-related pathways among the top 6 enriched pathways, including "Neutrophil aggregation," "Autocrine signaling," "Chronic inflammatory response," and "Leukocyte cell-cell adhesion." The top 2 enriched Molecular Functions were both related to inflammation, namely "Arachidonic acid binding" and "Toll-like receptor 4 binding." Furthermore, analysis of the 17 shared proteins revealed that the proteins S100A8 and S100A9, which exhibited the highest expression levels in the SpA group, are closely related to arthritis, while FERMT3 is crucial for maintaining leukocyte function [26]. It is evident that spontaneous spondyloarthritis in the crab-eating monkey results in inflammation in the body, causing a significant increase in inflammation protein markers linked to arthritis. Additionally, while the HLA-B27 marker associated with ankylosing spondylitis did not demonstrate abnormal expression in the monkey, the expression of C-reactive protein (CRP) in the proteome showed a notable increase (4.43-fold). These findings suggest that spontaneous spondyloarthritis in the crab-eating monkey stimulates an inflammatory response.

### 4.3 Spontaneous spondyloarthritis in the crab-eating macaque is closely associated with the autoimmune disease biomarkers S100A8 and S100A9

Analysis of key common proteins has revealed that the proteins with the highest expression in the spondyloarthritis group are S100A8 and S100A9. Studies have shown that S100A8 and S100A9 play crucial roles in various pathological processes of chronic inflammatory diseases. These proteins primarily mediate intracellular inflammatory signal transduction by binding to and activating Toll-like receptors and advanced glycation end product receptors [27]. Research has indicated that S100A8 and S100A9 are highly expressed in autoimmune diseases such as Rheumatoid Arthritis (RA), Systemic Lupus Erythematosus (SLE), Psoriasis (Pa) and Juvenile Idiopathic Arthritis (SoJIA). In active RA patients, S100A8 and S100A9 are significantly upregulated, particularly in individuals with bone destruction or synovium [28,29]. Macrophages release S100A8 and S100A9 dimers extracellularly upon activation, leading to the amplification of inflammatory factors and subsequent bone damage [30]. These proteins are also potential markers for predicting and evaluating the efficacy of clinical treatment for RA [31,32]. In SLE patients, the expression of S100A8 and S100A9 on the surface of dendritic cells is notably increased [33], especially during periods of infection [34]. Similarly, in Pa patients, S100A8 and S100A9 are expressed in skin lesions and present in elevated levels in the serum [35,36], and these proteins are also considered potential markers for psoriatic arthritis [37]. In patients with SSc, the levels of S100A8 and S100A9 are found to be elevated in their saliva, with significantly higher expression in their serum compared to healthy individuals [38,39]. Similarly, in patients with SoJIA, the serum levels of S100A8 and S100A9 are notably elevated, with a 12-fold increase in active polyarticular JIA patients and a striking 120-fold increase compared to normal controls [40]. This suggests a strong association between the presence of autoimmune disease markers S100A8 and S100A9 and the development of spontaneous ankylosing spondylitis in crab-eating monkeys.

## 5 Conclusion

In order to explore the pathophysiological characteristics of spontaneous non-human primate spondyloarthritis, this study systematically investigated the disease features in the affected animals using various methods such as X-ray imaging, MRI imaging, blood biochemistry, bone metabolism markers, transcriptomics, proteomics, and metabolomics. The X-ray imaging results showed that the affected monkeys with spondyloarthritis exhibited bone spurs formation in the spine and peripheral joint bone destruction. MRI imaging revealed inflammation changes in the vertebral bodies and facet joints of the affected monkeys, along with peripheral joint inflammation and bone destruction. Blood biochemistry analysis indicated abnormalities in liver function and kidney function indexes in the affected monkeys. Analysis of bone metabolism markers showed a decrease in bone formation (BGP) and bone resorption (β-CTx). A thorough correlation analysis was conducted on the transcriptome and proteome expression data, revealing a significant positive correlation between the two datasets. A total of eight genes and proteins with high expression levels were identified as common to both the transcriptome and proteome. Subsequent Gene Ontology (GO) and Kyoto Encyclopedia of Genes and Genomes (KEGG) analyses were performed on these co-expressed genes and proteins, indicating a predominant enrichment in the IL-17 signaling pathway, with S100A8 and S100A9 identified as the core proteins. Further analysis using clinical data in conjunction with proteome data through Weighted Gene Co-expression Network Analysis (WGCNA) demonstrated a significant positive correlation between the high expression of S100A8 and S100A9 and the clinical phenotypes of spinal abnormalities, thereby corroborating the close association of S100A8 and S100A9 with the phenotype of spondyloarthritis. Human clinical studies have already established a link between S100A8 and S100A9 and autoimmune-related arthritic diseases, suggesting that the spontaneous spondyloarthritis observed in crab-eating macaques may be related to autoimmune diseases. It is hypothesized that S100A8 and S100A9 could serve as potential predictive biomarkers for this spondyloarthritis in non-human primates.

## CRediT authorship contribution statement

**Lei Cai:** Writing – original draft. **Qing Lv:** Validation, Methodology. **Ronghua Ma:** Methodology. **Wei Liu:** Methodology. **Yalun Guan:** Methodology, Data curation. **Zhongqiang Huang:** Methodology, Data curation. **Qingyu Liu:** Validation, Methodology. **Yunfeng Li:** Methodology. **Shuhua Liu:** Methodology. **Ge Li:** Supervision, Methodology, Data curation, Conceptualization. **Yu Zhang:** Writing – review & editing, Validation, Supervision, Conceptualization.

## Data availability

RNA-Seq sequencing reads have been deposited in the NCBI Sequence Read Archive under project PRJNA1109446. The mass spectrometry proteomics data have been deposited to the iProX with the dataset identifer PXD052158.

## Declaration of Competing Interest

The authors declare that they have no known competing financial interests or personal relationships that could have appeared to influence the work reported in this paper.
