# Supplementary material for: Characteristics and multi-omics analysis of spontaneous spondyloarthritis in non-human primates: Case report
Source: Heliyon. 2025 Jan 8;11(2):e41706. doi: 10.1016/j.heliyon.2025.e41706 (PMC11773035; doi:10.1016/j.heliyon.2025.e41706)
Supplement: Multimedia component 1 [file mmc1.docx]

Supplementary Material

**Characteristics and Multi-omics Analysis of Spontaneous Spondyloarthritis in Non-Human Primates: Case Report**

**Supplementary data**

**Supplementary table 1 Transcriptome sequencing results**

| **Sample** | **Total Raw Reads (M)** | **Total Clean Reads (M)** | **Total Clean Bases (Gb)** | **Clean Reads Q20 (%)** | **Clean Reads Q30 (%)** | **Clean Reads Ratio (%)** |
| --- | --- | --- | --- | --- | --- | --- |
| CtrlF1 | 45.44 | 44.68 | 6.7 | 98.43 | 93.87 | 98.33 |
| CtrlF2 | 45.44 | 44.52 | 6.68 | 98.39 | 93.71 | 97.98 |
| CtrlF3 | 45.44 | 44.43 | 6.66 | 98.49 | 94.13 | 97.78 |
| CtrlF4 | 45.44 | 44.51 | 6.68 | 98.44 | 93.88 | 97.95 |
| CtrlF5 | 45.44 | 44.63 | 6.69 | 98.36 | 93.61 | 98.22 |
| CtrlM1 | 45.44 | 44.52 | 6.68 | 98.42 | 93.88 | 97.98 |
| CtrlM2 | 47.19 | 44.34 | 6.65 | 98.74 | 95 | 93.96 |
| CtrlM3 | 45.44 | 44.76 | 6.71 | 98.4 | 93.69 | 98.5 |
| CtrlM4 | 45.44 | 44.73 | 6.71 | 98.47 | 94 | 98.44 |
| CtrlM5 | 45.44 | 44.07 | 6.61 | 98.44 | 94.01 | 96.99 |
| MF1 | 45.44 | 44.85 | 6.73 | 98.35 | 93.61 | 98.7 |
| MF2 | 45.44 | 44.86 | 6.73 | 98.16 | 92.7 | 98.72 |
| MF3 | 47.19 | 44.99 | 6.75 | 98.49 | 94.18 | 95.34 |
| MF4 | 45.44 | 44.59 | 6.69 | 98.41 | 93.88 | 98.13 |
| MF5 | 45.44 | 44.41 | 6.66 | 98.26 | 93.25 | 97.73 |
| MF6 | 45.44 | 44.84 | 6.73 | 98.34 | 93.47 | 98.68 |
| MF7 | 45.44 | 44.73 | 6.71 | 98.34 | 93.52 | 98.44 |
| MM1 | 45.44 | 44.69 | 6.7 | 98.5 | 94.12 | 98.35 |
| MM2 | 45.44 | 43.97 | 6.6 | 98.55 | 94.32 | 96.76 |
| MM3 | 45.44 | 44.84 | 6.73 | 98.34 | 93.53 | 98.68 |
| MM4 | 45.44 | 44.5 | 6.68 | 98.5 | 94.2 | 97.93 |
| MM5 | 45.44 | 44.82 | 6.72 | 98.39 | 93.77 | 98.64 |
| MM6 | 45.44 | 44.6 | 6.69 | 98.41 | 93.84 | 98.15 |

**Supplementary Table 2 Aligning with the reference genome**

| **Sample** | **Total Clean Reads (M)** | **Total Mapping(%)** | **Uniquely Mapping(%)** |
| --- | --- | --- | --- |
| CtrlF1 | 44.68 | 97.79 | 95.09 |
| CtrlF2 | 44.52 | 97.78 | 95.12 |
| CtrlF3 | 44.43 | 97.82 | 95.63 |
| CtrlF4 | 44.51 | 97.85 | 94.72 |
| CtrlF5 | 44.63 | 97.81 | 95.78 |
| CtrlM1 | 44.52 | 97.57 | 95.28 |
| CtrlM2 | 44.34 | 97.14 | 94 |
| CtrlM3 | 44.76 | 97.83 | 94.07 |
| CtrlM4 | 44.73 | 97.7 | 95.63 |
| CtrlM5 | 44.07 | 97.42 | 94.18 |
| MF1 | 44.85 | 97.95 | 95.55 |
| MF2 | 44.86 | 97.98 | 95.55 |
| MF3 | 44.99 | 97.74 | 94.23 |
| MF4 | 44.59 | 97.81 | 95.49 |
| MF5 | 44.41 | 97.72 | 94.72 |
| MF6 | 44.84 | 98.05 | 93.62 |
| MF7 | 44.73 | 97.88 | 95.61 |
| MM1 | 44.69 | 97.72 | 95.49 |
| MM2 | 43.97 | 97.46 | 93.31 |
| MM3 | 44.84 | 97.82 | 95.55 |
| MM4 | 44.5 | 97.67 | 95.48 |
| MM5 | 44.82 | 97.79 | 95.54 |
| MM6 | 44.6 | 97.75 | 95.69 |

**Supplementary Table 3 All the proteins contained within the red block**

| Protein accession | Protein description | Gene name | Module | Abnormality_rate | Vertebral_abnomaly |
| --- | --- | --- | --- | --- | --- |
| A0A2K5VVZ6 | Pentaxin OS=Macaca fascicularis OX=9541 GN=EGM_01251 PE=3 SV=1 | CRP | red | 0.748868 | 0.76427 |
| G7PIV0 | Activin beta-C chain OS=Macaca fascicularis OX=9541 GN=EGM_03411 PE=3 SV=1 | INHBC | red | 0.73198 | 0.698368 |
| A0A2K5VN00 | Serum amyloid A protein OS=Macaca fascicularis OX=9541 PE=3 SV=2 | LOC102138293 | red | 0.727376 | 0.699881 |
| G7PGE5 | Lipopolysaccharide-binding protein OS=Macaca fascicularis OX=9541 GN=LBP PE=3 SV=2 | LBP | red | 0.725787 | 0.726463 |
| A0A2K5V406 | Matrix metallopeptidase 8 OS=Macaca fascicularis OX=9541 GN=MMP8 PE=3 SV=2 | MMP8 | red | 0.71236 | 0.662657 |
| A0A2K5X012 | Peptidase_M14 domain-containing protein OS=Macaca fascicularis OX=9541 PE=3 SV=2 | CPB2 | red | 0.709122 | 0.729722 |
| A0A2K5UK15 | Serum amyloid A protein OS=Macaca fascicularis OX=9541 PE=3 SV=2 | LOC102138293 | red | 0.694673 | 0.717713 |
| A0A2K5VRF5 | Proteoglycan 4 OS=Macaca fascicularis OX=9541 GN=PRG4 PE=4 SV=2 | PRG4 | red | 0.690295 | 0.729773 |
| A0A2K5WX40 | alpha-1,2-Mannosidase OS=Macaca fascicularis OX=9541 GN=MAN1A1 PE=3 SV=2 | MAN1A1 | red | 0.674856 | 0.703801 |
| A0A2K5V5Z0 | Hexosyltransferase OS=Macaca fascicularis OX=9541 GN=B3GNT7 PE=3 SV=1 | B3GNT7 | red | 0.66898 | 0.667149 |
| G7P9U2 | Matrix metalloproteinase-14 OS=Macaca fascicularis OX=9541 GN=MMP14 PE=3 SV=1 | MMP14 | red | 0.656876 | 0.597777 |
| A0A7N9DA00 | Pentaxin OS=Macaca fascicularis OX=9541 GN=APCS PE=3 SV=1 >tr\|G8F650\|G8F650_MACFA Pentaxin OS=Macaca fascicularis OX=9541 GN=EGM_21230 PE=3 SV=1 | APCS | red | 0.647862 | 0.698342 |
| A0A2K5X560 | Protein S100 OS=Macaca fascicularis OX=9541 GN=S100A8 PE=3 SV=1 | S100A8 | red | 0.637454 | 0.682967 |
| A0A2K5U1T1 | Complement C5 OS=Macaca fascicularis OX=9541 GN=C5 PE=4 SV=2 | C5 | red | 0.620498 | 0.656676 |
| A0A2K5VSD9 | Serpin family A member 3 OS=Macaca fascicularis OX=9541 GN=SERPINA3 PE=3 SV=2 | SERPINA3 | red | 0.62043 | 0.635945 |
| G8F2E1 | Angiotensinogen OS=Macaca fascicularis OX=9541 GN=EGM_20106 PE=3 SV=1 | AGT | red | 0.614107 | 0.63359 |
| A0A7N9C893 | Immunoglobulin kappa variable 4-1 OS=Macaca fascicularis OX=9541 GN=IGKV4-1 PE=4 SV=1 | LOC107127070 | red | 0.609393 | 0.629635 |
| A0A2K5VTE5 | SERPIN domain-containing protein OS=Macaca fascicularis OX=9541 GN=EGM_16913 PE=3 SV=1 | SERPINA11 | red | 0.601264 | 0.6558 |
| A0A2K5VRV3 | Protein S100 OS=Macaca fascicularis OX=9541 GN=S100A9 PE=3 SV=1 | S100A9 | red | 0.596468 | 0.621795 |
| G7PYX4 | Cysteine-rich secreted protein FIZZ3 OS=Macaca fascicularis OX=9541 GN=EGM_09188 PE=3 SV=1 | RETN | red | 0.586137 | 0.551862 |
| G7NVF9 | Chitinase-3-like protein 1 OS=Macaca fascicularis OX=9541 GN=EGM_01508 PE=3 SV=1 | CHI3L1 | red | 0.570113 | 0.645817 |
| A0A2K5UPZ9 | Myeloperoxidase OS=Macaca fascicularis OX=9541 GN=MPO PE=4 SV=2 | MPO | red | 0.55597 | 0.554236 |
| A0A2K5VVA4 | Leucine rich alpha-2-glycoprotein 1 OS=Macaca fascicularis OX=9541 GN=LRG1 PE=4 SV=2 | LRG1 | red | 0.551888 | 0.566741 |
| Q9BE24 | L-lactate dehydrogenase A chain OS=Macaca fascicularis OX=9541 GN=LDHA PE=2 SV=4 | LOC101867330 | red | 0.541084 | 0.527743 |
| A0A2K5TSV9 | Serpin family G member 1 OS=Macaca fascicularis OX=9541 GN=SERPING1 PE=3 SV=2 | SERPING1 | red | 0.522894 | 0.548115 |
| A0A2K5UHF8 | Serpin family A member 4 OS=Macaca fascicularis OX=9541 GN=SERPINA4 PE=3 SV=2 | SERPINA4 | red | 0.52243 | 0.56351 |
| A0A2K5VNV8 | Alpha-L-fucosidase OS=Macaca fascicularis OX=9541 GN=FUCA2 PE=3 SV=2 | FUCA2 | red | 0.517982 | 0.565161 |
| A0A2K5WKT2 | Glutaminyl-peptide cyclotransferase OS=Macaca fascicularis OX=9541 GN=QPCT PE=3 SV=2 | QPCT | red | 0.514708 | 0.51768 |
| G7PR44 | Lipocln_cytosolic_FA-bd_dom domain-containing protein (Fragment) OS=Macaca fascicularis OX=9541 GN=EGM_06489 PE=4 SV=1 | C8G | red | 0.512298 | 0.51644 |
| A0A2K5UFR8 | RNAse_Pc domain-containing protein OS=Macaca fascicularis OX=9541 PE=3 SV=2 | LOC102122199 | red | 0.473428 | 0.455766 |
| A0A7N9CJX5 | C1q and TNF related 5 OS=Macaca fascicularis OX=9541 PE=4 SV=1 | C1QTNF5 | red | 0.466116 | 0.400694 |
| A0A2K5X489 | Microfibril associated protein 5 OS=Macaca fascicularis OX=9541 PE=3 SV=1 | MFAP5 | red | 0.454785 | 0.490617 |
| A0A2K5TY19 | Haptoglobin OS=Macaca fascicularis OX=9541 PE=3 SV=2 | HP | red | 0.45324 | 0.453221 |
| I7GNR1 | Sulfotransferase OS=Macaca fascicularis OX=9541 PE=2 SV=1 | LOC101926364 | red | 0.451263 | 0.441783 |
| A0A2K5VFG7 | Guanine nucleotide-binding protein subunit gamma OS=Macaca fascicularis OX=9541 GN=EGM_00739 PE=3 SV=1 | GNG12 | red | 0.449138 | 0.430744 |
| A0A7N9CSF3 | Programmed cell death 6 OS=Macaca fascicularis OX=9541 PE=4 SV=1 | PDCD6 | red | 0.435017 | 0.533279 |
| A0A2K5WHS2 | Interferon alpha inducible protein 6 OS=Macaca fascicularis OX=9541 GN=IFI6 PE=3 SV=1 | IFI6 | red | 0.416409 | 0.477554 |
| A0A2K5X057 | Phospholipase A2 group XV OS=Macaca fascicularis OX=9541 GN=PLA2G15 PE=3 SV=1 | PLA2G15 | red | 0.41492 | 0.435244 |
| A0A2K5ULY6 | Ig-like domain-containing protein OS=Macaca fascicularis OX=9541 PE=4 SV=2 | LOC102142875 | red | 0.398967 | 0.406226 |
| A0A7N9CZ46 | Immunoglobulin heavy variable 6-1 OS=Macaca fascicularis OX=9541 GN=IGHV6-1 PE=4 SV=1 | LOC107130511 | red | 0.397561 | 0.426827 |
| A0A2K5TK33 | Lymphocyte cytosolic protein 1 OS=Macaca fascicularis OX=9541 GN=LCP1 PE=4 SV=2 | LCP1 | red | 0.387324 | 0.391225 |
| G7PI79 | Uncharacterized protein (Fragment) OS=Macaca fascicularis OX=9541 GN=EGM_03749 PE=3 SV=1 | ARPC3 | red | 0.355084 | 0.369753 |
| A0A2K5W850 | Hexose-6-phosphate dehydrogenase/glucose 1-dehydrogenase OS=Macaca fascicularis OX=9541 GN=H6PD PE=4 SV=1 | H6PD | red | 0.336986 | 0.371721 |
| Q2PG59 | Transgelin OS=Macaca fascicularis OX=9541 GN=EGM_06264 PE=2 SV=1 | TAGLN | red | 0.313416 | 0.258317 |
| A0A2K5X958 | Transferrin receptor protein 1 OS=Macaca fascicularis OX=9541 GN=TFRC PE=3 SV=2 | TFRC | red | 0.308915 | 0.330433 |
| A0A7N9IGN0 | Ig-like domain-containing protein OS=Macaca fascicularis OX=9541 PE=4 SV=1 | LOC102143543 | red | 0.263573 | 0.267523 |
| A0A2K5VSQ4 | Tartrate-resistant acid phosphatase type 5 OS=Macaca fascicularis OX=9541 GN=EGM_09288 PE=4 SV=1 | ACP5 | red | 0.178297 | 0.207048 |
| G8F694 | Ig-like domain-containing protein (Fragment) OS=Macaca fascicularis OX=9541 GN=EGM_19429 PE=4 SV=1 | LOC102139499 | red | 0.162768 | 0.214461 |
| A0A2K5U960 | Cytokeratin-1 OS=Macaca fascicularis OX=9541 GN=KRT1 PE=3 SV=2 | KRT1 | red | 0.160768 | 0.140895 |
| A0A2K5USE2 | Ig-like domain-containing protein OS=Macaca fascicularis OX=9541 PE=4 SV=2 | LOC102127136 | red | 0.099933 | 0.136915 |
| G7P6F9 | Fibrinogen C-terminal domain-containing protein OS=Macaca fascicularis OX=9541 GN=EGM_14763 PE=4 SV=1 | FGG | red | 0.013963 | 0.050784 |
| A0A2K5V790 | Tyrosine-protein kinase OS=Macaca fascicularis OX=9541 GN=SYK PE=3 SV=2 | SYK | red | -0.02617 | -0.13341 |
| A0A2K5UNK0 | Ig-like domain-containing protein OS=Macaca fascicularis OX=9541 PE=4 SV=2 | LOC102138638 | red | -0.10506 | -0.07135 |
| A0A172R2H1 | Rearranged Ig variable region VDJ (Fragment) OS=Macaca fascicularis OX=9541 GN=IGH PE=2 SV=1 | LOC107130514 | red | -0.22447 | -0.28539 |
| A0A7N9DGB0 | Immunoglobulin lambda variable 4-3 OS=Macaca fascicularis OX=9541 GN=IGLV4-3 PE=4 SV=1 | LOC102121140 | red | -0.25849 | -0.31421 |
| G7PC52 | Carbonic anhydrase OS=Macaca fascicularis OX=9541 GN=EGM_17448 PE=3 SV=1 | CA1 | red | -0.2879 | -0.33973 |
| A0A2K5TY12 | Haptoglobin OS=Macaca fascicularis OX=9541 PE=3 SV=2 | HP | red | -0.47367 | -0.484 |
| A0A7N9D1Q1 | Insulin-like growth factor-binding protein 1 OS=Macaca fascicularis OX=9541 GN=IGFBP1 PE=4 SV=1 | IGFBP1 | red | -0.58665 | -0.58204 |
| A0A7N9IB83 | Uncharacterized protein OS=Macaca fascicularis OX=9541 PE=4 SV=1 | LOC102139343 | red | -0.64138 | -0.6503 |
| A0A7N9CPG3 | Uncharacterized protein OS=Macaca fascicularis OX=9541 PE=4 SV=1 | LOC102132137 | red | -0.65828 | -0.71264 |
| A0A7N9I9M5 | Suprabasin OS=Macaca fascicularis OX=9541 GN=SBSN PE=4 SV=1 | SBSN | red | -0.72211 | -0.71262 |

**Supplementary Table 4 All the proteins contained within the pink block**

| Protein accession | Protein description | Gene name | Module | Abnormality_rate | Vertebral_abnomaly |
| --- | --- | --- | --- | --- | --- |
| A0A2K5U8F3 | Fermitin family member 3 OS=Macaca fascicularis OX=9541 GN=FERMT3 PE=3 SV=2 | FERMT3 | pink | 0.736261 | 0.746754 |
| A0A2K5VGA4 | Talin 1 OS=Macaca fascicularis OX=9541 GN=TLN1 PE=4 SV=2 | TLN1 | pink | 0.70927 | 0.70001 |
| A0A2K5W2A6 | Adenylyl cyclase-associated protein OS=Macaca fascicularis OX=9541 PE=3 SV=1 | CAP1 | pink | 0.693873 | 0.609518 |
| A0A2K5V663 | Monoglyceride lipase OS=Macaca fascicularis OX=9541 GN=MGLL PE=4 SV=2 | MGLL | pink | 0.68651 | 0.604267 |
| G7PWU0 | Small monomeric GTPase OS=Macaca fascicularis OX=9541 GN=RAB27B PE=3 SV=1 | RAB27B | pink | 0.681864 | 0.6527 |
| G7Q216 | Uncharacterized protein (Fragment) OS=Macaca fascicularis OX=9541 GN=EGM_19347 PE=3 SV=1 | FLNA | pink | 0.664652 | 0.658587 |
| A0A2K5TL59 | Granulin OS=Macaca fascicularis OX=9541 PE=3 SV=2 | GRN | pink | 0.635293 | 0.617202 |
| A0A2K5UKH0 | Uncharacterized protein OS=Macaca fascicularis OX=9541 PE=4 SV=2 | LOC102142558 | pink | 0.623599 | 0.64213 |
| A0A2K5VSW8 | Emerin OS=Macaca fascicularis OX=9541 GN=EMD PE=4 SV=2 | EMD | pink | 0.622897 | 0.548626 |
| A0A2K5UJW6 | Immunoglobulin lambda variable 3-9 OS=Macaca fascicularis OX=9541 GN=IGLV3-9 PE=4 SV=2 | LOC107131043 | pink | 0.602838 | 0.584021 |
| A0A2K5U1Y6 | C-X-C motif chemokine OS=Macaca fascicularis OX=9541 PE=3 SV=2 | gene-CXCL3-2 | pink | 0.598651 | 0.590007 |
| A0A7N9DFU9 | Metavinculin OS=Macaca fascicularis OX=9541 GN=VCL PE=3 SV=1 | VCL | pink | 0.591415 | 0.543838 |
| A0A2K5V3U7 | Calponin OS=Macaca fascicularis OX=9541 PE=3 SV=2 | LOC102118347 | pink | 0.586056 | 0.469789 |
| A0A023JCR5 | GST class-pi OS=Macaca fascicularis OX=9541 GN=GSTP1 PE=2 SV=1 | GSTP1 | pink | 0.565897 | 0.526933 |
| A0A2K5V8P9 | Ubiquitin-fold modifier-conjugating enzyme 1 OS=Macaca fascicularis OX=9541 GN=UFC1 PE=3 SV=2 | UFC1 | pink | 0.563909 | 0.54327 |
| A0A2K5VMD9 | Tubulin beta chain OS=Macaca fascicularis OX=9541 GN=TUBB PE=3 SV=2 | TUBB | pink | 0.554139 | 0.530156 |
| G8F3Q1 | Ig-like domain-containing protein (Fragment) OS=Macaca fascicularis OX=9541 GN=EGM_20070 PE=4 SV=1 | LOC102145068 | pink | 0.547517 | 0.453767 |
| A0A2K5W160 | Uncharacterized protein (Fragment) OS=Macaca fascicularis OX=9541 GN=EGM_12979 PE=4 SV=1 | CALD1 | pink | 0.545604 | 0.554304 |
| A0A7N9CIC2 | RAS guanyl releasing protein 2 OS=Macaca fascicularis OX=9541 PE=3 SV=1 | RASGRP2 | pink | 0.545358 | 0.463973 |
| A0A2K5VL27 | Zyxin OS=Macaca fascicularis OX=9541 GN=ZYX PE=4 SV=2 | ZYX | pink | 0.542504 | 0.542654 |
| Q4R9D4 | Ras-related protein Rap-1b OS=Macaca fascicularis OX=9541 GN=RAP1B PE=2 SV=1 >tr\|Q25DA2\|Q25DA2_MACFA Brain cDNA clone: QtrA-17332, similar to human RAP1B, member of RAS oncogene family (RAP1B) OS=Macaca fascicularis OX=9541 PE=2 SV=1 | RAP1B | pink | 0.537131 | 0.527918 |
| A0A2K5WC70 | Serine/threonine-protein phosphatase OS=Macaca fascicularis OX=9541 GN=PPP3CA PE=3 SV=2 | PPP3CA | pink | 0.524614 | 0.555051 |
| A0A2K5UPI7 | PDZ and LIM domain 1 OS=Macaca fascicularis OX=9541 GN=PDLIM1 PE=4 SV=2 | PDLIM1 | pink | 0.518859 | 0.467847 |
| A0A2K5W4D6 | Myosin light chain 12A OS=Macaca fascicularis OX=9541 GN=MYL12A PE=4 SV=2 | LOC102124550 | pink | 0.496937 | 0.461831 |
| A0A172R2E9 | Rearranged Ig variable region VDJ (Fragment) OS=Macaca fascicularis OX=9541 GN=IGH PE=2 SV=1 | LOC107126526 | pink | 0.488023 | 0.393719 |
| A0A2K5U150 | Serglycin OS=Macaca fascicularis OX=9541 GN=SRGN PE=4 SV=1 | SRGN | pink | 0.421542 | 0.389458 |
| Q4R4I1 | Ubiquitin-conjugating enzyme E2 N OS=Macaca fascicularis OX=9541 GN=UBE2N PE=2 SV=1 | UBE2N | pink | 0.418878 | 0.348722 |
| G7PCF6 | Tyrosine 3-monooxygenase/tryptophan 5-monooxygenase activation protein zeta OS=Macaca fascicularis OX=9541 GN=YWHAZ PE=3 SV=1 | YWHAZ | pink | 0.418797 | 0.353801 |
| A0A2K5VXJ9 | Bone marrow proteoglycan OS=Macaca fascicularis OX=9541 GN=EGM_05596 PE=4 SV=2 | PRG2 | pink | 0.387541 | 0.303313 |
| P68367 | Tubulin alpha-4A chain OS=Macaca fascicularis OX=9541 GN=TUBA4A PE=2 SV=1 | TUBA4A | pink | 0.385279 | 0.399901 |
| A0A2K5WGD3 | 59 kDa serine/threonine-protein kinase OS=Macaca fascicularis OX=9541 GN=EGM_05913 PE=4 SV=1 | ILK | pink | 0.380263 | 0.314656 |
| A0A2K5UBX7 | Myosin heavy chain 9 OS=Macaca fascicularis OX=9541 GN=MYH9 PE=3 SV=2 | MYH9 | pink | 0.347463 | 0.374346 |
| A0A2K5UC74 | Polyadenylate-binding protein OS=Macaca fascicularis OX=9541 PE=3 SV=2 | LOC102118081 | pink | 0.334158 | 0.328852 |
| Q4R5N4 | Ubiquitin-conjugating enzyme E2 D3 OS=Macaca fascicularis OX=9541 GN=UBE2D3 PE=2 SV=1 | UBE2D3 | pink | 0.319211 | 0.294589 |
| A0A7N9CHP7 | IGv domain-containing protein OS=Macaca fascicularis OX=9541 PE=4 SV=1 | LOC102139381 | pink | 0.289345 | 0.21252 |
| A0A2K5W3N9 | Rho GTPase activating protein 1 OS=Macaca fascicularis OX=9541 GN=ARHGAP1 PE=4 SV=2 | ARHGAP1 | pink | 0.272325 | 0.244388 |
| A0A7N9D8T9 | Ig-like domain-containing protein OS=Macaca fascicularis OX=9541 PE=4 SV=1 | LOC102129827 | pink | 0.238053 | 0.12869 |
| A0A7N9CH17 | Ig-like domain-containing protein OS=Macaca fascicularis OX=9541 PE=4 SV=1 | LOC102145803 | pink | 0.221367 | 0.210637 |
| A0A2K5WCE4 | Moesin OS=Macaca fascicularis OX=9541 GN=MSN PE=4 SV=2 | MSN | pink | 0.192441 | 0.130142 |
| A0A2K5V6F2 | EH domain containing 3 OS=Macaca fascicularis OX=9541 GN=EHD3 PE=4 SV=1 | EHD3 | pink | 0.184118 | 0.191535 |
| A0A2K5X8Q4 | Microtubule-associated protein RP/EB family member 2 OS=Macaca fascicularis OX=9541 GN=MAPRE2 PE=3 SV=2 | MAPRE2 | pink | 0.14378 | -0.00199 |
| A0A2K5UQB4 | Penta-EF-hand domain containing 1 OS=Macaca fascicularis OX=9541 GN=PEF1 PE=4 SV=1 | PEF1 | pink | 0.127978 | 0.147114 |
| A0A2K5WZ75 | Transgelin OS=Macaca fascicularis OX=9541 GN=TAGLN2 PE=3 SV=1 | TAGLN2 | pink | 0.113748 | 0.079422 |
| A0A2K5VHH0 | Torsin OS=Macaca fascicularis OX=9541 GN=SH2D3C PE=3 SV=2 | TOR2A | pink | 0.044116 | 0.1054 |
| G7P145 | Mitochondrial fission 1 protein OS=Macaca fascicularis OX=9541 GN=EGM_12423 PE=3 SV=1 | FIS1 | pink | 0.016739 | 0.024767 |
| I7G9T4 | Macaca fascicularis brain cDNA clone: QtrA-18020, similar to human similar to FLJ27099 protein (LOC388078), mRNA, RefSeq: XM_370835.1 OS=Macaca fascicularis OX=9541 PE=2 SV=1 | LOC102142558 | pink | -0.06006 | -0.12329 |
| A0A2K5WU43 | Uncharacterized protein OS=Macaca fascicularis OX=9541 GN=EGM_04866 PE=4 SV=1 | PLEK | pink | -0.09968 | -0.18962 |
| Q76KA2 | 60S ribosomal protein L30 OS=Macaca fascicularis OX=9541 GN=RPL30 PE=3 SV=3 >tr\|A0A158SIP2\|A0A158SIP2_MACFA 60S ribosomal protein L30 OS=Macaca fascicularis OX=9541 PE=2 SV=1 | RPL30 | pink | -0.17814 | -0.07082 |
| A0A7N9IBR1 | Ig-like domain-containing protein OS=Macaca fascicularis OX=9541 PE=4 SV=1 | LOC102129829 | pink | -0.23169 | -0.27527 |
| A0A2K5VCL9 | Ig-like domain-containing protein OS=Macaca fascicularis OX=9541 PE=4 SV=2 | LOC107130946 | pink | -0.25141 | -0.21196 |
| A0A7N9D6F4 | Ig-like domain-containing protein OS=Macaca fascicularis OX=9541 PE=4 SV=1 | LOC102140914 | pink | -0.44591 | -0.39418 |
| G7NUF4 | Uncharacterized protein OS=Macaca fascicularis OX=9541 GN=EGM_01100 PE=3 SV=1 | SPRR1B | pink | -0.51779 | -0.49483 |

**Supplementary Table 5 All the proteins contained within the black block**

| Protein accession | Protein description | Gene name | Module | Abnormality_rate | Vertebral_abnomaly |
| --- | --- | --- | --- | --- | --- |
| I7GDD2 | Protein S100 OS=Macaca fascicularis OX=9541 PE=2 SV=1 | S100A1 | black | -0.13229 | -0.15134 |
| G7PYU9 | Complement C3 OS=Macaca fascicularis OX=9541 GN=EGM_09161 PE=4 SV=1 | C3 | black | -0.40516 | -0.41131 |
| G7PMG1 | GlcNAc kinase OS=Macaca fascicularis OX=9541 GN=NAGK PE=3 SV=1 | URS00021F207A | black | 0.040278 | 0.056245 |
| G7PIV1 | Activin beta-E chain OS=Macaca fascicularis OX=9541 GN=INHBE PE=3 SV=1 | INHBE | black | -0.41523 | -0.36576 |
| G7PID3 | Cytokeratin-2e OS=Macaca fascicularis OX=9541 GN=KRT1 PE=3 SV=1 | KRT2 | black | 0.176294 | 0.06492 |
| G7P9U7 | Proteasome subunit beta OS=Macaca fascicularis OX=9541 GN=PSMB5 PE=3 SV=1 | PSMB5 | black | -0.45863 | -0.38672 |
| G7P7F1 | Complement component C6 OS=Macaca fascicularis OX=9541 GN=EGM_15009 PE=3 SV=1 | C6 | black | -0.1167 | -0.14035 |
| G7P4A9 | Proteasome subunit beta OS=Macaca fascicularis OX=9541 GN=EGM_14190 PE=3 SV=1 | PSMB1 | black | -0.45063 | -0.33972 |
| G7NU00 | Sulfhydryl oxidase OS=Macaca fascicularis OX=9541 GN=EGM_01627 PE=3 SV=1 | QSOX1 | black | 0.538033 | 0.592932 |
| G7NTT3 | Extracellular matrix protein 1 OS=Macaca fascicularis OX=9541 GN=ECM1 PE=4 SV=1 | ECM1 | black | -0.57838 | -0.66703 |
| A2V9Z9 | Uncharacterized protein OS=Macaca fascicularis OX=9541 PE=2 SV=1 | GPX3 | black | 0.434955 | 0.384803 |
| A0A7N9ICI4 | Ig-like domain-containing protein OS=Macaca fascicularis OX=9541 PE=4 SV=1 | LOC102140532 | black | 0.031897 | 0.025525 |
| A0A7N9I9E5 | Uncharacterized protein OS=Macaca fascicularis OX=9541 PE=4 SV=1 | LOC102136483 | black | 0.514404 | 0.519443 |
| A0A7N9DA15 | Proteasome subunit beta OS=Macaca fascicularis OX=9541 GN=PSMB2 PE=3 SV=1 | PSMB2 | black | -0.39464 | -0.35367 |
| A0A7N9D4M8 | Ig-like domain-containing protein OS=Macaca fascicularis OX=9541 PE=4 SV=1 | LOC102140914 | black | -0.10123 | -0.08956 |
| A0A7N9CGC8 | Alpha-1,4-N-acetylglucosaminyltransferase OS=Macaca fascicularis OX=9541 GN=A4GALT PE=3 SV=1 | A4GALT | black | -0.41016 | -0.40988 |
| A0A7N9CFS6 | Ig-like domain-containing protein OS=Macaca fascicularis OX=9541 PE=4 SV=1 | LOC102128954 | black | -0.36014 | -0.387 |
| A0A7N9C6U5 | Ig-like domain-containing protein OS=Macaca fascicularis OX=9541 PE=4 SV=1 | LOC102136084 | black | 0.382551 | 0.339417 |
| A0A2K5WXZ3 | Serpin family A member 6 OS=Macaca fascicularis OX=9541 GN=SERPINA6 PE=3 SV=2 | SERPINA6 | black | 0.115932 | 0.165929 |
| A0A2K5WSB6 | Transcobalamin 2 OS=Macaca fascicularis OX=9541 GN=TCN2 PE=3 SV=2 | TCN2 | black | -0.1456 | -0.11843 |
| A0A2K5WPR8 | Actin-related protein 2/3 complex subunit 4 OS=Macaca fascicularis OX=9541 GN=ARPC4 PE=3 SV=1 | ARPC4 | black | 0.615004 | 0.571474 |
| A0A2K5WLR2 | Ovochymase 2 OS=Macaca fascicularis OX=9541 GN=OVCH2 PE=4 SV=2 | OVCH2 | black | 0.608824 | 0.632028 |
| A0A2K5WKN3 | Angiopoietin like 2 OS=Macaca fascicularis OX=9541 GN=ANGPTL2 PE=4 SV=2 >tr\|G8F2W1\|G8F2W1_MACFA Angiopoietin-like protein 2 OS=Macaca fascicularis OX=9541 GN=EGM_19645 PE=4 SV=1 | ANGPTL2 | black | -0.43916 | -0.43916 |
| A0A2K5W6A0 | Proteasome subunit alpha type OS=Macaca fascicularis OX=9541 PE=3 SV=1 | PSMA3 | black | -0.89743 | -0.89743 |
| A0A2K5W2F7 | Mannan binding lectin serine peptidase 1 OS=Macaca fascicularis OX=9541 GN=MASP1 PE=4 SV=2 | MASP1 | black | 0.182774 | 0.121925 |
| A0A2K5VW23 | Periostin OS=Macaca fascicularis OX=9541 GN=POSTN PE=4 SV=2 | POSTN | black | -0.25618 | -0.35327 |
| A0A2K5VRH6 | Uncharacterized protein OS=Macaca fascicularis OX=9541 PE=4 SV=2 | DSC2 | black | -0.32453 | -0.32944 |
| A0A2K5VRH1 | Alpha-mannosidase OS=Macaca fascicularis OX=9541 GN=MAN2A1 PE=3 SV=2 | MAN2A1 | black | -0.49738 | -0.39139 |
| A0A2K5VPD5 | PDGF_2 domain-containing protein OS=Macaca fascicularis OX=9541 PE=3 SV=2 | PDGFA | black | 0.108433 | 0.144603 |
| A0A2K5VNN1 | CD109 molecule OS=Macaca fascicularis OX=9541 GN=CD109 PE=3 SV=2 | CD109 | black | -0.10034 | -0.11939 |
| A0A2K5V8G9 | Proteasome subunit beta OS=Macaca fascicularis OX=9541 GN=EGM_19907 PE=3 SV=1 >tr\|G8F2D0\|G8F2D0_MACFA Proteasome subunit beta OS=Macaca fascicularis OX=9541 GN=PSMB4 PE=3 SV=1 | PSMB4 | black | -0.42033 | -0.40829 |
| A0A2K5V898 | Thrombospondin 4 OS=Macaca fascicularis OX=9541 GN=THBS4 PE=3 SV=2 | THBS4 | black | -0.3417 | -0.36795 |
| A0A2K5UWF8 | Protein phosphatase 3 regulatory subunit B, alpha OS=Macaca fascicularis OX=9541 PE=3 SV=1 | PPP3R1 | black | 0.363911 | 0.160162 |
| A0A2K5UTU0 | Ig-like domain-containing protein OS=Macaca fascicularis OX=9541 PE=4 SV=2 | LOC102142507 | black | 0.356701 | 0.370939 |
| A0A2K5UTB9 | Rab GDP dissociation inhibitor OS=Macaca fascicularis OX=9541 GN=GDI2 PE=3 SV=2 | GDI2 | black | -0.47061 | -0.45466 |
| A0A2K5USD8 | Trefoil factor 1 OS=Macaca fascicularis OX=9541 GN=TFF1 PE=4 SV=2 | TFF1 | black | -0.27125 | -0.26595 |
| A0A2K5URU5 | Twinfilin actin binding protein 2 OS=Macaca fascicularis OX=9541 GN=TWF2 PE=3 SV=2 | LOC102144128 | black | 0.107791 | -0.03149 |
| A0A2K5UP93 | Meteorin, glial cell differentiation regulator OS=Macaca fascicularis OX=9541 GN=METRN PE=4 SV=2 | METRN | black | -0.30013 | -0.30851 |
| A0A2K5UL99 | Thymosin beta OS=Macaca fascicularis OX=9541 GN=TMSB4X PE=3 SV=2 | TMSB4X | black | -0.45235 | -0.39738 |
| A0A2K5UJD0 | Elongin B OS=Macaca fascicularis OX=9541 GN=ELOB PE=4 SV=1 | TCEB2 | black | -0.08123 | -0.24981 |
| A0A2K5U9A1 | Fucose mutarotase OS=Macaca fascicularis OX=9541 GN=FUOM PE=4 SV=2 | FUOM | black | -0.25543 | -0.21638 |
| A0A2K5U998 | Alpha-L-fucosidase I OS=Macaca fascicularis OX=9541 GN=FUCA1 PE=3 SV=1 | FUCA1 | black | 0.035546 | 0.135352 |
| A0A2K5U931 | Ig-like domain-containing protein OS=Macaca fascicularis OX=9541 PE=4 SV=2 | LOC107130949 | black | -0.18375 | -0.18784 |
| A0A2K5U515 | Peptidylglycine alpha-amidating monooxygenase OS=Macaca fascicularis OX=9541 GN=PAM PE=3 SV=2 | PAM | black | -0.6209 | -0.63805 |
| A0A2K5U4H6 | Thy-1 antigen OS=Macaca fascicularis OX=9541 GN=THY1 PE=4 SV=1 | THY1 | black | -0.1314 | -0.26068 |
| A0A2K5U480 | Inter-alpha-trypsin inhibitor heavy chain 1 OS=Macaca fascicularis OX=9541 GN=ITIH1 PE=3 SV=2 | ITIH1 | black | -0.42959 | -0.40645 |
| A0A2K5U0M2 | Proteasome subunit beta OS=Macaca fascicularis OX=9541 GN=TAP1 PE=3 SV=2 | PSMB8 | black | 0.046337 | 0.077359 |
| A0A2K5TV89 | Vitelline membrane outer layer 1 homolog OS=Macaca fascicularis OX=9541 GN=VMO1 PE=4 SV=2 | VMO1 | black | -0.40992 | -0.3368 |
| A0A2K5TNF2 | Ig-like domain-containing protein OS=Macaca fascicularis OX=9541 PE=4 SV=2 | LOC102141543 | black | 0.1167 | 0.12212 |
| A0A2K5TN05 | Peptidylprolyl isomerase OS=Macaca fascicularis OX=9541 PE=4 SV=2 | FKBP3 | black | -0.15101 | -0.15602 |
| A0A172R2J0 | Rearranged Ig variable region VDJ (Fragment) OS=Macaca fascicularis OX=9541 GN=IGH PE=2 SV=1 | LOC102130358 | black | 0.367046 | 0.434176 |
| A0A172R2G9 | Rearranged Ig variable region VDJ (Fragment) OS=Macaca fascicularis OX=9541 GN=IGH PE=2 SV=1 | LOC102134353 | black | 0.289964 | 0.171581 |
| Q7YQD7 | EGF-containing fibulin-like extracellular matrix protein 1 OS=Macaca fascicularis OX=9541 GN=EFEMP1 PE=2 SV=1 | EFEMP1 | black | 0.093819 | 0.148885 |
| Q60HG6 | Dipeptidyl peptidase 1 OS=Macaca fascicularis OX=9541 GN=CTSC PE=2 SV=1 | CTSC | black | 0.136728 | 0.127731 |
| Q4R3D4 | Elongation factor 1-delta OS=Macaca fascicularis OX=9541 GN=EEF1D PE=2 SV=2 | EEF1D | black | 0.645623 | 0.564003 |

**Supplementary Table 6 All the proteins contained within the blue block**

| Protein accession | Protein description | Gene name | Module | Abnormality_rate | Vertebral_abnomaly |
| --- | --- | --- | --- | --- | --- |
| A0A2K5V886 | Lipopolysaccharide-binding protein OS=Macaca fascicularis OX=9541 GN=EGM_02099 PE=3 SV=1 | LBP | blue | 0.629333 | 0.656033 |
| A0A2K5TWD9 | Multimerin 1 OS=Macaca fascicularis OX=9541 GN=MMRN1 PE=4 SV=2 | MMRN1 | blue | 0.582721 | 0.642348 |
| P61269 | Metalloproteinase inhibitor 3 OS=Macaca fascicularis OX=9541 GN=TIMP3 PE=2 SV=1 | TIMP3 | blue | 0.54928 | 0.608314 |
| A0A2K5WZC5 | Phospholipase B-like OS=Macaca fascicularis OX=9541 GN=PLBD2 PE=3 SV=2 | PLBD2 | blue | 0.534627 | 0.5698 |
| A0A2K5WK85 | Serpin family A member 5 OS=Macaca fascicularis OX=9541 GN=SERPINA5 PE=3 SV=2 | SERPINA5 | blue | 0.414307 | 0.474847 |
| A0A2K5TVP3 | Zinc finger and BTB domain containing 48 OS=Macaca fascicularis OX=9541 GN=ZBTB48 PE=4 SV=2 | ZBTB48 | blue | 0.403366 | 0.448744 |
| A0A7N9CMB1 | Immunoglobulin lambda variable 9-49 OS=Macaca fascicularis OX=9541 PE=4 SV=1 | LOC107126465 | blue | 0.362297 | 0.377193 |
| A0A2K5V505 | Oncoprotein induced transcript 3 OS=Macaca fascicularis OX=9541 GN=OIT3 PE=4 SV=2 | OIT3 | blue | 0.237219 | 0.242897 |
| G7Q1J3 | Haptoglobin OS=Macaca fascicularis OX=9541 GN=EGM_11911 PE=3 SV=1 | LOC102131765 | blue | 0.235076 | 0.280381 |
| Q4R5B6 | L-lactate dehydrogenase B chain OS=Macaca fascicularis OX=9541 GN=LDHB PE=2 SV=3 >tr\|G7PCH4\|G7PCH4_MACFA L-lactate dehydrogenase OS=Macaca fascicularis OX=9541 GN=EGM_17551 PE=3 SV=1 | LOC102117828 | blue | 0.137844 | 0.129333 |
| A0A2K5TZ70 | Ig-like domain-containing protein OS=Macaca fascicularis OX=9541 PE=4 SV=2 | LOC102131401 | blue | 0.007986 | -0.01523 |
| A0A2K5UU92 | U6 snRNA-associated Sm-like protein LSm4 OS=Macaca fascicularis OX=9541 GN=LSM4 PE=3 SV=2 | LSM4 | blue | -0.00678 | 0.013723 |
| Q4R4R6 | Cell division control protein 42 homolog OS=Macaca fascicularis OX=9541 GN=CDC42 PE=2 SV=1 | CDC42 | blue | -0.01407 | -0.08324 |
| A0A7N9CIG3 | Aldo_ket_red domain-containing protein OS=Macaca fascicularis OX=9541 PE=4 SV=1 | LOC102144847 | blue | -0.01686 | -0.06699 |
| A0A2K5TM46 | NSF attachment protein alpha OS=Macaca fascicularis OX=9541 PE=3 SV=2 | NAPA | blue | -0.03258 | 0.15859 |
| A0A2K5WE57 | Collagen type I alpha 1 chain OS=Macaca fascicularis OX=9541 GN=COL1A1 PE=4 SV=2 | COL1A1 | blue | -0.05258 | -0.11218 |
| Q8HXW4 | Glycogen phosphorylase, muscle form OS=Macaca fascicularis OX=9541 GN=PYGM PE=2 SV=3 | PYGM | blue | -0.05752 | -0.19345 |
| A0A7N9DH80 | Ig-like domain-containing protein OS=Macaca fascicularis OX=9541 PE=4 SV=1 | LOC102138638 | blue | -0.08114 | -0.0455 |
| G7PRG1 | Lipocln_cytosolic_FA-bd_dom domain-containing protein OS=Macaca fascicularis OX=9541 GN=EGM_06639 PE=3 SV=1 | LCN2 | blue | -0.08964 | -0.04311 |
| A0A2K5V9B1 | Ig-like domain-containing protein OS=Macaca fascicularis OX=9541 PE=4 SV=2 | LOC107130946 | blue | -0.10463 | -0.16478 |
| G8F5L2 | Uncharacterized protein OS=Macaca fascicularis OX=9541 GN=EGM_20969 PE=3 SV=1 | DDT | blue | -0.10798 | -0.24979 |
| A0A7N9IBW1 | Ig-like domain-containing protein OS=Macaca fascicularis OX=9541 PE=4 SV=1 | LOC102121852 | blue | -0.11207 | -0.20274 |
| A0A2K5U4J3 | Complement C1r subcomponent like OS=Macaca fascicularis OX=9541 GN=C1RL PE=4 SV=2 | C1RL | blue | -0.12845 | -0.18295 |
| A0A2K5VUH9 | Glucosidase II alpha subunit OS=Macaca fascicularis OX=9541 PE=3 SV=1 | GANAB | blue | -0.14739 | -0.16421 |
| A0A2K5WW23 | Lipoprotein lipase OS=Macaca fascicularis OX=9541 PE=3 SV=2 | LPL | blue | -0.14771 | -0.1731 |
| A0A2K5WGG3 | Glucosamine-6-phosphate isomerase OS=Macaca fascicularis OX=9541 GN=GNPDA2 PE=3 SV=2 | GNPDA2 | blue | -0.15775 | -0.25979 |
| A0A2K5U0I4 | C3/C5 convertase OS=Macaca fascicularis OX=9541 GN=EGM_20128 PE=4 SV=1 >tr\|G8F3W0\|G8F3W0_MACFA C3/C5 convertase OS=Macaca fascicularis OX=9541 GN=C2 PE=4 SV=1 | C2 | blue | -0.16325 | -0.11632 |
| G7PXY0 | Peptidoglycan-recognition protein OS=Macaca fascicularis OX=9541 GN=EGM_09885 PE=3 SV=1 | PGLYRP1 | blue | -0.18232 | -0.21079 |
| A0A2K5WYC7 | Peroxiredoxin 3 OS=Macaca fascicularis OX=9541 PE=4 SV=2 | PRDX3 | blue | -0.18508 | -0.29181 |
| Q4R5C0 | Cofilin-1 OS=Macaca fascicularis OX=9541 GN=CFL1 PE=2 SV=3 | CFL1 | blue | -0.2109 | -0.28492 |
| A0A2K5VD26 | Family with sequence similarity 180 member B OS=Macaca fascicularis OX=9541 GN=FAM180B PE=4 SV=2 | FAM180B | blue | -0.21218 | -0.16468 |
| G7PLE3 | Insulin-like growth factor-binding protein 5 OS=Macaca fascicularis OX=9541 GN=EGM_04300 PE=4 SV=1 | IGFBP5 | blue | -0.22911 | -0.28505 |
| A0A7N9CS44 | Ig-like domain-containing protein OS=Macaca fascicularis OX=9541 PE=4 SV=1 | LOC102143543 | blue | -0.23483 | -0.25072 |
| A0A2K5UF05 | Ig-like domain-containing protein OS=Macaca fascicularis OX=9541 PE=4 SV=1 | LOC102132176 | blue | -0.24684 | -0.19327 |
| G7PHC9 | Ig-like domain-containing protein (Fragment) OS=Macaca fascicularis OX=9541 GN=EGM_02499 PE=4 SV=1 | LOC102121852 | blue | -0.25712 | -0.30588 |
| I7GDN5 | Macaca fascicularis brain cDNA clone: QflA-23190, similar to human transferrin (TF), mRNA, RefSeq: NM_001063.2 OS=Macaca fascicularis OX=9541 PE=2 SV=1 | LOC101866521 | blue | -0.25853 | -0.3586 |
| A0A2K5V7P0 | C-C motif chemokine OS=Macaca fascicularis OX=9541 PE=3 SV=2 | LOC102134112 | blue | -0.26881 | -0.30021 |
| G7Q2L6 | Complement factor P OS=Macaca fascicularis OX=9541 GN=EGM_18726 PE=4 SV=1 | CFP | blue | -0.28418 | -0.34656 |
| A0A2K5VVW4 | Coagulation factor XI OS=Macaca fascicularis OX=9541 GN=F11 PE=4 SV=1 | F11 | blue | -0.2873 | -0.28747 |
| A0A2K5X990 | Fibrinogen like 1 OS=Macaca fascicularis OX=9541 GN=FGL1 PE=4 SV=1 | FGL1 | blue | -0.29437 | -0.23778 |
| A0A2K5WMN9 | Tyrosine 3-monooxygenase/tryptophan 5-monooxygenase activation protein gamma OS=Macaca fascicularis OX=9541 GN=YWHAG PE=3 SV=2 | YWHAG | blue | -0.30515 | -0.35917 |
| A0A2K5W6R2 | C1q domain-containing protein OS=Macaca fascicularis OX=9541 GN=EGM_14973 PE=4 SV=1 | C1QTNF3 | blue | -0.3077 | -0.26982 |
| G7PN15 | Ig-like domain-containing protein (Fragment) OS=Macaca fascicularis OX=9541 GN=EGM_05146 PE=4 SV=1 | LOC102143278 | blue | -0.30865 | -0.42552 |
| A0A2K5W5A4 | 72 kDa gelatinase OS=Macaca fascicularis OX=9541 GN=MMP2 PE=3 SV=1 | MMP2 | blue | -0.31331 | -0.31331 |
| A0A2K5UPU4 | PDZ and LIM domain 5 OS=Macaca fascicularis OX=9541 GN=PDLIM5 PE=4 SV=2 | PDLIM5 | blue | -0.31622 | -0.34608 |
| G7P2L7 | H1.2 linker histone, cluster member OS=Macaca fascicularis OX=9541 GN=H1-2 PE=3 SV=1 | LOC102138750 | blue | -0.32147 | -0.28591 |
| A0A171P0Z8 | Carboxylic ester hydrolase OS=Macaca fascicularis OX=9541 PE=2 SV=1 | LOC102119346 | blue | -0.32345 | -0.36312 |
| A0A2K5TM01 | HIT domain-containing protein OS=Macaca fascicularis OX=9541 PE=3 SV=1 | HINT1 | blue | -0.32835 | -0.39125 |
| A0A2K5URR6 | Insulin-like growth factor I OS=Macaca fascicularis OX=9541 GN=IGF1 PE=3 SV=1 | IGF1 | blue | -0.33226 | -0.51313 |
| A0A2K5WM93 | Inter-alpha-trypsin inhibitor heavy chain 4 OS=Macaca fascicularis OX=9541 GN=ITIH4 PE=3 SV=1 | ITIH4 | blue | -0.33261 | -0.36236 |
| A0A2K5UJF6 | Ig-like domain-containing protein OS=Macaca fascicularis OX=9541 PE=4 SV=1 | LOC107131040 | blue | -0.34377 | -0.25371 |
| A0A7N9D4Z4 | Lysyl oxidase homolog OS=Macaca fascicularis OX=9541 GN=LOX PE=3 SV=1 | LOX | blue | -0.35932 | -0.48928 |
| A0A2K5UDC5 | Angiopoietin like 3 OS=Macaca fascicularis OX=9541 GN=ANGPTL3 PE=4 SV=1 | ANGPTL3 | blue | -0.36411 | -0.44579 |
| A0A2K5U716 | Cathepsin X OS=Macaca fascicularis OX=9541 GN=CTSZ PE=3 SV=2 | CTSZ | blue | -0.36783 | -0.3175 |
| A0A2K5TTI7 | Enoyl-CoA delta isomerase 1 OS=Macaca fascicularis OX=9541 GN=ECI1 PE=3 SV=2 | ECI1 | blue | -0.38567 | -0.40276 |
| A0A2K5WJV6 | Complement C1q subcomponent subunit A OS=Macaca fascicularis OX=9541 GN=C1QA PE=4 SV=2 | C1QA | blue | -0.40066 | -0.34942 |
| A0A2K5WWZ6 | Tyrosine 3-monooxygenase/tryptophan 5-monooxygenase activation protein epsilon OS=Macaca fascicularis OX=9541 GN=YWHAE PE=3 SV=1 | YWHAE | blue | -0.40306 | -0.42281 |
| A0A2K5V4B0 | Apolipoprotein H OS=Macaca fascicularis OX=9541 PE=4 SV=2 | APOH | blue | -0.40436 | -0.42663 |
| A0A172R2E0 | Rearranged Ig variable region VDJ (Fragment) OS=Macaca fascicularis OX=9541 GN=IGH PE=2 SV=1 | LOC102128954 | blue | -0.40908 | -0.46398 |
| A0A7N9D0Y9 | Serine protease 23 OS=Macaca fascicularis OX=9541 GN=PRSS23 PE=4 SV=1 | PRSS23 | blue | -0.43317 | -0.43625 |
| A0A2K5V473 | Ficolin 3 OS=Macaca fascicularis OX=9541 GN=FCN3 PE=4 SV=2 | FCN3 | blue | -0.4344 | -0.50423 |
| A0A2K5X4G5 | von Willebrand factor OS=Macaca fascicularis OX=9541 GN=VWF PE=4 SV=2 | VWF | blue | -0.43695 | -0.5025 |
| G7PYL2 | Leukemia/lymphoma-related factor OS=Macaca fascicularis OX=9541 GN=EGM_09102 PE=4 SV=1 | ZBTB7A | blue | -0.43835 | -0.52558 |
| A0A2K5VHC7 | Appetite-regulating hormone OS=Macaca fascicularis OX=9541 GN=GHRL PE=3 SV=1 | GHRL | blue | -0.44004 | -0.39225 |
| G7PHT9 | Insulin-like growth factor-binding protein 6 OS=Macaca fascicularis OX=9541 GN=IGFBP6 PE=4 SV=1 | IGFBP6 | blue | -0.44539 | -0.50916 |
| A0A0G4NS53 | B protein OS=Macaca fascicularis OX=9541 GN=Mafa-B PE=2 SV=1 | LOC102117143 | blue | -0.45246 | -0.397 |
| Q4R5M2 | Cathepsin B OS=Macaca fascicularis OX=9541 GN=CTSB PE=2 SV=1 >tr\|G7PCE3\|G7PCE3_MACFA Cathepsin B OS=Macaca fascicularis OX=9541 GN=EGM_17073 PE=3 SV=1 | CTSB | blue | -0.45362 | -0.46868 |
| A0A2K5X7W4 | Catalase OS=Macaca fascicularis OX=9541 GN=CAT PE=3 SV=1 | CAT | blue | -0.45434 | -0.40352 |
| G7NUP6 | Uncharacterized protein OS=Macaca fascicularis OX=9541 GN=EGM_01442 PE=4 SV=1 | C4BPB | blue | -0.45655 | -0.45371 |
| Q2PFX4 | Histone H2B type 1-K OS=Macaca fascicularis OX=9541 GN=H2BC12 PE=2 SV=3 | LOC102137744 | blue | -0.46423 | -0.52001 |
| A0A2K5VEE8 | Glucose-6-phosphate 1-dehydrogenase OS=Macaca fascicularis OX=9541 GN=G6PD PE=3 SV=2 | G6PD | blue | -0.46657 | -0.44222 |
| A0A2K5TSB5 | Aldo_ket_red domain-containing protein OS=Macaca fascicularis OX=9541 PE=4 SV=2 | LOC102144847 | blue | -0.46738 | -0.59753 |
| A0A2K5VTV5 | Dermokine OS=Macaca fascicularis OX=9541 GN=DMKN PE=4 SV=2 | DMKN | blue | -0.47646 | -0.53578 |
| A0A2K5VB66 | Glutathione peroxidase OS=Macaca fascicularis OX=9541 GN=GPX1 PE=3 SV=1 | GPX1 | blue | -0.48011 | -0.53244 |
| A0A2K5TYK2 | Transforming growth factor beta receptor 3 OS=Macaca fascicularis OX=9541 GN=TGFBR3 PE=4 SV=2 | TGFBR3 | blue | -0.48548 | -0.55933 |
| A0A2K5TSB3 | Cytidine deaminase OS=Macaca fascicularis OX=9541 GN=EGM_00303 PE=3 SV=1 | CDA | blue | -0.48771 | -0.51201 |
| G7PWK2 | Desmocollin-4 OS=Macaca fascicularis OX=9541 GN=EGM_08770 PE=4 SV=1 | DSC3 | blue | -0.52241 | -0.54275 |
| A0A7N9C8Q6 | UPAR/Ly6 domain-containing protein OS=Macaca fascicularis OX=9541 PE=4 SV=1 | LOC102133480 | blue | -0.53187 | -0.5922 |
| A0A0A8R5S8 | MHC class II protein OS=Macaca fascicularis OX=9541 GN=Mafa-DRA PE=2 SV=1 | LOC102144039 | blue | -0.53559 | -0.61498 |
| G7NUE7 | Guanylate cyclase activator 2B OS=Macaca fascicularis OX=9541 GN=GUCA2B PE=3 SV=1 | GUCA2B | blue | -0.53695 | -0.5133 |
| A0A2K5VGW3 | Hyaluronidase OS=Macaca fascicularis OX=9541 GN=HYAL3 PE=3 SV=1 | HYAL3 | blue | -0.53794 | -0.63416 |
| A0A2K5V0E3 | Fibronectin OS=Macaca fascicularis OX=9541 GN=FN1 PE=4 SV=1 | FN1 | blue | -0.53891 | -0.64521 |
| A0A2K5VNF3 | Pyruvate kinase OS=Macaca fascicularis OX=9541 PE=3 SV=2 | PKM | blue | -0.54263 | -0.48337 |
| Q9TSM4 | Glutathione S-transferase Mu 2 OS=Macaca fascicularis OX=9541 GN=GSTM2 PE=1 SV=3 | GSTM2 | blue | -0.55283 | -0.55804 |
| A0A2K5U897 | Suprabasin OS=Macaca fascicularis OX=9541 GN=SBSN PE=4 SV=1 | SBSN | blue | -0.56433 | -0.55353 |
| A0A2K5WUZ3 | Protein AMBP OS=Macaca fascicularis OX=9541 GN=EGM_06710 PE=3 SV=1 | AMBP | blue | -0.56467 | -0.56187 |
| A0A2K5X0P4 | Phosphatidylethanolamine binding protein 4 OS=Macaca fascicularis OX=9541 GN=PEBP4 PE=3 SV=2 | PEBP4 | blue | -0.58321 | -0.59116 |
| A0A2K5TX64 | Fatty acid binding protein 6 OS=Macaca fascicularis OX=9541 GN=FABP6 PE=3 SV=1 | FABP6 | blue | -0.58801 | -0.67594 |
| A0A7N9D443 | Adipsin OS=Macaca fascicularis OX=9541 PE=4 SV=1 | CFD | blue | -0.58844 | -0.65152 |
| A0A2K5WIY3 | Neuroblastoma suppressor of tumorigenicity 1 OS=Macaca fascicularis OX=9541 GN=NBL1 PE=3 SV=1 | NBL1 | blue | -0.59057 | -0.61332 |
| A0A2K5V5A5 | Gastrokine 1 OS=Macaca fascicularis OX=9541 GN=GKN1 PE=4 SV=1 | GKN1 | blue | -0.62049 | -0.63121 |
| A0A2K5W5E1 | Ly6/neurotoxin 1 OS=Macaca fascicularis OX=9541 PE=4 SV=1 | LYNX1 | blue | -0.6228 | -0.64593 |
| Q8HXP3 | Superoxide dismutase [Mn], mitochondrial OS=Macaca fascicularis OX=9541 GN=SOD2 PE=2 SV=3 | SOD2 | blue | -0.63745 | -0.60473 |
| A0A2K5VUU4 | Uncharacterized protein OS=Macaca fascicularis OX=9541 GN=EGM_10806 PE=4 SV=1 | FETUB | blue | -0.65883 | -0.66397 |
| G8F2P8 | Ig-like domain-containing protein (Fragment) OS=Macaca fascicularis OX=9541 GN=EGM_19491 PE=4 SV=1 | LOC102131004 | blue | -0.66045 | -0.59075 |
| A0A7N9CVY3 | Secreted phosphoprotein 2 OS=Macaca fascicularis OX=9541 GN=SPP2 PE=3 SV=1 | SPP2 | blue | -0.66229 | -0.70513 |
| A0A2K5VQJ9 | EF-hand domain-containing protein OS=Macaca fascicularis OX=9541 PE=4 SV=2 | EFHD2 | blue | -0.67062 | -0.64057 |
| A0A2K5W448 | Galectin OS=Macaca fascicularis OX=9541 GN=PDXP PE=4 SV=1 | LGALS1 | blue | -0.69368 | -0.76003 |
| A0A2K5W2G8 | Apolipoprotein F OS=Macaca fascicularis OX=9541 GN=EGM_03387 PE=4 SV=1 | APOF | blue | -0.71414 | -0.69648 |
| Q8HXX6 | Ganglioside GM2 activator OS=Macaca fascicularis OX=9541 GN=GM2A PE=2 SV=2 | GM2A | blue | -0.71715 | -0.79381 |
| A0A2K5TZE8 | Aldo_ket_red domain-containing protein OS=Macaca fascicularis OX=9541 PE=3 SV=2 | LOC101865018 | blue | -0.71719 | -0.7739 |
| A0A2K5VJW0 | CutA divalent cation tolerance homolog OS=Macaca fascicularis OX=9541 GN=CUTA PE=3 SV=1 | CUTA | blue | -0.7542 | -0.75779 |
| A0A2K5VAC4 | Uncharacterized protein OS=Macaca fascicularis OX=9541 PE=4 SV=2 | DSC1 | blue | -0.85319 | -0.87241 |

**Supplementary Table 7 All the proteins contained within the brown block**

| Protein accession | Protein description | Gene name | Module | Abnormality_rate | Vertebral_abnomaly |
| --- | --- | --- | --- | --- | --- |
| I7GM68 | Clusterin OS=Macaca fascicularis OX=9541 PE=2 SV=1 | CLU | brown | -0.05021 | -0.11692 |
| G8F6J7 | NTR domain-containing protein (Fragment) OS=Macaca fascicularis OX=9541 GN=EGM_00034 PE=4 SV=1 | LOC102132156 | brown | 0.267628 | 0.240207 |
| G8F6B4 | Ig-like domain-containing protein (Fragment) OS=Macaca fascicularis OX=9541 GN=EGM_19501 PE=4 SV=1 | LOC102142558 | brown | 0.194831 | 0.270013 |
| G7Q062 | LRRNT domain-containing protein OS=Macaca fascicularis OX=9541 GN=EGM_11333 PE=4 SV=1 | IGFALS | brown | -0.17752 | -0.2068 |
| G7PYC5 | TGF_BETA_2 domain-containing protein (Fragment) OS=Macaca fascicularis OX=9541 GN=EGM_09042 PE=3 SV=1 | AMH | brown | 0.484964 | 0.374776 |
| G7PUW6 | Amine oxidase OS=Macaca fascicularis OX=9541 GN=EGM_07954 PE=3 SV=1 | LOC102140322 | brown | 0.00244 | -0.00677 |
| G7PR74 | Fibrinogen C-terminal domain-containing protein OS=Macaca fascicularis OX=9541 GN=EGM_06538 PE=3 SV=1 | LOC102131557 | brown | -0.04521 | -0.04949 |
| G7PMM8 | Uncharacterized protein OS=Macaca fascicularis OX=9541 GN=EGM_04982 PE=4 SV=1 | SFTPB | brown | -0.40413 | -0.43695 |
| G7PM02 | Uncharacterized protein (Fragment) OS=Macaca fascicularis OX=9541 GN=EGM_04713 PE=4 SV=1 | LTBP1 | brown | 0.363052 | 0.378936 |
| G7PLQ1 | Vitellogenin domain-containing protein OS=Macaca fascicularis OX=9541 GN=EGM_04602 PE=4 SV=1 | APOB | brown | -0.27728 | -0.33819 |
| G7PJR6 | C3 and PZP-like alpha-2-macroglobulin domain-containing protein 6 (Fragment) OS=Macaca fascicularis OX=9541 GN=EGM_02954 PE=3 SV=1 | LOC102133131 | brown | -0.27443 | -0.16754 |
| G7PDQ7 | Uncharacterized protein OS=Macaca fascicularis OX=9541 GN=EGM_18272 PE=3 SV=1 | CPN1 | brown | -0.0056 | -0.0215 |
| G7P8Z5 | Ig-like domain-containing protein (Fragment) OS=Macaca fascicularis OX=9541 GN=EGM_17028 PE=4 SV=1 | LOC102120126 | brown | 0.060416 | 0.080364 |
| G7P5L5 | Alpha-albumin OS=Macaca fascicularis OX=9541 GN=EGM_14424 PE=4 SV=1 | AFM | brown | -0.38037 | -0.36479 |
| G7P4P9 | Apolipoprotein M OS=Macaca fascicularis OX=9541 GN=APOM PE=3 SV=2 | APOM | brown | -0.14206 | -0.13865 |
| G7P0L3 | Leptin OS=Macaca fascicularis OX=9541 GN=LEP PE=3 SV=1 | LEP | brown | -0.3122 | -0.319 |
| G7NWI7 | Uncharacterized protein (Fragment) OS=Macaca fascicularis OX=9541 GN=EGM_01567 PE=4 SV=1 | CFH | brown | -0.37461 | -0.34634 |
| B3Y6B7 | Monocyte differentiation antigen CD14 OS=Macaca fascicularis OX=9541 GN=CD14 PE=2 SV=1 | CD14 | brown | -0.23103 | -0.17362 |
| A0A7N9IH44 | Syndecan OS=Macaca fascicularis OX=9541 PE=3 SV=1 | SDC1 | brown | 0.282221 | 0.103824 |
| A0A7N9ID20 | Ig-like domain-containing protein OS=Macaca fascicularis OX=9541 PE=4 SV=1 | LOC102143278 | brown | 0.317281 | 0.244797 |
| A0A7N9CZM4 | HtrA serine peptidase 2 OS=Macaca fascicularis OX=9541 GN=HTRA2 PE=3 SV=1 | HTRA2 | brown | -0.34552 | -0.28606 |
| A0A7N9CP14 | Ig-like domain-containing protein OS=Macaca fascicularis OX=9541 PE=4 SV=1 | LOC102143051 | brown | -0.30661 | -0.34384 |
| A0A7N9CNL5 | Immunoglobulin heavy constant mu OS=Macaca fascicularis OX=9541 GN=IGHM PE=4 SV=1 | LOC102116308 | brown | -0.09993 | -0.03677 |
| A0A7N9CG56 | UPAR/Ly6 domain-containing protein OS=Macaca fascicularis OX=9541 PE=4 SV=1 | LY6D | brown | -0.2597 | -0.24471 |
| A0A7N9CER8 | Uncharacterized protein OS=Macaca fascicularis OX=9541 PE=4 SV=1 | LPA | brown | -0.54098 | -0.53061 |
| A0A7N9CAW0 | Uncharacterized protein OS=Macaca fascicularis OX=9541 PE=4 SV=1 | LOC107126576 | brown | -0.0343 | 0.046446 |
| A0A7N9C7B5 | Insulin-like growth factor-binding protein 3 OS=Macaca fascicularis OX=9541 GN=IGFBP3 PE=4 SV=1 | IGFBP3 | brown | -0.47586 | -0.5157 |
| A0A2K5X975 | Prolactin OS=Macaca fascicularis OX=9541 GN=PRL PE=3 SV=2 | PRL | brown | -0.55837 | -0.56777 |
| A0A2K5X8X0 | Sex hormone-binding globulin OS=Macaca fascicularis OX=9541 GN=EGM_07374 PE=4 SV=1 | SHBG | brown | -0.38546 | -0.34577 |
| A0A2K5X8R4 | Dipeptidase OS=Macaca fascicularis OX=9541 GN=DPEP2 PE=3 SV=1 | DPEP2 | brown | 0.035491 | -0.0273 |
| A0A2K5X8L9 | Uncharacterized protein OS=Macaca fascicularis OX=9541 GN=EGM_02656 PE=4 SV=1 | RAC2 | brown | 0.257805 | 0.223918 |
| A0A2K5X7X6 | Adiponectin, C1Q and collagen domain containing OS=Macaca fascicularis OX=9541 GN=ADIPOQ PE=4 SV=1 | ADIPOQ | brown | 0.051296 | 0.046911 |
| A0A2K5X6Z8 | Ribosomal protein lateral stalk subunit P1 OS=Macaca fascicularis OX=9541 GN=RPLP1 PE=3 SV=1 | RPLP1 | brown | -0.60221 | -0.70025 |
| A0A2K5X3D3 | Protein XRP2 OS=Macaca fascicularis OX=9541 GN=RP2 PE=3 SV=1 | RP2 | brown | 0.423306 | 0.454613 |
| A0A2K5X338 | Uncharacterized protein OS=Macaca fascicularis OX=9541 GN=EGM_10375 PE=3 SV=1 | ADPRH | brown | 0.191406 | 0.084643 |
| A0A2K5X0M7 | Grancalcin OS=Macaca fascicularis OX=9541 GN=GCA PE=4 SV=2 | GCA | brown | 0.458217 | 0.478724 |
| A0A2K5WUU2 | Kininogen 1 OS=Macaca fascicularis OX=9541 GN=KNG1 PE=4 SV=1 | KNG1 | brown | -0.43363 | -0.47347 |
| A0A2K5WSB8 | Actin-depolymerizing factor OS=Macaca fascicularis OX=9541 GN=GSN PE=4 SV=2 | GSN | brown | -0.60038 | -0.56737 |
| A0A2K5WQL7 | Cathepsin S OS=Macaca fascicularis OX=9541 GN=CTSS PE=3 SV=2 | CTSS | brown | -0.64259 | -0.67517 |
| A0A2K5WPI4 | Keratin 10 OS=Macaca fascicularis OX=9541 GN=KRT10 PE=3 SV=2 | KRT10 | brown | 0.303932 | 0.313834 |
| A0A2K5WPG0 | Inter-alpha-trypsin inhibitor heavy chain 3 OS=Macaca fascicularis OX=9541 GN=ITIH3 PE=3 SV=2 | ITIH3 | brown | 0.426566 | 0.371638 |
| A0A2K5WNU0 | Metalloproteinase inhibitor 1 OS=Macaca fascicularis OX=9541 GN=TIMP1 PE=3 SV=1 | TIMP1 | brown | -0.22798 | -0.16319 |
| A0A2K5WNK0 | Complement C1q C chain OS=Macaca fascicularis OX=9541 GN=C1QC PE=4 SV=1 | C1QB | brown | -0.28817 | -0.28162 |
| A0A2K5WGH3 | Peptidase D OS=Macaca fascicularis OX=9541 GN=PEPD PE=3 SV=2 | PEPD | brown | -0.40013 | -0.334 |
| A0A2K5WEE9 | Complement factor H related 5 OS=Macaca fascicularis OX=9541 GN=CFHR5 PE=4 SV=2 | CFHR5 | brown | -0.11716 | -0.07514 |
| A0A2K5WAA5 | Uncharacterized protein OS=Macaca fascicularis OX=9541 PE=4 SV=2 | COL6A1 | brown | 0.191627 | 0.142977 |
| A0A2K5W920 | Complement C7 OS=Macaca fascicularis OX=9541 GN=C7 PE=3 SV=2 | C7 | brown | -0.42503 | -0.37624 |
| A0A2K5W7C7 | Vesicle amine transport 1 OS=Macaca fascicularis OX=9541 GN=VAT1 PE=3 SV=2 | VAT1 | brown | 0.291583 | 0.242121 |
| A0A2K5W719 | Ectonucleotide pyrophosphatase/phosphodiesterase 2 OS=Macaca fascicularis OX=9541 GN=ENPP2 PE=3 SV=2 | ENPP2 | brown | -0.43097 | -0.39374 |
| A0A2K5W6R3 | Uncharacterized protein OS=Macaca fascicularis OX=9541 GN=EGM_15390 PE=3 SV=1 | SAR1B | brown | 0.022858 | -0.02151 |
| A0A2K5W4E5 | Cysteine rich secretory protein 3 OS=Macaca fascicularis OX=9541 PE=3 SV=2 | CRISP3 | brown | -0.01951 | -0.05749 |
| A0A2K5VZN1 | Tubulin alpha chain OS=Macaca fascicularis OX=9541 PE=3 SV=2 | LOC102128802 | brown | 0.12666 | 0.083939 |
| A0A2K5VXP9 | Insulin like growth factor binding protein acid labile subunit OS=Macaca fascicularis OX=9541 GN=IGFALS PE=4 SV=2 | IGFALS | brown | -0.24061 | -0.33605 |
| A0A2K5VV75 | Clusterin OS=Macaca fascicularis OX=9541 GN=CLU PE=3 SV=2 | CLU | brown | -0.09897 | -0.13839 |
| A0A2K5VUF7 | Glycosyl-phosphatidylinositol-specific phospholipase D OS=Macaca fascicularis OX=9541 GN=GPLD1 PE=3 SV=2 | GPLD1 | brown | -0.16604 | -0.12862 |
| A0A2K5VTJ9 | Kallikrein B1 OS=Macaca fascicularis OX=9541 GN=KLKB1 PE=4 SV=2 | KLKB1 | brown | -0.52619 | -0.5551 |
| A0A2K5VQK4 | Protein C, inactivator of coagulation factors Va and VIIIa OS=Macaca fascicularis OX=9541 GN=PROC PE=4 SV=1 | PROC | brown | -0.585 | -0.52993 |
| A0A2K5VND9 | Insulin-like growth factor II OS=Macaca fascicularis OX=9541 GN=IGF2 PE=3 SV=2 | IGF2 | brown | -0.46922 | -0.43328 |
| A0A2K5VHK1 | Coagulation factor X OS=Macaca fascicularis OX=9541 GN=F10 PE=4 SV=1 | F10 | brown | -0.35381 | -0.32817 |
| A0A2K5VHF1 | Myosin heavy chain 4 OS=Macaca fascicularis OX=9541 GN=MYH4 PE=3 SV=2 | LOC102145458 | brown | -0.45196 | -0.37438 |
| A0A2K5VHA7 | Chitobiase OS=Macaca fascicularis OX=9541 GN=CTBS PE=3 SV=1 | CTBS | brown | -0.354 | -0.434 |
| A0A2K5VFF6 | Chemerin OS=Macaca fascicularis OX=9541 PE=4 SV=2 | RARRES2 | brown | -0.04401 | -0.06652 |
| A0A2K5VA46 | Uncharacterized protein OS=Macaca fascicularis OX=9541 PE=4 SV=2 | CFH | brown | -0.14135 | -0.13937 |
| A0A2K5V6D6 | Asporin OS=Macaca fascicularis OX=9541 PE=3 SV=2 | ASPN | brown | -0.26095 | -0.2368 |
| A0A2K5V1B0 | Proteasome subunit alpha type OS=Macaca fascicularis OX=9541 GN=EGM_19577 PE=3 SV=1 >tr\|G8F2T1\|G8F2T1_MACFA Proteasome subunit alpha type OS=Macaca fascicularis OX=9541 GN=PSMA2 PE=3 SV=1 | PSMA2 | brown | -0.62075 | -0.54896 |
| A0A2K5V0V6 | Complement factor I OS=Macaca fascicularis OX=9541 GN=CFI PE=4 SV=2 | CFI | brown | 0.190683 | 0.265277 |
| A0A2K5UYN7 | Antithrombin-III OS=Macaca fascicularis OX=9541 GN=SERPINC1 PE=3 SV=1 | SERPINC1 | brown | -0.54601 | -0.51724 |
| A0A2K5UXZ0 | Beta-1 metal-binding globulin OS=Macaca fascicularis OX=9541 GN=TF PE=3 SV=2 | LOC101866521 | brown | -0.43919 | -0.40394 |
| A0A2K5UU17 | Galectin 3 binding protein OS=Macaca fascicularis OX=9541 GN=LGALS3BP PE=4 SV=2 | LGALS3BP | brown | -0.22894 | -0.25953 |
| A0A2K5UTG9 | Prolylcarboxypeptidase OS=Macaca fascicularis OX=9541 GN=PRCP PE=3 SV=2 | PRCP | brown | 0.268199 | 0.323205 |
| A0A2K5UP30 | Xg glycoprotein (Xg blood group) OS=Macaca fascicularis OX=9541 GN=XG PE=3 SV=2 | XG | brown | -0.50879 | -0.41245 |
| A0A2K5UK62 | Immunoglobulin lambda variable 10-54 OS=Macaca fascicularis OX=9541 GN=IGLV10-54 PE=4 SV=1 | LOC107130949 | brown | -0.27341 | -0.2544 |
| A0A2K5UJY0 | Mannan binding lectin serine peptidase 2 OS=Macaca fascicularis OX=9541 GN=MASP2 PE=4 SV=2 | MASP2 | brown | -0.05017 | -0.02598 |
| A0A2K5UJC4 | Ig-like domain-containing protein OS=Macaca fascicularis OX=9541 PE=4 SV=2 | LOC107131036 | brown | 0.059878 | 0.204109 |
| A0A2K5UJ61 | Peptidoglycan recognition protein 2 OS=Macaca fascicularis OX=9541 GN=PGLYRP2 PE=3 SV=2 | PGLYRP2 | brown | -0.54109 | -0.45024 |
| A0A2K5UGR2 | Tissue factor pathway inhibitor OS=Macaca fascicularis OX=9541 GN=TFPI PE=4 SV=1 | TFPI | brown | -0.05791 | -0.05248 |
| A0A2K5UG57 | SERPIN domain-containing protein OS=Macaca fascicularis OX=9541 PE=3 SV=2 | LOC102143828 | brown | -0.13947 | -0.14977 |
| A0A2K5U9E5 | Regenerating family member 1 beta OS=Macaca fascicularis OX=9541 GN=REG1B PE=4 SV=2 | REG1B | brown | 0.085536 | 0.161839 |
| A0A2K5U867 | Coiled-coil domain containing 80 OS=Macaca fascicularis OX=9541 GN=CCDC80 PE=4 SV=2 | CCDC80 | brown | 0.217751 | 0.103518 |
| A0A2K5U852 | Peptide-methionine (S)-S-oxide reductase OS=Macaca fascicularis OX=9541 GN=PRSS55 PE=3 SV=2 | MSRA | brown | 0.455595 | 0.443037 |
| A0A2K5U2E1 | Keratinocyte differentiation associated protein OS=Macaca fascicularis OX=9541 GN=KRTDAP PE=4 SV=1 | KRTDAP | brown | -0.18818 | -0.18765 |
| A0A2K5TZY3 | C3/C5 convertase OS=Macaca fascicularis OX=9541 PE=4 SV=1 | CFB | brown | 0.054295 | 0.038918 |
| A0A2K5TZH3 | HGF activator OS=Macaca fascicularis OX=9541 GN=HGFAC PE=4 SV=2 | HGFAC | brown | -0.40952 | -0.41304 |
| A0A2K5TZC4 | Proprotein convertase 9 OS=Macaca fascicularis OX=9541 GN=PCSK9 PE=3 SV=2 | PCSK9 | brown | -0.02248 | 0.042049 |
| A0A2K5TU41 | Alpha-2-glycoprotein 1, zinc-binding OS=Macaca fascicularis OX=9541 GN=AZGP1 PE=3 SV=1 | AZGP1 | brown | -0.27735 | -0.2699 |
| A0A2K5TRN5 | Cathepsin H OS=Macaca fascicularis OX=9541 GN=CTSH PE=3 SV=1 | CTSH | brown | -0.14186 | -0.09301 |
| A0A2K5TQS8 | Microfibril associated protein 4 OS=Macaca fascicularis OX=9541 GN=MFAP4 PE=4 SV=2 | MFAP4 | brown | -0.25589 | -0.28138 |
| A0A2K5TQF8 | Carboxypeptidase OS=Macaca fascicularis OX=9541 GN=CTSA PE=3 SV=2 | CTSA | brown | -0.18461 | -0.25016 |
| A0A2K5TPD3 | Phospholipase A1 OS=Macaca fascicularis OX=9541 GN=LIPG PE=3 SV=1 | LIPG | brown | -0.28352 | -0.31229 |
| A0A2K5TJJ3 | Plasminogen OS=Macaca fascicularis OX=9541 PE=3 SV=2 | PLG | brown | -0.43704 | -0.39419 |
| A0A172R2I3 | Rearranged Ig variable region VDJ (Fragment) OS=Macaca fascicularis OX=9541 GN=IGH PE=2 SV=1 | LOC107130516 | brown | 0.593671 | 0.505417 |
| A0A059TDH3 | Microtubule-associated protein (Fragment) OS=Macaca fascicularis OX=9541 PE=2 SV=1 | MAP4 | brown | 0.362626 | 0.342372 |
| Q66S50 | Mannose-binding protein C OS=Macaca fascicularis OX=9541 GN=MBL2 PE=3 SV=1 >tr\|G7PDG1\|G7PDG1_MACFA MBP1 OS=Macaca fascicularis OX=9541 GN=EGM_18164 PE=4 SV=1 | MBL2 | brown | -0.23217 | -0.30612 |
| Q60HH4 | Acid ceramidase OS=Macaca fascicularis OX=9541 GN=ASAH1 PE=2 SV=1 | ASAH1 | brown | 0.037828 | 0.075694 |
| Q4R577 | Complement C1r subcomponent OS=Macaca fascicularis OX=9541 GN=C1R PE=2 SV=1 | C1R | brown | -0.44192 | -0.31399 |
| Q4R561 | Actin, cytoplasmic 1 OS=Macaca fascicularis OX=9541 GN=ACTB PE=2 SV=1 >tr\|Q25PW9\|Q25PW9_MACFA Brain cDNA clone: QmoA-10647, similar to human actin, beta (ACTB) OS=Macaca fascicularis OX=9541 PE=2 SV=1 | ACTB | brown | 0.320887 | 0.263308 |
| Q4R362 | Histone H4 OS=Macaca fascicularis OX=9541 GN=QtsA-19327 PE=3 SV=1 >tr\|G7NZ44\|G7NZ44_MACFA Histone H4 OS=Macaca fascicularis OX=9541 GN=EGM_13314 PE=4 SV=1 >tr\|G7P2N0\|G7P2N0_MACFA Histone H4 OS=Macaca fascicularis OX=9541 GN=EGM_03027 PE=3 SV=1 | LOC102136865 | brown | 0.052385 | 0.073618 |
| P68292 | Apolipoprotein A-I OS=Macaca fascicularis OX=9541 GN=APOA1 PE=1 SV=1 >tr\|G7PP13\|G7PP13_MACFA Uncharacterized protein OS=Macaca fascicularis OX=9541 GN=EGM_06260 PE=3 SV=1 | APOA1 | brown | -0.31395 | -0.25495 |
| P61261 | Beta-defensin 1 OS=Macaca fascicularis OX=9541 GN=DEFB1 PE=3 SV=1 >tr\|G7PCC6\|G7PCC6_MACFA Beta-defensin 1 OS=Macaca fascicularis OX=9541 GN=EGM_17054 PE=4 SV=1 | DEFB1 | brown | -0.08428 | -0.11808 |
| P58238 | Proteasome activator complex subunit 1 OS=Macaca fascicularis OX=9541 GN=PSME1 PE=2 SV=1 | PSME1 | brown | 0.391054 | 0.379344 |
| P0DTQ3 | Apolipoprotein C-I, acidic form OS=Macaca fascicularis OX=9541 GN=APOC1A PE=3 SV=1 | APOC1 | brown | -0.17147 | -0.24329 |
| A2V9Z4 | Albumin OS=Macaca fascicularis OX=9541 GN=ALB PE=2 SV=1 | ALB | brown | -0.51291 | -0.48445 |

**Supplementary Table 8 All the proteins contained within the green block**

| Protein accession | Protein description | Gene name | Module | Abnormality_rate | Vertebral_abnomaly |
| --- | --- | --- | --- | --- | --- |
| Q4R5G4 | Phosphoglycerate mutase OS=Macaca fascicularis OX=9541 PE=2 SV=1 | PGAM1 | green | 0.078984 | -0.0303 |
| I7G937 | Macaca fascicularis brain cDNA clone: QorA-10439, similar to human ras homolog gene family, member G (rho G) (RHOG), mRNA, RefSeq: NM_001665.2 OS=Macaca fascicularis OX=9541 PE=2 SV=1 | RHOG | green | -0.28233 | -0.33138 |
| I7G6Q2 | Macaca fascicularis brain cDNA clone: QflA-20976, similar to human nuclear transport factor 2 (NUTF2), mRNA, RefSeq: NM_005796.1 OS=Macaca fascicularis OX=9541 PE=2 SV=1 | NUTF2 | green | 0.174114 | 0.13798 |
| G7Q103 | Apoptosis-associated speck-like protein containing a CARD OS=Macaca fascicularis OX=9541 GN=PYCARD PE=4 SV=1 | PYCARD | green | -0.36715 | -0.47983 |
| G7PN21 | Ig-like domain-containing protein (Fragment) OS=Macaca fascicularis OX=9541 GN=EGM_05152 PE=4 SV=1 | LOC102125630 | green | 0.152547 | 0.105878 |
| G7PCC8 | Defensin_propep domain-containing protein OS=Macaca fascicularis OX=9541 GN=EGM_17057 PE=4 SV=1 | LOC102116821 | green | 0.141675 | 0.157266 |
| G7PC40 | Adipocyte-type fatty acid-binding protein OS=Macaca fascicularis OX=9541 GN=FABP4 PE=3 SV=1 | FABP4 | green | -0.32343 | -0.40452 |
| G7PBD8 | Serpin family A member 1 OS=Macaca fascicularis OX=9541 GN=SERPINA1 PE=3 SV=2 | SERPINA1 | green | -0.04706 | -0.01767 |
| G7PBA1 | Fibrillin-1 (Fragment) OS=Macaca fascicularis OX=9541 GN=EGM_15956 PE=3 SV=1 | FBN1 | green | -0.03376 | -0.08273 |
| A0A7N9I9H1 | Mannan binding lectin serine peptidase 1 OS=Macaca fascicularis OX=9541 GN=MASP1 PE=4 SV=1 | MASP1 | green | 0.137339 | 0.048794 |
| A0A7N9DEC3 | Myotrophin OS=Macaca fascicularis OX=9541 PE=4 SV=1 | MTPN | green | -0.08875 | -0.16226 |
| A0A7N9D9C2 | Beta-hexosaminidase OS=Macaca fascicularis OX=9541 PE=3 SV=1 | HEXA | green | 0.38485 | 0.3183 |
| A0A7N9D817 | Peptidylprolyl isomerase OS=Macaca fascicularis OX=9541 PE=4 SV=1 | FKBP1A | green | 0.046847 | 0.01424 |
| A0A7N9D5L8 | Uncharacterized protein OS=Macaca fascicularis OX=9541 PE=4 SV=1 | LOC102133501 | green | 0.019905 | -0.0507 |
| A0A7N9CYX6 | 1,4-beta-N-acetylmuramidase C OS=Macaca fascicularis OX=9541 GN=LYZ PE=3 SV=1 | LYZ | green | -0.04756 | -0.1122 |
| A0A7N9CK20 | Chloride intracellular channel protein OS=Macaca fascicularis OX=9541 PE=3 SV=1 | CLIC1 | green | -0.03363 | -0.13481 |
| A0A7N9CAJ2 | Antioxidant 1 copper chaperone OS=Macaca fascicularis OX=9541 PE=4 SV=1 | ATOX1 | green | 0.008034 | -0.13823 |
| A0A2K5WY73 | Actin-like protein 3 OS=Macaca fascicularis OX=9541 GN=ACTR3 PE=3 SV=2 | ACTR3 | green | 0.076443 | -0.08389 |
| A0A2K5WWI5 | LIM domain containing preferred translocation partner in lipoma OS=Macaca fascicularis OX=9541 GN=LPP PE=4 SV=2 | LPP | green | 0.25662 | 0.160613 |
| A0A2K5WSY0 | Actin-related protein 2/3 complex subunit OS=Macaca fascicularis OX=9541 GN=EGM_12378 PE=3 SV=1 | ARPC1B | green | -0.09575 | -0.09244 |
| A0A2K5WBF8 | Uncharacterized protein OS=Macaca fascicularis OX=9541 PE=4 SV=1 | CALM2 | green | -0.12909 | -0.18692 |
| A0A2K5WBB8 | Protein S100 OS=Macaca fascicularis OX=9541 GN=EGM_01112 PE=3 SV=2 | S100A4 | green | 0.054887 | -0.08149 |
| A0A2K5W5Z4 | ADAM metallopeptidase with thrombospondin type 1 motif 2 OS=Macaca fascicularis OX=9541 GN=ADAMTS2 PE=4 SV=2 | ADAMTS2 | green | 0.501358 | 0.48989 |
| A0A2K5W354 | Alcohol dehydrogenase [NADP(+)] OS=Macaca fascicularis OX=9541 GN=EGM_19776 PE=3 SV=1 >tr\|G8F2B0\|G8F2B0_MACFA Alcohol dehydrogenase [NADP(+)] OS=Macaca fascicularis OX=9541 GN=AKR1A1 PE=3 SV=1 | AKR1A1 | green | -0.17786 | -0.29394 |
| A0A2K5W341 | Vasodilator stimulated phosphoprotein OS=Macaca fascicularis OX=9541 GN=VASP PE=3 SV=2 | VASP | green | -0.22282 | -0.28919 |
| A0A2K5VUJ7 | Collagen type I alpha 2 chain OS=Macaca fascicularis OX=9541 GN=COL1A2 PE=4 SV=2 | COL1A2 | green | -0.16432 | -0.23831 |
| A0A2K5VU17 | Profilin OS=Macaca fascicularis OX=9541 GN=PFN1 PE=3 SV=1 | PFN1 | green | 0.053512 | -0.05119 |
| A0A2K5VRT7 | BH3-interacting domain death agonist OS=Macaca fascicularis OX=9541 GN=BID PE=4 SV=2 | BID | green | 0.231495 | 0.230637 |
| A0A2K5VR08 | Elongation factor 1-alpha OS=Macaca fascicularis OX=9541 GN=EEF1A1 PE=3 SV=1 | EEF1A1 | green | -0.55244 | -0.61361 |
| A0A2K5VQR0 | PEDS1-UBE2V1 readthrough OS=Macaca fascicularis OX=9541 GN=PEDS1-UBE2V1 PE=4 SV=2 | LOC102136723 | green | -0.16231 | -0.25262 |
| A0A2K5VK55 | ADAM metallopeptidase domain 15 OS=Macaca fascicularis OX=9541 GN=ADAM15 PE=4 SV=2 | ADAM15 | green | -0.20515 | -0.23486 |
| A0A2K5VFN9 | Collagen type VI alpha 3 chain OS=Macaca fascicularis OX=9541 GN=COL6A3 PE=4 SV=2 | COL6A3 | green | 0.389762 | 0.273879 |
| A0A2K5VE10 | DEFENSIN domain-containing protein OS=Macaca fascicularis OX=9541 PE=3 SV=2 | LOC102117659 | green | 0.155498 | 0.10786 |
| A0A2K5VCN4 | Fibulin-1 OS=Macaca fascicularis OX=9541 GN=FBLN1 PE=3 SV=2 | FBLN1 | green | 0.261742 | 0.230921 |
| A0A2K5VBH6 | N-ribosyldihydronicotinamide:quinone reductase 2 OS=Macaca fascicularis OX=9541 GN=NQO2 PE=3 SV=2 | NQO2 | green | -0.00842 | -0.19021 |
| A0A2K5V394 | Eukaryotic translation initiation factor 5A OS=Macaca fascicularis OX=9541 GN=EIF5A2 PE=3 SV=1 | EIF5A2 | green | -0.00713 | -0.08609 |
| A0A2K5US96 | Dicarbonyl and L-xylulose reductase OS=Macaca fascicularis OX=9541 GN=DCXR PE=4 SV=1 | DCXR | green | -0.15337 | -0.16317 |
| A0A2K5UKC6 | Cortactin OS=Macaca fascicularis OX=9541 GN=CTTN PE=4 SV=1 | CTTN | green | 0.112787 | 0.069925 |
| A0A2K5UJ92 | Ig-like domain-containing protein OS=Macaca fascicularis OX=9541 PE=4 SV=2 | LOC102126660 | green | -0.01905 | -0.08963 |
| A0A2K5UF24 | Peptidyl-prolyl cis-trans isomerase OS=Macaca fascicularis OX=9541 PE=3 SV=2 | LOC102135013 | green | -0.14055 | -0.25216 |
| A0A2K5UDS3 | APC-binding protein EB1 OS=Macaca fascicularis OX=9541 GN=MAPRE1 PE=3 SV=1 | MAPRE1 | green | -0.13337 | -0.193 |
| A0A2K5UCI8 | Ribonuclease A family member k6 OS=Macaca fascicularis OX=9541 GN=RNASE6 PE=3 SV=2 | RNASE6 | green | -0.22531 | -0.12128 |
| A0A2K5UCF7 | H1.5 linker histone, cluster member OS=Macaca fascicularis OX=9541 GN=H1-5 PE=3 SV=1 | LOC102138750 | green | -0.5717 | -0.63979 |
| A0A2K5U9C9 | Leukotriene A(4) hydrolase OS=Macaca fascicularis OX=9541 GN=EGM_03637 PE=3 SV=1 | LTA4H | green | 0.038033 | 0.04828 |
| A0A2K5U5S6 | Sorting nexin 12 OS=Macaca fascicularis OX=9541 GN=SNX12 PE=4 SV=1 | SNX12 | green | 0.136902 | 0.095002 |
| A0A2K5U530 | Glutaredoxin OS=Macaca fascicularis OX=9541 GN=GLRX PE=4 SV=1 | GLRX | green | 0.017808 | -0.05635 |
| A0A2K5U3F1 | Serine peptidase inhibitor Kazal type 7 OS=Macaca fascicularis OX=9541 GN=EGM_15523 PE=4 SV=1 | SPINK7 | green | 0.088395 | 0.118659 |
| A0A2K5U3B4 | 2,3-bisphosphoglycerate 3-phosphatase OS=Macaca fascicularis OX=9541 GN=MINPP1 PE=3 SV=2 | MINPP1 | green | 0.384571 | 0.299573 |
| A0A2K5U2F3 | Enhancer of rudimentary homolog OS=Macaca fascicularis OX=9541 GN=ERH PE=3 SV=1 | ERH | green | -0.1222 | -0.13262 |
| A0A2K5TZD4 | Uncharacterized protein OS=Macaca fascicularis OX=9541 PE=4 SV=2 | TNXB | green | 0.107099 | 0.097169 |
| A0A2K5TYP0 | Actin alpha 1, skeletal muscle OS=Macaca fascicularis OX=9541 GN=ACTA1 PE=3 SV=1 | ACTA1 | green | 0.23026 | 0.170836 |
| A0A2K5TYI9 | Filamin C OS=Macaca fascicularis OX=9541 GN=FLNC PE=3 SV=2 | FLNC | green | -0.24796 | -0.16787 |
| A0A2K5TTB9 | Serpin family B member 1 OS=Macaca fascicularis OX=9541 GN=SERPINB1 PE=3 SV=2 | SERPINB1 | green | -0.12647 | -0.14482 |
| A0A2K5TQK4 | Peptidase S1 domain-containing protein OS=Macaca fascicularis OX=9541 GN=EGM_21267 PE=4 SV=1 >tr\|G8F672\|G8F672_MACFA Cathepsin G OS=Macaca fascicularis OX=9541 GN=CTSG PE=4 SV=1 | CTSG | green | 0.113647 | 0.045299 |
| A0A2K5TPY2 | Isoamyl acetate-hydrolyzing esterase 1 homolog OS=Macaca fascicularis OX=9541 GN=IAH1 PE=3 SV=2 | IAH1 | green | -0.14091 | -0.19914 |
| A0A2K5TPB8 | Microtubule actin crosslinking factor 1 OS=Macaca fascicularis OX=9541 GN=MACF1 PE=4 SV=2 | MACF1 | green | 0.25866 | 0.360149 |
| A0A2K5TNF5 | Uncharacterized protein OS=Macaca fascicularis OX=9541 PE=4 SV=2 | MYL6 | green | -0.22685 | -0.29216 |
| A0A172R2G2 | Rearranged Ig variable region VDJ (Fragment) OS=Macaca fascicularis OX=9541 GN=IGH PE=2 SV=1 | LOC102127638 | green | -0.30577 | -0.42936 |
| A0A0A7KUP9 | Glyceraldehyde-3-phosphate dehydrogenase OS=Macaca fascicularis OX=9541 GN=GAPDH PE=2 SV=1 | GAPDH | green | -0.22644 | -0.30499 |
| Q4R7H5 | Elongation factor 1-gamma OS=Macaca fascicularis OX=9541 GN=EEF1G PE=2 SV=1 | EEF1G | green | 0.178341 | 0.121948 |
| Q4R549 | Macrophage migration inhibitory factor OS=Macaca fascicularis OX=9541 GN=MIF PE=3 SV=3 | MIF | green | 0.054973 | -0.00439 |
| Q4R4T5 | Heat shock protein HSP 90-beta OS=Macaca fascicularis OX=9541 GN=HSP90AB1 PE=2 SV=1 | HSP90AB1 | green | 0.108133 | 0.029396 |
| P68223 | Hemoglobin subunit beta OS=Macaca fascicularis OX=9541 GN=HBB PE=1 SV=2 >tr\|G7PQY3\|G7PQY3_MACFA Hemoglobin beta chain OS=Macaca fascicularis OX=9541 GN=EGM_05960 PE=2 SV=1 | HBB | green | -0.27653 | -0.39079 |
| P61273 | Dynein light chain 1, cytoplasmic OS=Macaca fascicularis OX=9541 GN=DYNLL1 PE=3 SV=1 >tr\|G7PJ46\|G7PJ46_MACFA Dynein light chain OS=Macaca fascicularis OX=9541 GN=EGM_03826 PE=2 SV=1 | DYNLL1 | green | -0.06988 | -0.17394 |

**Supplementary Table 9 All the proteins contained within the grey block**

| Protein accession | Protein description | Gene name | Module | Abnormality_rate | Vertebral_abnomaly |
| --- | --- | --- | --- | --- | --- |
| Q6XMM6 | C4 (Fragment) OS=Macaca fascicularis OX=9541 PE=4 SV=1 | LOC102132156 | grey | -0.17317 | -0.13613 |
| I7GHL6 | Macaca fascicularis brain cDNA clone: QorA-13574, similar to human prosaposin (variant Gaucher disease and variantmetachromatic leukodystrophy) (PSAP), mRNA, RefSeq: NM_002778.1 OS=Macaca fascicularis OX=9541 PE=2 SV=1 | PSAP | grey | -0.20025 | -0.17124 |
| G8F4T5 | Ig-like domain-containing protein (Fragment) OS=Macaca fascicularis OX=9541 GN=EGM_20593 PE=4 SV=1 | LOC102139499 | grey | 0.199138 | 0.235044 |
| G8F2W7 | Ig-like domain-containing protein (Fragment) OS=Macaca fascicularis OX=9541 GN=EGM_19665 PE=4 SV=1 | LOC102116858 | grey | -0.14851 | -0.09069 |
| G8F2W6 | Ig-like domain-containing protein (Fragment) OS=Macaca fascicularis OX=9541 GN=EGM_19664 PE=4 SV=1 | LOC107130946 | grey | -0.05578 | -0.04007 |
| G7PNJ9 | Matrix metallopeptidase 7 OS=Macaca fascicularis OX=9541 GN=MMP7 PE=3 SV=1 | MMP7 | grey | 0.215437 | 0.211768 |
| G7PN18 | Ig-like domain-containing protein (Fragment) OS=Macaca fascicularis OX=9541 GN=EGM_05149 PE=4 SV=1 | LOC102146186 | grey | -0.08545 | 0.045561 |
| G7PN14 | Ig-like domain-containing protein (Fragment) OS=Macaca fascicularis OX=9541 GN=EGM_05145 PE=4 SV=1 | LOC102131786 | grey | -0.09598 | -0.06284 |
| G7PHC3 | Ig-like domain-containing protein (Fragment) OS=Macaca fascicularis OX=9541 GN=EGM_02491 PE=4 SV=1 | LOC107131036 | grey | 0.257722 | 0.315052 |
| G7PGB2 | WNT1-inducible-signaling pathway protein 2 OS=Macaca fascicularis OX=9541 GN=EGM_02060 PE=3 SV=1 | WISP2 | grey | -0.33575 | -0.40628 |
| G7P8Z3 | Ig-like domain-containing protein (Fragment) OS=Macaca fascicularis OX=9541 GN=EGM_17025 PE=4 SV=1 | LOC102143051 | grey | 0.183169 | 0.14291 |
| G7P639 | Aminopeptidase OS=Macaca fascicularis OX=9541 GN=ENPEP PE=3 SV=1 | ENPEP | grey | 0.220893 | 0.28324 |
| G7P4U1 | ShKT domain-containing protein OS=Macaca fascicularis OX=9541 GN=EGM_13676 PE=3 SV=1 | CRISP3 | grey | 0.345397 | 0.366823 |
| G7P3F6 | Triggering receptor expressed on myeloid cells like 1 OS=Macaca fascicularis OX=9541 GN=TREML1 PE=4 SV=1 | TREML1 | grey | -0.15151 | -0.16917 |
| A0A7N9IH33 | Carboxypeptidase N subunit 2 OS=Macaca fascicularis OX=9541 GN=CPN2 PE=4 SV=1 | CPN2 | grey | -0.03342 | 0.024151 |
| A0A7N9IAI0 | Uncharacterized protein OS=Macaca fascicularis OX=9541 PE=3 SV=1 | SPRR1B | grey | 0.065972 | 0.055111 |
| A0A7N9IA06 | Guanine nucleotide-binding protein subunit gamma OS=Macaca fascicularis OX=9541 PE=3 SV=1 | GNG2 | grey | -0.23025 | -0.20525 |
| A0A7N9D829 | Ig-like domain-containing protein OS=Macaca fascicularis OX=9541 PE=4 SV=1 | LOC102127903 | grey | -0.07226 | -0.07179 |
| A0A7N9D5G1 | Ig-like domain-containing protein OS=Macaca fascicularis OX=9541 PE=4 SV=1 | LOC102139381 | grey | -0.19037 | -0.24843 |
| A0A7N9D2T7 | Ig-like domain-containing protein OS=Macaca fascicularis OX=9541 PE=4 SV=1 | LOC102121852 | grey | -0.11195 | -0.068 |
| A0A7N9D1Q3 | Coactosin like F-actin binding protein 1 OS=Macaca fascicularis OX=9541 PE=4 SV=1 | COTL1 | grey | -0.26039 | -0.24074 |
| A0A7N9D0T2 | Ig-like domain-containing protein OS=Macaca fascicularis OX=9541 PE=4 SV=1 | LOC102125506 | grey | -0.18918 | -0.14949 |
| A0A7N9CZ15 | Carbonic anhydrase OS=Macaca fascicularis OX=9541 GN=CA13 PE=3 SV=1 | CA13 | grey | 0.217525 | 0.089239 |
| A0A7N9CNK8 | Ig-like domain-containing protein OS=Macaca fascicularis OX=9541 PE=4 SV=1 | LOC102121852 | grey | 0.005172 | 0.044889 |
| A0A7N9CN86 | Ig-like domain-containing protein OS=Macaca fascicularis OX=9541 PE=4 SV=1 | LOC102130108 | grey | 0.171318 | 0.009184 |
| A0A7N9CME3 | Ig-like domain-containing protein OS=Macaca fascicularis OX=9541 PE=4 SV=1 | LOC102121852 | grey | 0.192046 | 0.203721 |
| A0A7N9CIU8 | Ig-like domain-containing protein OS=Macaca fascicularis OX=9541 PE=4 SV=1 | LOC107130290 | grey | -0.22032 | -0.22046 |
| A0A7N9CHQ9 | Ig-like domain-containing protein OS=Macaca fascicularis OX=9541 PE=4 SV=1 | LOC102143672 | grey | 0.207005 | 0.258448 |
| A0A7N9CED2 | Ig-like domain-containing protein OS=Macaca fascicularis OX=9541 PE=4 SV=1 | LOC102121852 | grey | -0.06939 | -0.06038 |
| A0A2K5X079 | Ig-like domain-containing protein OS=Macaca fascicularis OX=9541 PE=4 SV=2 | LOC102139381 | grey | 0.036967 | 0.068181 |
| A0A2K5WXY8 | 78 kDa glucose-regulated protein OS=Macaca fascicularis OX=9541 GN=HSPA5 PE=3 SV=1 | HSPA5 | grey | -0.35128 | -0.42017 |
| A0A2K5WVZ7 | C-C motif chemokine OS=Macaca fascicularis OX=9541 PE=3 SV=2 | CCL19 | grey | 0.139878 | 0.153602 |
| A0A2K5WVL1 | Hemopexin OS=Macaca fascicularis OX=9541 GN=EGM_05923 PE=3 SV=1 | HPX | grey | 0.194574 | 0.309337 |
| A0A2K5WPR4 | Lipocln_cytosolic_FA-bd_dom domain-containing protein OS=Macaca fascicularis OX=9541 PE=3 SV=2 | ORM1 | grey | 0.120637 | 0.192555 |
| A0A2K5WLE6 | Lactotransferrin OS=Macaca fascicularis OX=9541 GN=LTF PE=3 SV=2 | LTF | grey | -0.16055 | -0.16177 |
| A0A2K5WJV8 | Mitogen-activated protein kinase 14 OS=Macaca fascicularis OX=9541 PE=3 SV=1 | MAPK14 | grey | -0.07842 | -0.01322 |
| A0A2K5WID3 | Fibrinogen like 2 OS=Macaca fascicularis OX=9541 GN=FGL2 PE=4 SV=1 | FGL2 | grey | 0.040117 | -0.00775 |
| A0A2K5WGT1 | Alpha-mannosidase OS=Macaca fascicularis OX=9541 GN=MAN2B2 PE=3 SV=2 | MAN2B2 | grey | -0.29271 | -0.30161 |
| A0A2K5WG79 | Hepcidin antimicrobial peptide OS=Macaca fascicularis OX=9541 GN=HAMP PE=3 SV=1 | HAMP | grey | 0.245941 | 0.172604 |
| A0A2K5WD70 | Beta-hexosaminidase OS=Macaca fascicularis OX=9541 PE=3 SV=1 | HEXB | grey | -0.02025 | -0.09139 |
| A0A2K5W8U4 | Matrix Gla protein OS=Macaca fascicularis OX=9541 GN=EGM_03033 PE=3 SV=1 | MGP | grey | 0.098793 | 0.032428 |
| A0A2K5W8S8 | Ig-like domain-containing protein OS=Macaca fascicularis OX=9541 PE=4 SV=2 | LOC102120376 | grey | -0.00104 | -0.05888 |
| A0A2K5W6U2 | ADAMTS like 4 OS=Macaca fascicularis OX=9541 GN=ADAMTSL4 PE=4 SV=2 | ADAMTSL4 | grey | 0.187616 | 0.134107 |
| A0A2K5W3S5 | Complement component C6 OS=Macaca fascicularis OX=9541 GN=C6 PE=3 SV=2 | C6 | grey | -0.07377 | -0.03715 |
| A0A2K5VZG8 | Protein Z, vitamin K dependent plasma glycoprotein OS=Macaca fascicularis OX=9541 GN=PROZ PE=4 SV=2 | PROZ | grey | 0.003302 | -0.16302 |
| A0A2K5VSA3 | Neural cell adhesion molecule 1 OS=Macaca fascicularis OX=9541 GN=NCAM1 PE=4 SV=2 | NCAM1 | grey | 0.050175 | 0.041307 |
| A0A2K5VRK7 | Dermatopontin OS=Macaca fascicularis OX=9541 GN=DPT PE=3 SV=1 | DPT | grey | 0.062074 | 0.074382 |
| A0A2K5VGF4 | Fibroblast activation protein alpha OS=Macaca fascicularis OX=9541 GN=FAP PE=4 SV=1 | FAP | grey | 0.07686 | 0.086096 |
| A0A2K5VCH9 | SERPIN domain-containing protein OS=Macaca fascicularis OX=9541 GN=EGM_16912 PE=3 SV=1 | SERPINA1 | grey | 0.472804 | 0.501322 |
| A0A2K5V5J5 | Glucosamine-6-phosphate isomerase OS=Macaca fascicularis OX=9541 GN=EGM_15489 PE=3 SV=1 | GNPDA1 | grey | 0.199303 | 0.199416 |
| A0A2K5UWC7 | Ig-like domain-containing protein OS=Macaca fascicularis OX=9541 PE=4 SV=2 | LOC102141705 | grey | -0.08234 | -0.15678 |
| A0A2K5UQY2 | DEFSN domain-containing protein OS=Macaca fascicularis OX=9541 PE=4 SV=2 | LOC102124095 | grey | -0.07511 | -0.08432 |
| A0A2K5UQG2 | Immunoglobulin lambda variable 4-69 OS=Macaca fascicularis OX=9541 GN=IGLV4-69 PE=4 SV=2 | LOC102121507 | grey | -0.42891 | -0.37966 |
| A0A2K5UMM8 | Ig-like domain-containing protein OS=Macaca fascicularis OX=9541 PE=4 SV=2 | LOC102120376 | grey | -0.12227 | -0.07573 |
| A0A2K5UJ20 | Tsukushi, small leucine rich proteoglycan OS=Macaca fascicularis OX=9541 GN=TSKU PE=4 SV=1 | TSKU | grey | -0.06897 | -0.02753 |
| A0A2K5UIU2 | Ig-like domain-containing protein OS=Macaca fascicularis OX=9541 PE=4 SV=2 | LOC107131035 | grey | 0.12938 | 0.216586 |
| A0A2K5UIL6 | Ig-like domain-containing protein OS=Macaca fascicularis OX=9541 PE=4 SV=2 | LOC107128712 | grey | -0.0094 | -0.03772 |
| A0A2K5UI48 | Ig-like domain-containing protein OS=Macaca fascicularis OX=9541 PE=4 SV=2 | LOC102140263 | grey | -0.1709 | -0.15603 |
| A0A2K5UI25 | Folate gamma-glutamyl hydrolase OS=Macaca fascicularis OX=9541 GN=GGH PE=3 SV=2 | GGH | grey | 0.140346 | 0.132613 |
| A0A2K5UCE0 | Cadherin-2 OS=Macaca fascicularis OX=9541 PE=4 SV=2 | CDH2 | grey | -0.04931 | -0.05451 |
| A0A2K5UBN1 | Ig-like domain-containing protein OS=Macaca fascicularis OX=9541 PE=4 SV=2 | LOC107127076 | grey | -0.0902 | -0.14085 |
| A0A2K5U8W4 | Ig-like domain-containing protein OS=Macaca fascicularis OX=9541 PE=4 SV=2 | LOC107130509 | grey | -0.30815 | -0.27798 |
| A0A2K5U3L8 | Complement C8 alpha chain OS=Macaca fascicularis OX=9541 GN=C8A PE=3 SV=2 | C8A | grey | -0.2524 | -0.27495 |
| A0A2K5U3C2 | Secretory leukocyte peptidase inhibitor OS=Macaca fascicularis OX=9541 GN=SLPI PE=4 SV=2 | SLPI | grey | 0.281455 | 0.171732 |
| A0A2K5U3A1 | Fumarylacetoacetase OS=Macaca fascicularis OX=9541 GN=FAH PE=3 SV=1 | FAH | grey | 0.084503 | 0.012031 |
| A0A2K5TWI4 | Ficolin 1 OS=Macaca fascicularis OX=9541 GN=FCN1 PE=4 SV=2 | FCN1 | grey | 0.070933 | 0.009612 |
| A0A2K5TWH0 | Fibrinogen C-terminal domain-containing protein OS=Macaca fascicularis OX=9541 PE=3 SV=2 | LOC102131557 | grey | 0.384029 | 0.361082 |
| A0A2K5TVE8 | Tubulointerstitial nephritis antigen like 1 OS=Macaca fascicularis OX=9541 GN=TINAGL1 PE=3 SV=2 | TINAGL1 | grey | 0.005717 | -0.08572 |
| A0A2K5TUI8 | Ryanodine receptor 2 OS=Macaca fascicularis OX=9541 GN=RYR2 PE=4 SV=2 | RYR2 | grey | -0.28282 | -0.40506 |
| A0A2K5TMG7 | Cellular communication network factor 2 OS=Macaca fascicularis OX=9541 GN=CCN2 PE=3 SV=1 | CTGF | grey | 0.172202 | 0.248064 |
| A0A2K5TJB2 | Transforming growth factor beta OS=Macaca fascicularis OX=9541 GN=TGFB1 PE=3 SV=2 | TGFB1 | grey | 0.252344 | 0.323549 |
| A0A172R2I7 | Rearranged Ig variable region VDJ (Fragment) OS=Macaca fascicularis OX=9541 GN=IGH PE=2 SV=1 | LOC107130512 | grey | 0.111218 | 0.064719 |
| A0A172R2H4 | Rearranged Ig variable region VDJ (Fragment) OS=Macaca fascicularis OX=9541 GN=IGH PE=2 SV=1 | LOC102143051 | grey | 0.183808 | 0.168938 |
| A0A172R2G7 | Rearranged Ig variable region VDJ (Fragment) OS=Macaca fascicularis OX=9541 GN=IGH PE=2 SV=1 | LOC102124047 | grey | -0.21494 | -0.2619 |
| A0A172R2G5 | Rearranged Ig variable region VDJ (Fragment) OS=Macaca fascicularis OX=9541 GN=IGH PE=2 SV=1 | LOC107130516 | grey | 0.476743 | 0.527434 |
| A0A172R2G3 | Rearranged Ig variable region VDJ (Fragment) OS=Macaca fascicularis OX=9541 GN=IGH PE=2 SV=1 | LOC102136084 | grey | 0.276881 | 0.393219 |
| Q4R6C4 | N(4)-(beta-N-acetylglucosaminyl)-L-asparaginase OS=Macaca fascicularis OX=9541 GN=AGA PE=2 SV=1 | AGA | grey | 0.103149 | 0.021584 |
| Q4R5P2 | ADP-ribosylation factor 1 OS=Macaca fascicularis OX=9541 GN=ARF1 PE=2 SV=3 >tr\|A0A158SIP9\|A0A158SIP9_MACFA Brain cDNA clone: QbsB-10485, similar to human ADP-ribosylation factor 1 (ARF1) OS=Macaca fascicularis OX=9541 PE=2 SV=1 | ARF1 | grey | 0.342792 | 0.264556 |
| Q4R379 | Ras-related protein Ral-B OS=Macaca fascicularis OX=9541 GN=RALB PE=2 SV=1 | RALB | grey | -0.26239 | -0.38702 |

**Supplementary Table 10 All the proteins contained within the turquoise block**

| Protein accession | Protein description | Gene name | Module | Abnormality_rate | Vertebral_abnomaly |
| --- | --- | --- | --- | --- | --- |
| Q4R5L3 | Glutathione transferase OS=Macaca fascicularis OX=9541 GN=GSTM5 PE=2 SV=1 | GSTM5 | turquoise | -0.03997 | -0.05666 |
| I7GPF3 | Serpin family F member 1 OS=Macaca fascicularis OX=9541 PE=2 SV=1 | SERPINF1 | turquoise | -0.32277 | -0.36066 |
| I7GIP6 | Heat shock 27 kDa protein OS=Macaca fascicularis OX=9541 PE=2 SV=1 | HSPB1 | turquoise | 0.098241 | 0.1006 |
| G8F4W8 | Uncharacterized protein (Fragment) OS=Macaca fascicularis OX=9541 GN=EGM_20643 PE=4 SV=1 | LOC102128706 | turquoise | 0.327534 | 0.366225 |
| G8F4J6 | Uncharacterized protein (Fragment) OS=Macaca fascicularis OX=9541 GN=EGM_20443 PE=4 SV=1 | LOC102116308 | turquoise | 0.238578 | 0.26956 |
| G8F425 | Ig-like domain-containing protein (Fragment) OS=Macaca fascicularis OX=9541 GN=EGM_20220 PE=4 SV=1 | LOC107131036 | turquoise | 0.205991 | 0.219491 |
| G8F3M1 | Macaca fascicularis brain cDNA clone: QorA-12578, similar to human ras homolog gene family, member A (RHOA), mRNA, RefSeq: NM_001664.1 OS=Macaca fascicularis OX=9541 PE=2 SV=1 >tr\|I7GLB2\|I7GLB2_MACFA Uncharacterized protein OS=Macaca fascicularis OX=9541 GN=EGM_20036 PE=4 SV=1 | RHOC | turquoise | 0.171583 | 0.118034 |
| G8F2X0 | Ig-like domain-containing protein (Fragment) OS=Macaca fascicularis OX=9541 GN=EGM_19668 PE=4 SV=1 | LOC107130949 | turquoise | -0.03489 | -0.02898 |
| G7Q1F6 | Phosphatidylcholine-sterol acyltransferase OS=Macaca fascicularis OX=9541 GN=EGM_11866 PE=3 SV=1 | LCAT | turquoise | 0.109373 | 0.068483 |
| G7PZZ1 | Chondroitinsulfatase OS=Macaca fascicularis OX=9541 GN=EGM_12064 PE=3 SV=1 | GALNS | turquoise | 0.386584 | 0.328512 |
| G7PZZ0 | Adenine phosphoribosyltransferase OS=Macaca fascicularis OX=9541 GN=EGM_12063 PE=3 SV=1 | APRT | turquoise | 0.178125 | 0.190139 |
| G7PTW8 | Serum-spreading factor OS=Macaca fascicularis OX=9541 GN=EGM_07543 PE=4 SV=1 | VTN | turquoise | 0.064117 | 0.062629 |
| G7PT00 | Alpha-2-antiplasmin OS=Macaca fascicularis OX=9541 GN=EGM_07232 PE=3 SV=1 | SERPINF2 | turquoise | 0.30963 | 0.266907 |
| G7PQA7 | Prothrombin OS=Macaca fascicularis OX=9541 GN=EGM_05674 PE=3 SV=1 | F2 | turquoise | 0.287251 | 0.325749 |
| G7PN19 | Ig-like domain-containing protein (Fragment) OS=Macaca fascicularis OX=9541 GN=EGM_05150 PE=4 SV=1 | LOC102146186 | turquoise | 0.22044 | 0.216253 |
| G7PMP5 | Ig-like domain-containing protein OS=Macaca fascicularis OX=9541 GN=EGM_05005 PE=4 SV=1 | LOC102129829 | turquoise | 0.050284 | -0.02241 |
| G7PMP4 | Ig-like domain-containing protein OS=Macaca fascicularis OX=9541 GN=EGM_05004 PE=4 SV=1 | LOC102129829 | turquoise | 0.051362 | 0.019858 |
| G7PJR5 | Alpha-2-macroglobulin OS=Macaca fascicularis OX=9541 GN=EGM_02953 PE=3 SV=1 | LOC102130734 | turquoise | 0.139587 | 0.187984 |
| G7PHG6 | Uncharacterized protein OS=Macaca fascicularis OX=9541 GN=EGM_02547 PE=4 SV=1 | KNG1 | turquoise | -0.17396 | -0.13748 |
| G7PHD1 | Uncharacterized protein OS=Macaca fascicularis OX=9541 GN=EGM_02501 PE=4 SV=1 | LOC102136297 | turquoise | 0.200716 | 0.202326 |
| G7P9F5 | Aminopeptidase OS=Macaca fascicularis OX=9541 GN=EGM_16277 PE=3 SV=1 | ANPEP | turquoise | -0.06819 | -0.05564 |
| G7P8Z4 | Ig-like domain-containing protein (Fragment) OS=Macaca fascicularis OX=9541 GN=EGM_17026 PE=4 SV=1 | LOC102140263 | turquoise | 0.043941 | 0.076252 |
| G7P8R2 | Osteonectin OS=Macaca fascicularis OX=9541 GN=SPARC PE=3 SV=1 | SPARC | turquoise | 0.00409 | -0.00248 |
| G7P8L5 | Serine peptidase inhibitor Kazal type 1 OS=Macaca fascicularis OX=9541 GN=SPINK1 PE=4 SV=1 | SPINK1 | turquoise | -0.44254 | -0.4869 |
| G7P5K8 | C-X-C motif chemokine OS=Macaca fascicularis OX=9541 GN=EGM_14416 PE=3 SV=1 | LOC102125111 | turquoise | 0.433674 | 0.496889 |
| G7P1C5 | Paraoxonase OS=Macaca fascicularis OX=9541 GN=PON1 PE=3 SV=1 | PON1 | turquoise | 0.162639 | 0.087396 |
| G7NYL9 | Uncharacterized protein OS=Macaca fascicularis OX=9541 GN=EGM_10805 PE=4 SV=1 | HRG | turquoise | 0.102392 | 0.143695 |
| G7NX90 | Intelectin 1 OS=Macaca fascicularis OX=9541 GN=ITLN1 PE=4 SV=2 | ITLN1 | turquoise | -0.22278 | -0.17386 |
| D4P3V7 | Carboxylic ester hydrolase OS=Macaca fascicularis OX=9541 GN=BChE PE=2 SV=1 | BCHE | turquoise | -0.04723 | 0.035114 |
| C0SJM5 | Glutathione S-transferase omega OS=Macaca fascicularis OX=9541 GN=GSTO1 PE=2 SV=1 | GSTO1 | turquoise | -0.18098 | -0.16232 |
| A2V9Z3 | Uncharacterized protein OS=Macaca fascicularis OX=9541 PE=2 SV=1 | APOE | turquoise | 0.440779 | 0.452284 |
| A0A805RCF6 | Uncharacterized protein OS=Macaca fascicularis OX=9541 PE=3 SV=1 | APOA1 | turquoise | 0.098976 | 0.096001 |
| A0A7N9IH16 | Ig-like domain-containing protein OS=Macaca fascicularis OX=9541 PE=4 SV=1 | LOC102146186 | turquoise | 0.314033 | 0.347614 |
| A0A7N9IFT8 | Ig-like domain-containing protein OS=Macaca fascicularis OX=9541 PE=4 SV=1 | LOC102128716 | turquoise | 0.21981 | 0.243042 |
| A0A7N9IDU3 | Serum amyloid A protein OS=Macaca fascicularis OX=9541 PE=3 SV=1 | LOC102141725 | turquoise | 0.341923 | 0.37351 |
| A0A7N9ID62 | Uncharacterized protein OS=Macaca fascicularis OX=9541 PE=4 SV=1 | LOC102136297 | turquoise | 0.172849 | 0.178617 |
| A0A7N9ID58 | Carboxypeptidase N subunit 1 OS=Macaca fascicularis OX=9541 GN=CPN1 PE=3 SV=1 | CPN1 | turquoise | -0.15195 | -0.17376 |
| A0A7N9ICP2 | Ig-like domain-containing protein OS=Macaca fascicularis OX=9541 PE=4 SV=1 | LOC107127081 | turquoise | 0.154398 | 0.157153 |
| A0A7N9IB48 | Fibrinogen beta chain OS=Macaca fascicularis OX=9541 GN=FGB PE=4 SV=1 | FGB | turquoise | 0.33613 | 0.323595 |
| A0A7N9DFE4 | Inter-alpha-trypsin inhibitor heavy chain 4 OS=Macaca fascicularis OX=9541 GN=ITIH4 PE=3 SV=1 | ITIH4 | turquoise | 0.00702 | -0.00027 |
| A0A7N9DC64 | DEFENSIN domain-containing protein OS=Macaca fascicularis OX=9541 PE=3 SV=1 | LOC102126320 | turquoise | -0.09599 | -0.0578 |
| A0A7N9DB46 | Ig-like domain-containing protein OS=Macaca fascicularis OX=9541 PE=4 SV=1 | LOC102146186 | turquoise | 0.225783 | 0.210058 |
| A0A7N9D7S7 | Ig-like domain-containing protein OS=Macaca fascicularis OX=9541 PE=4 SV=1 | LOC102132176 | turquoise | -0.08015 | -0.08648 |
| A0A7N9D714 | Alpha-1,6-mannosyl-glycoprotein 2-beta-N-acetylglucosaminyltransferase OS=Macaca fascicularis OX=9541 PE=3 SV=1 | MGAT2 | turquoise | 0.586958 | 0.501118 |
| A0A7N9D6S9 | Joining chain of multimeric IgA and IgM OS=Macaca fascicularis OX=9541 GN=JCHAIN PE=4 SV=1 | JCHAIN | turquoise | 0.14439 | 0.152571 |
| A0A7N9D1R1 | Uncharacterized protein OS=Macaca fascicularis OX=9541 PE=4 SV=1 | LOC102128706 | turquoise | -0.65185 | -0.62726 |
| A0A7N9CWM1 | Ig-like domain-containing protein OS=Macaca fascicularis OX=9541 PE=4 SV=1 | LOC102142105 | turquoise | 0.01111 | -0.05339 |
| A0A7N9CWF8 | CD5 molecule like OS=Macaca fascicularis OX=9541 GN=CD5L PE=4 SV=1 | CD5L | turquoise | 0.018912 | 0.048768 |
| A0A7N9CU35 | Ig-like domain-containing protein OS=Macaca fascicularis OX=9541 PE=4 SV=1 | PXDN | turquoise | -0.24254 | -0.23684 |
| A0A7N9CSJ1 | Hedgehog protein OS=Macaca fascicularis OX=9541 GN=IHH PE=3 SV=1 | IHH | turquoise | 0.50297 | 0.565152 |
| A0A7N9CRX1 | Ig-like domain-containing protein OS=Macaca fascicularis OX=9541 PE=4 SV=1 | LOC107130519 | turquoise | 0.373341 | 0.417909 |
| A0A7N9CR72 | Ig-like domain-containing protein OS=Macaca fascicularis OX=9541 PE=4 SV=1 | LOC107127071 | turquoise | 0.121084 | 0.010893 |
| A0A7N9CQQ8 | Uncharacterized protein OS=Macaca fascicularis OX=9541 PE=4 SV=1 | LOC102142558 | turquoise | 0.23695 | 0.227114 |
| A0A7N9CQF5 | Ig-like domain-containing protein OS=Macaca fascicularis OX=9541 PE=4 SV=1 | LOC102129829 | turquoise | 0.017282 | 0.024004 |
| A0A7N9CL49 | Ig-like domain-containing protein OS=Macaca fascicularis OX=9541 PE=4 SV=1 | LOC102129829 | turquoise | 0.189699 | 0.139272 |
| A0A7N9CKY8 | Kringle domain-containing protein OS=Macaca fascicularis OX=9541 PE=4 SV=1 | LPA | turquoise | -0.24576 | -0.27775 |
| A0A7N9CJI2 | RNAse_Pc domain-containing protein OS=Macaca fascicularis OX=9541 PE=3 SV=1 | ANG | turquoise | 0.448533 | 0.468037 |
| A0A7N9CJ21 | Ig-like domain-containing protein OS=Macaca fascicularis OX=9541 PE=4 SV=1 | LOC102132176 | turquoise | 0.02117 | -0.03949 |
| A0A7N9CII3 | Ig-like domain-containing protein OS=Macaca fascicularis OX=9541 PE=4 SV=1 | LOC102119103 | turquoise | -0.08936 | -0.09647 |
| A0A7N9CHS1 | GLOBIN domain-containing protein OS=Macaca fascicularis OX=9541 PE=3 SV=1 | LOC102136846 | turquoise | 0.157688 | 0.215048 |
| A0A7N9CH45 | Thioredoxin OS=Macaca fascicularis OX=9541 PE=3 SV=1 | TXN | turquoise | 0.086049 | 0.043255 |
| A0A7N9CH04 | Macrophage stimulating 1 OS=Macaca fascicularis OX=9541 PE=3 SV=1 | MST1 | turquoise | 0.579882 | 0.572962 |
| A0A7N9CEB8 | Ig-like domain-containing protein OS=Macaca fascicularis OX=9541 PE=4 SV=1 | LOC107131040 | turquoise | -0.10818 | -0.01031 |
| A0A7N9CA59 | Endogenous retrovirus group MER34 member 1, envelope OS=Macaca fascicularis OX=9541 GN=ERVMER34-1 PE=4 SV=1 | ERVMER34-1 | turquoise | -0.09973 | -0.0926 |
| A0A7N9C6X8 | Ig-like domain-containing protein OS=Macaca fascicularis OX=9541 PE=4 SV=1 | LOC102139010 | turquoise | 0.05934 | 0.151095 |
| A0A650AXN4 | Complement factor I OS=Macaca fascicularis OX=9541 GN=CFI PE=2 SV=1 | CFI | turquoise | 0.481871 | 0.559398 |
| A0A2K5X2W7 | Complement component C9 OS=Macaca fascicularis OX=9541 GN=C9 PE=3 SV=1 | C9 | turquoise | 0.446473 | 0.475216 |
| A0A2K5X2N6 | Alpha-1-B glycoprotein OS=Macaca fascicularis OX=9541 GN=A1BG PE=4 SV=2 | A1BG | turquoise | 0.138685 | 0.177096 |
| A0A2K5X2M3 | Uncharacterized protein OS=Macaca fascicularis OX=9541 PE=4 SV=2 | LOC102129539 | turquoise | 0.090592 | 0.111756 |
| A0A2K5X2M0 | Keratin 16 OS=Macaca fascicularis OX=9541 GN=KRT16 PE=3 SV=2 | KRT16 | turquoise | -0.01034 | 0.00578 |
| A0A2K5X046 | Ubiquitin conjugating enzyme E2 L3 OS=Macaca fascicularis OX=9541 PE=3 SV=1 | LOC102140123 | turquoise | -0.08043 | -0.07036 |
| A0A2K5WZK1 | Secretoglobin family 3A member 1 OS=Macaca fascicularis OX=9541 GN=SCGB3A1 PE=4 SV=1 | SCGB3A1 | turquoise | 0.00877 | 0.115446 |
| A0A2K5WZA2 | Insulin-like growth factor-binding protein 2 OS=Macaca fascicularis OX=9541 GN=IGFBP2 PE=4 SV=2 | IGFBP2 | turquoise | -0.17427 | -0.13258 |
| A0A2K5WYX9 | Uncharacterized protein OS=Macaca fascicularis OX=9541 PE=4 SV=2 | CFH | turquoise | 0.470949 | 0.548672 |
| A0A2K5WY01 | Ig-like domain-containing protein OS=Macaca fascicularis OX=9541 PE=4 SV=2 | LOC107127072 | turquoise | 0.252402 | 0.175423 |
| A0A2K5WSY1 | Serpin family A member 7 OS=Macaca fascicularis OX=9541 GN=SERPINA7 PE=3 SV=2 | SERPINA7 | turquoise | 0.152752 | 0.245774 |
| A0A2K5WRJ5 | Hephaestin like 1 OS=Macaca fascicularis OX=9541 GN=HEPHL1 PE=3 SV=2 | HEPHL1 | turquoise | 0.013859 | 0.040986 |
| A0A2K5WRC0 | ABRA C-terminal-like protein OS=Macaca fascicularis OX=9541 GN=EGM_13967 PE=3 SV=1 | ABRACL | turquoise | 0.099376 | 0.050966 |
| A0A2K5WR32 | Inter-alpha-trypsin inhibitor heavy chain 2 OS=Macaca fascicularis OX=9541 GN=ITIH2 PE=3 SV=2 | ITIH2 | turquoise | 0.080675 | 0.115867 |
| A0A2K5WNV2 | C1q domain-containing protein OS=Macaca fascicularis OX=9541 GN=EGM_00326 PE=4 SV=1 | C1QC | turquoise | 0.206007 | 0.212147 |
| A0A2K5WNU6 | Serpin family F member 2 OS=Macaca fascicularis OX=9541 GN=SERPINF2 PE=3 SV=2 | SERPINF2 | turquoise | 0.205754 | 0.216107 |
| A0A2K5WHY8 | Hyaluronan binding protein 2 OS=Macaca fascicularis OX=9541 GN=HABP2 PE=4 SV=2 | HABP2 | turquoise | 0.1248 | 0.056603 |
| A0A2K5WFC8 | GLI pathosis related 2 OS=Macaca fascicularis OX=9541 PE=4 SV=1 | GLIPR2 | turquoise | 0.134447 | 0.13571 |
| A0A2K5WEE7 | Uncharacterized protein OS=Macaca fascicularis OX=9541 GN=EGM_21014 PE=4 SV=1 >tr\|G8F5N7\|G8F5N7_MACFA Complement factor H related 5 OS=Macaca fascicularis OX=9541 GN=CFHR5 PE=4 SV=1 | LOC102134603 | turquoise | 0.161946 | 0.15391 |
| A0A2K5WDQ7 | Lecithin-cholesterol acyltransferase OS=Macaca fascicularis OX=9541 GN=LCAT PE=3 SV=2 | LCAT | turquoise | 0.199373 | 0.207739 |
| A0A2K5WCM3 | ADAM metallopeptidase domain 10 OS=Macaca fascicularis OX=9541 GN=ADAM10 PE=4 SV=2 | ADAM10 | turquoise | 0.110367 | 0.085143 |
| A0A2K5WAC5 | Uncharacterized protein OS=Macaca fascicularis OX=9541 PE=4 SV=2 | LOC102132156 | turquoise | 0.307397 | 0.306697 |
| A0A2K5W9H2 | Alpha-2-HS-glycoprotein OS=Macaca fascicularis OX=9541 GN=AHSG PE=4 SV=1 | AHSG | turquoise | -0.1533 | -0.13442 |
| A0A2K5W786 | Hyaluronidase OS=Macaca fascicularis OX=9541 GN=HYAL1 PE=3 SV=2 | HYAL1 | turquoise | 0.10018 | 0.126529 |
| A0A2K5W5C2 | Uncharacterized protein OS=Macaca fascicularis OX=9541 PE=4 SV=1 | LOC102114868 | turquoise | 0.110548 | 0.102297 |
| A0A2K5W3G8 | C-type lectin domain family 3 member B OS=Macaca fascicularis OX=9541 GN=CLEC3B PE=4 SV=1 | CLEC3B | turquoise | -0.23163 | -0.21771 |
| A0A2K5W2N5 | Uncharacterized protein OS=Macaca fascicularis OX=9541 PE=4 SV=2 | IGLL1 | turquoise | 0.037576 | 0.0158 |
| A0A2K5W172 | Retinol-binding protein OS=Macaca fascicularis OX=9541 GN=RBP4 PE=3 SV=1 | RBP4 | turquoise | -0.07869 | -0.05618 |
| A0A2K5VRN1 | Christmas factor OS=Macaca fascicularis OX=9541 GN=F9 PE=4 SV=1 | F9 | turquoise | -0.0764 | -0.09874 |
| A0A2K5VQX9 | Complement C1s OS=Macaca fascicularis OX=9541 GN=C1S PE=4 SV=2 | C1S | turquoise | -0.09761 | 0.023013 |
| A0A2K5VPK0 | Apolipoprotein C-IV OS=Macaca fascicularis OX=9541 GN=APOC4 PE=3 SV=1 | APOC4 | turquoise | 0.308354 | 0.336404 |
| A0A2K5VP36 | Ceruloplasmin OS=Macaca fascicularis OX=9541 GN=CP PE=3 SV=2 | CP | turquoise | 0.012195 | 0.049467 |
| A0A2K5VNY5 | Keratin 9 OS=Macaca fascicularis OX=9541 GN=KRT9 PE=3 SV=2 | KRT9 | turquoise | -0.46258 | -0.34881 |
| A0A2K5VES4 | Vitamin K-dependent protein S OS=Macaca fascicularis OX=9541 GN=EGM_10266 PE=4 SV=1 | PROS1 | turquoise | 0.216472 | 0.239361 |
| A0A2K5VE67 | Insulin like growth factor binding protein 4 OS=Macaca fascicularis OX=9541 GN=IGFBP4 PE=4 SV=2 | IGFBP4 | turquoise | -0.20636 | -0.21387 |
| A0A2K5VDN5 | Apolipoprotein M OS=Macaca fascicularis OX=9541 GN=EGM_13450 PE=3 SV=1 | APOM | turquoise | 0.188405 | 0.235217 |
| A0A2K5V8H1 | Coagulation factor VII OS=Macaca fascicularis OX=9541 GN=F7 PE=4 SV=1 | F7 | turquoise | 0.454205 | 0.48755 |
| A0A2K5V684 | Adipocyte plasma membrane-associated protein OS=Macaca fascicularis OX=9541 GN=APMAP PE=3 SV=1 | APMAP | turquoise | 0.506118 | 0.547837 |
| A0A2K5V414 | Out at first protein homolog OS=Macaca fascicularis OX=9541 GN=OAF PE=3 SV=2 | | turquoise | 0.242116 | 0.225976 |
| A0A2K5V365 | Coagulation factor XII OS=Macaca fascicularis OX=9541 GN=F12 PE=4 SV=2 | F12 | turquoise | -0.07476 | 0.000244 |
| A0A2K5V295 | Cadherin-1 OS=Macaca fascicularis OX=9541 GN=CDH1 PE=4 SV=1 | CDH1 | turquoise | -0.13406 | -0.1164 |
| A0A2K5V1W9 | Gc-globulin OS=Macaca fascicularis OX=9541 GN=EGM_14434 PE=4 SV=1 | GC | turquoise | -0.31646 | -0.28745 |
| A0A2K5UZ51 | Prothrombin OS=Macaca fascicularis OX=9541 GN=F2 PE=3 SV=2 | F2 | turquoise | -0.02948 | 0.003122 |
| A0A2K5UYF0 | Chondroadherin OS=Macaca fascicularis OX=9541 GN=CHAD PE=4 SV=2 | CHAD | turquoise | 0.270556 | 0.232449 |
| A0A2K5UVV0 | C-X-C motif chemokine OS=Macaca fascicularis OX=9541 GN=EGM_14415 PE=3 SV=1 | PPBP | turquoise | 0.27088 | 0.334552 |
| A0A2K5UTQ8 | Serpin family D member 1 OS=Macaca fascicularis OX=9541 GN=SERPIND1 PE=3 SV=2 | SERPIND1 | turquoise | 0.212565 | 0.276423 |
| A0A2K5URB6 | Immunoglobulin kappa constant OS=Macaca fascicularis OX=9541 GN=IGKC PE=4 SV=2 | LOC102133296 | turquoise | 0.138105 | 0.192189 |
| A0A2K5UKT4 | Ig-like domain-containing protein OS=Macaca fascicularis OX=9541 PE=4 SV=2 | LOC102121524 | turquoise | 0.283394 | 0.264003 |
| A0A2K5UK54 | Immunoglobulin lambda variable 11-55 (non-functional) OS=Macaca fascicularis OX=9541 GN=IGLV11-55 PE=4 SV=1 | LOC107126472 | turquoise | 0.284187 | 0.326608 |
| A0A2K5UK06 | Hexosyltransferase OS=Macaca fascicularis OX=9541 GN=EGM_09765 PE=3 SV=1 | B3GNT8 | turquoise | -0.0654 | -0.06902 |
| A0A2K5UJE6 | Ig-like domain-containing protein OS=Macaca fascicularis OX=9541 PE=4 SV=2 | LOC107130291 | turquoise | 0.138058 | 0.225787 |
| A0A2K5UIU0 | Ribonuclease A family member 4 OS=Macaca fascicularis OX=9541 GN=RNASE4 PE=3 SV=2 | RNASE4 | turquoise | 0.373189 | 0.344002 |
| A0A2K5UHY6 | Cytochrome c OS=Macaca fascicularis OX=9541 PE=3 SV=1 | LOC102136392 | turquoise | -0.00836 | -0.04098 |
| A0A2K5UHX3 | Superoxide dismutase [Cu-Zn] OS=Macaca fascicularis OX=9541 GN=SOD3 PE=3 SV=2 | SOD3 | turquoise | 0.088376 | 0.040102 |
| A0A2K5UHQ2 | Ig-like domain-containing protein OS=Macaca fascicularis OX=9541 PE=4 SV=2 | LOC107128639 | turquoise | 0.26285 | 0.260088 |
| A0A2K5UEZ1 | Immunoglobulin lambda variable 5-52 OS=Macaca fascicularis OX=9541 GN=IGLV5-52 PE=4 SV=1 | LOC107131031 | turquoise | 0.378934 | 0.386405 |
| A0A2K5UEU6 | Immunoglobulin kappa variable 6D-41 (non-functional) OS=Macaca fascicularis OX=9541 GN=IGKV6D-41 PE=4 SV=2 | LOC102145803 | turquoise | -0.11348 | -0.11545 |
| A0A2K5UEA5 | Nucleoside diphosphate kinase OS=Macaca fascicularis OX=9541 GN=NME2 PE=3 SV=1 | LOC102125550 | turquoise | 0.216734 | 0.186462 |
| A0A2K5UE84 | Calcium-activated neutral proteinase small subunit OS=Macaca fascicularis OX=9541 PE=4 SV=2 | LOC102131455 | turquoise | -0.2268 | -0.2268 |
| A0A2K5UC71 | Uncharacterized protein OS=Macaca fascicularis OX=9541 PE=4 SV=2 | LOC102142263 | turquoise | 0.09372 | 0.149717 |
| A0A2K5U7X5 | Exostosin like glycosyltransferase 2 OS=Macaca fascicularis OX=9541 GN=EXTL2 PE=4 SV=1 | EXTL2 | turquoise | -0.18134 | -0.17403 |
| A0A2K5TYM1 | C-C motif chemokine OS=Macaca fascicularis OX=9541 GN=CCL16 PE=3 SV=1 | CCL16 | turquoise | 0.160328 | 0.196613 |
| A0A2K5TY00 | Fibrinogen gamma chain OS=Macaca fascicularis OX=9541 GN=FGG PE=4 SV=2 | FGG | turquoise | 0.501516 | 0.520061 |
| A0A2K5TW84 | Serine/threonine-protein phosphatase OS=Macaca fascicularis OX=9541 GN=PPP1CA PE=3 SV=2 | PPP1CA | turquoise | 0.179394 | 0.148205 |
| A0A2K5TW15 | Uncharacterized protein OS=Macaca fascicularis OX=9541 PE=4 SV=2 | IGLL5 | turquoise | 0.164465 | 0.190876 |
| A0A2K5TVD2 | Sorcin OS=Macaca fascicularis OX=9541 GN=SRI PE=4 SV=2 | SRI | turquoise | 0.03866 | 0.011557 |
| A0A2K5TUG6 | Bleomycin hydrolase OS=Macaca fascicularis OX=9541 GN=BLMH PE=3 SV=1 | BLMH | turquoise | 0.104143 | -0.0077 |
| A0A2K5TT34 | Alpha-amylase OS=Macaca fascicularis OX=9541 PE=3 SV=2 | LOC102123076 | turquoise | -0.02407 | -0.0917 |
| A0A2K5TSZ9 | Alpha-amylase OS=Macaca fascicularis OX=9541 PE=3 SV=2 | LOC102123076 | turquoise | 0.012061 | -0.01259 |
| A0A2K5TSW1 | Receptor protein-tyrosine kinase OS=Macaca fascicularis OX=9541 GN=DDR2 PE=4 SV=2 | DDR2 | turquoise | 0.19144 | 0.131258 |
| A0A2K5TSM5 | 1-alkyl-2-acetylglycerophosphocholine esterase OS=Macaca fascicularis OX=9541 GN=PLA2G7 PE=4 SV=2 | PLA2G7 | turquoise | -0.04708 | -0.0092 |
| A0A2K5TS80 | Coagulation factor XIII B chain OS=Macaca fascicularis OX=9541 GN=F13B PE=4 SV=2 | F13B | turquoise | -0.16547 | -0.13757 |
| A0A2K5TRK3 | Lumican OS=Macaca fascicularis OX=9541 PE=3 SV=1 | LUM | turquoise | -0.18937 | -0.18796 |
| A0A2K5TQM4 | Serpin family A member 10 OS=Macaca fascicularis OX=9541 GN=SERPINA10 PE=3 SV=2 | SERPINA10 | turquoise | -0.33484 | -0.40596 |
| A0A2K5TPI7 | ACB domain-containing protein OS=Macaca fascicularis OX=9541 PE=4 SV=2 | DBI | turquoise | -0.01286 | -0.08697 |
| A0A2K5TP51 | Uncharacterized protein OS=Macaca fascicularis OX=9541 PE=4 SV=2 | LOC102130108 | turquoise | 0.248734 | 0.256906 |
| A0A2K5TNA6 | Apolipoprotein B OS=Macaca fascicularis OX=9541 GN=APOB PE=4 SV=1 | APOB | turquoise | 0.063706 | -0.00099 |
| A0A2K5TN82 | Apolipoprotein B OS=Macaca fascicularis OX=9541 GN=APOB PE=4 SV=2 | APOB | turquoise | -0.12615 | -0.07409 |
| A0A2K5TJG3 | Zymogen granule membrane protein 16 OS=Macaca fascicularis OX=9541 GN=EGM_11617 PE=4 SV=1 | ZG16 | turquoise | -0.19633 | -0.20452 |
| A0A2K5TJA4 | Coagulation factor V OS=Macaca fascicularis OX=9541 GN=F5 PE=3 SV=2 | F5 | turquoise | 0.168781 | 0.207192 |
| A0A2K5TJ42 | Complement component 8 subunit beta OS=Macaca fascicularis OX=9541 GN=EGM_20240 PE=3 SV=1 | C8B | turquoise | -0.01472 | 0.014168 |
| A0A172R2H8 | Rearranged Ig variable region VDJ (Fragment) OS=Macaca fascicularis OX=9541 GN=IGH PE=2 SV=1 | LOC107130516 | turquoise | 0.12832 | 0.150753 |
| A0A172R2F4 | Rearranged Ig variable region VDJ (Fragment) OS=Macaca fascicularis OX=9541 GN=IGH PE=2 SV=1 | LOC107130519 | turquoise | 0.345229 | 0.218326 |
| A0A172R2E3 | Rearranged Ig variable region VDJ (Fragment) OS=Macaca fascicularis OX=9541 GN=IGH PE=2 SV=1 | LOC102118733 | turquoise | 0.147443 | 0.059694 |
| A0A023JCN4 | Glutathione transferase OS=Macaca fascicularis OX=9541 GN=GSTT2 PE=2 SV=1 | GSTT2 | turquoise | 0.32042 | 0.178107 |
| Q9TSM5 | Glutathione S-transferase Mu 1 OS=Macaca fascicularis OX=9541 GN=GSTM1 PE=2 SV=3 | GSTM4 | turquoise | -0.02697 | -0.07973 |
| Q95KC9 | Prenylcysteine oxidase OS=Macaca fascicularis OX=9541 GN=PCYOX1 PE=2 SV=1 | PCYOX1 | turquoise | -0.27285 | -0.23748 |
| Q8SPW0 | Beta-2-microglobulin OS=Macaca fascicularis OX=9541 GN=B2M PE=3 SV=1 | B2M | turquoise | -0.06119 | -0.08692 |
| Q8SPI0 | Apolipoprotein D OS=Macaca fascicularis OX=9541 GN=APOD PE=2 SV=1 | APOD | turquoise | 0.214126 | 0.24389 |
| Q8HXW1 | Transthyretin OS=Macaca fascicularis OX=9541 GN=TTR PE=2 SV=2 >tr\|G7PWK8\|G7PWK8_MACFA Transthyretin OS=Macaca fascicularis OX=9541 GN=TTR PE=2 SV=1 | TTR | turquoise | 0.10605 | 0.127139 |
| Q6LDD9 | Hypoxanthine-guanine phosphoribosyltransferase OS=Macaca fascicularis OX=9541 GN=HPRT1 PE=2 SV=3 | PRTFDC1 | turquoise | 0.393941 | 0.385504 |
| Q60HC1 | Integral membrane protein 2B OS=Macaca fascicularis OX=9541 GN=ITM2B PE=2 SV=1 | ITM2B | turquoise | -0.17516 | -0.18808 |
| Q4R5L1 | Aspartate aminotransferase, cytoplasmic OS=Macaca fascicularis OX=9541 GN=GOT1 PE=2 SV=1 >tr\|Q25L56\|Q25L56_MACFA Aspartate aminotransferase OS=Macaca fascicularis OX=9541 PE=2 SV=1 | GOT1 | turquoise | -0.03144 | -0.05267 |
| Q4R4J2 | Coronin-1A OS=Macaca fascicularis OX=9541 GN=CORO1A PE=2 SV=3 | CORO1A | turquoise | 0.324517 | 0.296731 |
| Q1KLX7 | Cathelicidin antimicrobial peptide OS=Macaca fascicularis OX=9541 GN=CAMP PE=3 SV=1 | CAMP | turquoise | 0.081895 | 0.090869 |
| P68303 | Metallothionein-2 OS=Macaca fascicularis OX=9541 GN=MT2 PE=3 SV=1 >tr\|G7Q161\|G7Q161_MACFA Metallothionein OS=Macaca fascicularis OX=9541 GN=EGM_11753 PE=3 SV=1 | LOC102135658 | turquoise | 0.139616 | 0.121563 |
| P33621 | Apolipoprotein A-IV OS=Macaca fascicularis OX=9541 GN=APOA4 PE=2 SV=1 | APOA4 | turquoise | -0.20538 | -0.19178 |
| P28714 | Epididymal secretory glutathione peroxidase OS=Macaca fascicularis OX=9541 GN=GPX5 PE=2 SV=1 >tr\|G7P3T2\|G7P3T2_MACFA Glutathione peroxidase OS=Macaca fascicularis OX=9541 GN=EGM_13370 PE=3 SV=1 | GPX5 | turquoise | 0.195622 | 0.212329 |
| P18659 | Apolipoprotein C-III OS=Macaca fascicularis OX=9541 GN=APOC3 PE=1 SV=2 | APOC3 | turquoise | 0.210379 | 0.223009 |
| P18658 | Apolipoprotein C-II OS=Macaca fascicularis OX=9541 GN=APOC2 PE=1 SV=2 >tr\|A2V9Y5\|A2V9Y5_MACFA Apolipoprotein C-II OS=Macaca fascicularis OX=9541 PE=2 SV=1 | APOC2 | turquoise | -0.02362 | -0.00452 |
| P18657 | Apolipoprotein C-I, basic form OS=Macaca fascicularis OX=9541 GN=APOC1B PE=1 SV=2 | APOC1 | turquoise | 0.063546 | 0.042718 |
| P18656 | Apolipoprotein A-II OS=Macaca fascicularis OX=9541 GN=APOA2 PE=1 SV=2 >tr\|G7NXA8\|G7NXA8_MACFA Apolipoprotein A-II OS=Macaca fascicularis OX=9541 GN=EGM_01298 PE=3 SV=1 | APOA2 | turquoise | -0.22233 | -0.22382 |

**Supplementary Table 11 All the proteins contained within the yellow block**

| Protein accession | Protein description | Gene name | Module | Abnormality_rate | Vertebral_abnomaly |
| --- | --- | --- | --- | --- | --- |
| I7GPE6 | Carbonic anhydrase OS=Macaca fascicularis OX=9541 PE=2 SV=1 | CA2 | yellow | -0.50254 | -0.54324 |
| G7Q018 | Hemoglobin alpha chain OS=Macaca fascicularis OX=9541 GN=EGM_11265 PE=3 SV=1 | LOC102136846 | yellow | -0.5619 | -0.60212 |
| G7PU51 | C-C motif chemokine OS=Macaca fascicularis OX=9541 GN=EGM_07643 PE=3 SV=1 | LOC102134112 | yellow | -0.24434 | -0.13114 |
| G7PPF9 | Cystatin E/M OS=Macaca fascicularis OX=9541 GN=CST6 PE=4 SV=1 | CST6 | yellow | -0.54857 | -0.57499 |
| G7PN10 | Ig-like domain-containing protein (Fragment) OS=Macaca fascicularis OX=9541 GN=EGM_05141 PE=4 SV=1 | LOC102137503 | yellow | -0.67262 | -0.59135 |
| G7PHX5 | Uncharacterized protein OS=Macaca fascicularis OX=9541 GN=EGM_03325 PE=4 SV=1 | | yellow | -0.62704 | -0.62396 |
| G7P8Z1 | Ig-like domain-containing protein (Fragment) OS=Macaca fascicularis OX=9541 GN=EGM_17023 PE=4 SV=1 | LOC107130512 | yellow | 0.003166 | -0.08084 |
| G7P6F8 | Fibrinogen alpha chain OS=Macaca fascicularis OX=9541 GN=EGM_14762 PE=4 SV=1 | FGA | yellow | -0.16211 | -0.23124 |
| G7P026 | FABP domain-containing protein OS=Macaca fascicularis OX=9541 GN=EGM_11047 PE=3 SV=1 | RBP1 | yellow | -0.56445 | -0.56552 |
| G7NZV2 | Uncharacterized protein OS=Macaca fascicularis OX=9541 GN=EGM_10595 PE=3 SV=1 | ITIH1 | yellow | -0.78833 | -0.79023 |
| G7NXY9 | Beta-1 metal-binding globulin OS=Macaca fascicularis OX=9541 GN=EGM_11087 PE=3 SV=1 | LOC101866521 | yellow | -0.18384 | -0.24221 |
| C0SJM2 | Glutathione S-transferase OS=Macaca fascicularis OX=9541 GN=GSTAv1 PE=2 SV=1 | LOC102115229 | yellow | -0.57152 | -0.57553 |
| A0A7N9I9K7 | Alpha-1,3-mannosyl-glycoprotein 2-beta-N-acetylglucosaminyltransferase OS=Macaca fascicularis OX=9541 GN=MGAT1 PE=3 SV=1 | MGAT1 | yellow | 0.163365 | 0.23614 |
| A0A7N9DFI9 | Ig-like domain-containing protein OS=Macaca fascicularis OX=9541 PE=4 SV=1 | LOC102129464 | yellow | -0.33717 | -0.43607 |
| A0A7N9DD93 | Desert hedgehog signaling molecule OS=Macaca fascicularis OX=9541 GN=DHH PE=3 SV=1 | DHH | yellow | 0.357251 | 0.326659 |
| A0A7N9DA44 | Cystatin B OS=Macaca fascicularis OX=9541 GN=CSTB PE=3 SV=1 | CSTB | yellow | -0.32538 | -0.37808 |
| A0A7N9D9E2 | GLOBIN domain-containing protein OS=Macaca fascicularis OX=9541 PE=3 SV=1 | HBB | yellow | -0.33615 | -0.40102 |
| A0A7N9D6T3 | Cystatin C OS=Macaca fascicularis OX=9541 GN=CST3 PE=4 SV=1 | CST3 | yellow | -0.4499 | -0.45254 |
| A0A7N9D0A6 | GLOBIN domain-containing protein OS=Macaca fascicularis OX=9541 PE=3 SV=1 | LOC102143165 | yellow | -0.47499 | -0.5084 |
| A0A7N9CTI1 | Adenine phosphoribosyltransferase OS=Macaca fascicularis OX=9541 GN=APRT PE=3 SV=1 | APRT | yellow | 0.000732 | -0.09767 |
| A0A7N9CNI5 | Immunoglobulin lambda variable 5-52 OS=Macaca fascicularis OX=9541 GN=IGLV5-52 PE=4 SV=1 | LOC102116858 | yellow | 0.62357 | 0.569743 |
| A0A7N9CLP1 | GLOBIN domain-containing protein OS=Macaca fascicularis OX=9541 PE=3 SV=1 | LOC102136192 | yellow | -0.46254 | -0.4844 |
| A0A7N9CID1 | Transforming growth factor-beta-induced protein ig-h3 OS=Macaca fascicularis OX=9541 GN=TGFBI PE=4 SV=1 | TGFBI | yellow | -0.26608 | -0.29571 |
| A0A7N9CGS4 | Uncharacterized protein OS=Macaca fascicularis OX=9541 PE=4 SV=1 | LOC102132137 | yellow | -0.69906 | -0.7498 |
| A0A7N9CDP7 | Uncharacterized protein OS=Macaca fascicularis OX=9541 PE=3 SV=1 | LOC101867369 | yellow | -0.69685 | -0.69185 |
| A0A7N9CAD5 | Trefoil factor 2 OS=Macaca fascicularis OX=9541 PE=4 SV=1 | TFF2 | yellow | -0.44997 | -0.45817 |
| A0A7N9C838 | Ig-like domain-containing protein OS=Macaca fascicularis OX=9541 PE=4 SV=1 | LOC102135686 | yellow | -0.3654 | -0.51592 |
| A0A2K5X8C8 | Sex hormone binding globulin OS=Macaca fascicularis OX=9541 PE=4 SV=2 | SHBG | yellow | -0.25982 | -0.25078 |
| A0A2K5X3G2 | LIM zinc-binding domain-containing protein OS=Macaca fascicularis OX=9541 PE=4 SV=1 | CRIP1 | yellow | -0.47248 | -0.52227 |
| A0A2K5WZX3 | Biliverdin reductase B OS=Macaca fascicularis OX=9541 GN=BLVRB PE=4 SV=2 | BLVRB | yellow | -0.55532 | -0.57174 |
| A0A2K5WXU9 | C-X-C motif chemokine 12 OS=Macaca fascicularis OX=9541 PE=3 SV=2 | CXCL12 | yellow | -0.06983 | -0.14024 |
| A0A2K5WWG8 | Glucagon OS=Macaca fascicularis OX=9541 GN=GCG PE=3 SV=2 | GCG | yellow | -0.27173 | -0.35242 |
| A0A2K5WN50 | Immunoglobulin kappa variable 1-27 OS=Macaca fascicularis OX=9541 GN=IGKV1-27 PE=4 SV=2 | LOC102125630 | yellow | 0.14365 | 0.264473 |
| A0A2K5WN42 | Ig-like domain-containing protein OS=Macaca fascicularis OX=9541 PE=4 SV=2 | LOC102142104 | yellow | 0.140038 | 0.2262 |
| A0A2K5WJ10 | GLOBIN domain-containing protein OS=Macaca fascicularis OX=9541 PE=3 SV=1 | LOC102143165 | yellow | -0.24019 | -0.30102 |
| A0A2K5WIF5 | GLOBIN domain-containing protein OS=Macaca fascicularis OX=9541 PE=3 SV=2 | LOC102136846 | yellow | -0.54241 | -0.54345 |
| A0A2K5VZI6 | Mannosyl-oligosaccharide glucosidase OS=Macaca fascicularis OX=9541 GN=MOGS PE=3 SV=2 | MOGS | yellow | 0.273799 | 0.346292 |
| A0A2K5VZ08 | Malate dehydrogenase 1 OS=Macaca fascicularis OX=9541 GN=MDH1 PE=4 SV=1 | MDH1 | yellow | -0.35047 | -0.41779 |
| A0A2K5VUR4 | Chitinase OS=Macaca fascicularis OX=9541 GN=CHIT1 PE=3 SV=2 | CHIT1 | yellow | -0.26497 | -0.20232 |
| A0A2K5VRI0 | Ankyrin 1 OS=Macaca fascicularis OX=9541 GN=ANK1 PE=4 SV=2 | ANK1 | yellow | -0.40226 | -0.40751 |
| A0A2K5VHQ4 | Neuropeptide Y OS=Macaca fascicularis OX=9541 GN=NPY PE=3 SV=1 | NPY | yellow | -0.2213 | -0.22941 |
| A0A2K5VGX1 | Uncharacterized protein OS=Macaca fascicularis OX=9541 GN=EGM_18009 PE=4 SV=1 | C9H10orf99 | yellow | -0.00954 | -0.04467 |
| A0A2K5VE94 | Alpha glucosidase OS=Macaca fascicularis OX=9541 GN=GAA PE=3 SV=2 | GAA | yellow | -0.26705 | -0.29446 |
| A0A2K5V8Q3 | Selenium binding protein 1 OS=Macaca fascicularis OX=9541 GN=SELENBP1 PE=3 SV=2 | SELENBP1 | yellow | -0.69717 | -0.77827 |
| A0A2K5V619 | Thrombospondin 1 OS=Macaca fascicularis OX=9541 PE=3 SV=2 | THBS1 | yellow | 0.114603 | 0.209251 |
| A0A2K5V2H1 | Chromogranin A OS=Macaca fascicularis OX=9541 GN=CHGA PE=3 SV=2 | CHGA | yellow | -0.66551 | -0.68026 |
| A0A2K5UV95 | Calcium-regulated heat-stable protein of 24 kDa OS=Macaca fascicularis OX=9541 GN=EGM_11465 PE=4 SV=1 | CARHSP1 | yellow | -0.61873 | -0.64256 |
| A0A2K5UV39 | SPARC-like protein 1 OS=Macaca fascicularis OX=9541 GN=SPARCL1 PE=3 SV=2 | SPARCL1 | yellow | -0.35045 | -0.3218 |
| A0A2K5UU44 | Prosaposin OS=Macaca fascicularis OX=9541 GN=PSAP PE=4 SV=2 | PSAP | yellow | -0.35229 | -0.30418 |
| A0A2K5UTN5 | SH3 domain binding glutamate rich protein like 3 OS=Macaca fascicularis OX=9541 GN=SH3BGRL3 PE=3 SV=1 | SH3BGRL3 | yellow | -0.44559 | -0.52785 |
| A0A2K5UTI1 | Ig-like domain-containing protein OS=Macaca fascicularis OX=9541 PE=4 SV=2 | LOC107130949 | yellow | -0.29827 | -0.22744 |
| A0A2K5USW4 | Acylphosphatase OS=Macaca fascicularis OX=9541 PE=3 SV=2 | ACYP1 | yellow | -0.48857 | -0.49422 |
| A0A2K5URZ6 | Serine peptidase inhibitor Kazal type 5 OS=Macaca fascicularis OX=9541 GN=SPINK5 PE=4 SV=2 | SPINK5 | yellow | -0.27958 | -0.2867 |
| A0A2K5URP2 | Ig-like domain-containing protein OS=Macaca fascicularis OX=9541 PE=4 SV=2 | LOC102128311 | yellow | -0.38297 | -0.30993 |
| A0A2K5URA2 | Complement C3d receptor 2 OS=Macaca fascicularis OX=9541 GN=CR2 PE=4 SV=2 | CR2 | yellow | 0.416389 | 0.353963 |
| A0A2K5UML5 | Peroxiredoxin 1 OS=Macaca fascicularis OX=9541 GN=EGM_00603 PE=3 SV=1 | PRDX1 | yellow | 0.013316 | -0.02185 |
| A0A2K5UJA3 | Purine nucleoside phosphorylase OS=Macaca fascicularis OX=9541 PE=3 SV=2 | PNP | yellow | -0.27704 | -0.3732 |
| A0A2K5UIH5 | Papilin, proteoglycan like sulfated glycoprotein OS=Macaca fascicularis OX=9541 GN=PAPLN PE=4 SV=2 | PAPLN | yellow | -0.30451 | -0.34952 |
| A0A2K5UEU7 | Ig-like domain-containing protein OS=Macaca fascicularis OX=9541 PE=4 SV=2 | LOC102129829 | yellow | -0.65024 | -0.5875 |
| A0A2K5UDV1 | Nucleoside diphosphate kinase OS=Macaca fascicularis OX=9541 GN=NME1 PE=3 SV=2 | LOC101925770 | yellow | -0.34507 | -0.39636 |
| A0A2K5UD97 | B-cell maturation protein OS=Macaca fascicularis OX=9541 GN=EGM_11488 PE=4 SV=1 | TNFRSF17 | yellow | -0.47419 | -0.51651 |
| A0A2K5U7W5 | C-type lectin domain-containing protein OS=Macaca fascicularis OX=9541 PE=4 SV=2 | LOC102119426 | yellow | -0.35434 | -0.45078 |
| A0A2K5U5Y1 | Proteasome activator subunit 2 OS=Macaca fascicularis OX=9541 PE=3 SV=2 | PSME2 | yellow | -0.25926 | -0.24443 |
| A0A2K5U233 | Galectin OS=Macaca fascicularis OX=9541 GN=EGM_16650 PE=4 SV=1 | LGALS3 | yellow | 0.194864 | 0.256031 |
| A0A2K5U228 | Regenerating family member 3 alpha OS=Macaca fascicularis OX=9541 GN=REG3A PE=4 SV=1 | LOC102140919 | yellow | -0.16993 | -0.14589 |
| A0A2K5U090 | Regenerating family member 1 alpha OS=Macaca fascicularis OX=9541 GN=REG1A PE=4 SV=2 | LOC102141707 | yellow | -0.7689 | -0.73057 |
| A0A2K5TWI0 | Fibrinogen C-terminal domain-containing protein OS=Macaca fascicularis OX=9541 PE=3 SV=2 | LOC102132200 | yellow | 0.631943 | 0.642425 |
| A0A2K5TTS9 | Zinc finger CCCH-type containing, antiviral 1 OS=Macaca fascicularis OX=9541 GN=ZC3HAV1 PE=3 SV=2 | ZC3HAV1 | yellow | -0.05029 | 0.083919 |
| A0A2K5TMC4 | Macrophage colony-stimulating factor 1 OS=Macaca fascicularis OX=9541 GN=CSF1 PE=4 SV=2 | CSF1 | yellow | -0.18263 | -0.21354 |
| A0A2K5TKE8 | Chromogranin B OS=Macaca fascicularis OX=9541 GN=CHGB PE=3 SV=2 | CHGB | yellow | -0.55357 | -0.63286 |
| A0A2K5TJ43 | Platelet-derived growth factor D OS=Macaca fascicularis OX=9541 GN=PDGFD PE=3 SV=2 | PDGFD | yellow | 0.139233 | 0.15714 |
| Q8HXQ1 | Superoxide dismutase [Cu-Zn] OS=Macaca fascicularis OX=9541 GN=SOD1 PE=2 SV=3 | SOD1 | yellow | -0.57088 | -0.62934 |
| Q4R6L7 | Bisphosphoglycerate mutase OS=Macaca fascicularis OX=9541 GN=BPGM PE=2 SV=3 | BPGM | yellow | -0.18664 | -0.26722 |
| Q4R5H0 | Protein-L-isoaspartate(D-aspartate) O-methyltransferase OS=Macaca fascicularis OX=9541 GN=PCMT1 PE=2 SV=3 | LOC102122412 | yellow | -0.33207 | -0.39315 |
| Q4R5F2 | Lactoylglutathione lyase OS=Macaca fascicularis OX=9541 GN=GLO1 PE=2 SV=3 >tr\|G7P3D8\|G7P3D8_MACFA Lactoylglutathione lyase OS=Macaca fascicularis OX=9541 GN=EGM_13562 PE=3 SV=1 | GLO1 | yellow | -0.35285 | -0.39239 |
| Q4R4T8 | Legumain OS=Macaca fascicularis OX=9541 GN=LGMN PE=2 SV=1 | LGMN | yellow | -0.44868 | -0.47179 |
| Q2PFZ3 | Peroxiredoxin-2 OS=Macaca fascicularis OX=9541 GN=PRDX2 PE=2 SV=3 | PRDX2 | yellow | -0.47707 | -0.52619 |
| Q2PFL9 | Peroxiredoxin-6 OS=Macaca fascicularis OX=9541 GN=PRDX6 PE=2 SV=3 | RPF1 | yellow | -0.06477 | -0.10414 |
| P61918 | NPC intracellular cholesterol transporter 2 OS=Macaca fascicularis OX=9541 GN=NPC2 PE=2 SV=1 | NPC2 | yellow | -0.59623 | -0.69254 |
| P0C273 | Ubiquitin-60S ribosomal protein L40 OS=Macaca fascicularis OX=9541 GN=UBA52 PE=2 SV=2 | UBA52 | yellow | -0.31655 | -0.39357 |
| P02150 | Myoglobin OS=Macaca fascicularis OX=9541 GN=MB PE=1 SV=2 >tr\|G7PFA9\|G7PFA9_MACFA Myoglobin OS=Macaca fascicularis OX=9541 GN=EGM_02630 PE=3 SV=1 | MB | yellow | -0.42513 | -0.4328 |
